# Adjuvant trastuzumab emtansine in HER2-positive breast cancer patients with HER2-negative residual invasive disease in KATHERINE

**DOI:** 10.1038/s41523-022-00477-z

**Published:** 2022-09-19

**Authors:** Sibylle Loibl, Chiun-Sheng Huang, Max S. Mano, Eleftherios P. Mamounas, Charles E. Geyer, Michael Untch, Jean-Christophe Thery, Ingo Schwaner, Steven Limentani, Niklas Loman, Kristina Lübbe, Jenny C. Chang, Thomas Hatschek, David Tesarowski, Chunyan Song, Sanne Lysbet de Haas, Thomas Boulet, Chiara Lambertini, Norman Wolmark

**Affiliations:** 1 GBG, Neu-Isenburg, Germany; 2 Centre for Haematology and Oncology Bethanien, Frankfurt, Germany; 3 National Taiwan University Hospital and National Taiwan University College of Medicine, Taipei, Taiwan; 4 Instituto do Câncer do Estado de São Paulo, São Paulo, Brazil; 5 NSABP Foundation and Orlando Health Cancer Institute, Orlando, FL USA; 6 NSABP Foundation and University of Pittsburgh Hillman Cancer Center, Pittsburgh, PA USA; 7 AGO-B and HELIOS Klinikum Berlin Buch, Berlin, Germany; 8 Centre Henri Becquerel, Cancer Center, Rouen, France; 9 Oncological Practice, Berlin, Germany; 10 Atrium Health, Charlotte, NC USA; 11 Skåne University Hospital, Lund, Sweden; 12 Diakovere Henrietten Stift, Hannover, Germany; 13 Houston Methodist Cancer Center, Houston, TX USA; 14 Karolinska University Hospital, Solna, Sweden; 15 Genentech, Inc., South San Francisco, CA USA; 16 F. Hoffmann-La Roche Ltd, Basel, Switzerland; 17 NSABP Foundation and University of Pittsburgh, Pittsburgh, PA USA

**Keywords:** Breast cancer, Breast cancer, Outcomes research

## Abstract

Following chemotherapy and human epidermal growth factor 2 (HER2)-targeted neoadjuvant therapy for HER2-positive early breast cancer, residual invasive breast cancer at surgery may be HER2-negative on retesting in some patients. We evaluated outcomes with T-DM1 and trastuzumab in patients randomized in the phase III KATHERINE trial based on HER2-positive central testing of the pre-treatment core biopsy with HER2-negative central testing on their corresponding surgical specimen after neoadjuvant treatment. In the 70/845 (8.3%) patients with HER2-negative residual disease on retesting at surgery, there were 11 IDFS events in the 42 trastuzumab-treated patients (26.2%) and none in the 28 T-DM1-treated patients, suggesting that T-DM1 should not be withheld in this patient population.

Patients with human epidermal growth factor receptor 2 (HER2)-positive early breast cancer (EBC) and residual invasive disease at surgery, after chemotherapy and HER2-targeted neoadjuvant systemic therapy (NAST), have higher rates of recurrence and death than those attaining a pathological complete response^1,2^. In the phase 3 KATHERINE study, adjuvant trastuzumab emtansine (T-DM1) reduced the risk of invasive disease recurrence or death by 50% compared with adjuvant trastuzumab in these high-risk patients^3^, and changed the standard of care for this patient population^4,5^.

Changes from HER2-positive to HER2-negative status in residual breast cancer after NAST have been documented^6–10^, but how this affects outcomes with subsequent adjuvant treatment remains unclear. This is of potential importance with T-DM1, a HER2-targeted antibody-drug conjugate. In this descriptive report we provide available outcome information in KATHERINE patients with HER2-negative status on re-testing of residual disease after NAST.

Paired pre-NAST (core biopsy) and post-NAST (surgical) tumor samples were available for 1002 of the 1486 patients enrolled in KATHERINE, and valid, centrally determined HER2 status results were available in 845 paired samples (invalid/unknown in 157) (Fig. 1a). Pre-NAST samples were preferentially used to assess eligibility and were prospectively confirmed to be HER2-positive. Of the assessable paired post-NAST residual disease samples, 775 (91.7%) were HER2-positive and 70 (8.3%) were HER2-negative. The HER2-negative subgroup consisted of 53 patients with HER2-negative status by both IHC and ISH. We also considered 17 additional patients with IHC 0–1+ and unknown ISH to be HER2-negative given the high likelihood of IHC 0–1+ being ISH-negative^11^. The rate of HER2 status conversion in KATHERINE is somewhat less than previous series reporting changes to HER2-negative status after HER2-targeted NAST in 13–32% of patients, however, the patient populations differ in some respects^6,7,10^.

**Fig. 1:**
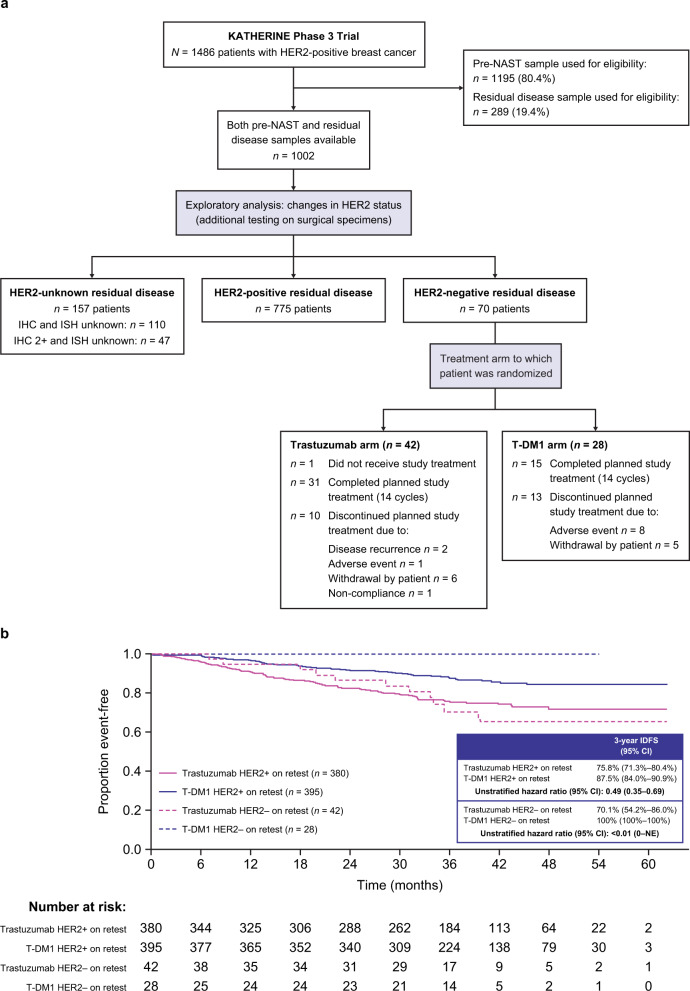
Analysis of outcomes in patients with HER2-negative vs HER2-positive residual disease upon retesting at surgery. **a** Patient data assessed for this analysis is shown. Of the 1486 enrolled patients, two in the trastuzumab arm were excluded from the analysis, one without centrally confirmed HER2-positive breast cancer and another who was inadvertently randomized twice. Paired pre-NAST and residual disease samples were available for 845 patients. Patients with HER2-negative residual disease (*n* = 70) include 53 with confirmed HER2-negative disease and 17 with unknown HER2-status (IHC 0–1+/ISH unknown). **b** Kaplan–Meier estimates of time to first IDFS event in each treatment arm are shown. Abbreviations: CI confidence interval, HER2 human epidermal growth factor receptor 2, IDFS invasive disease-free survival, NAST neoadjuvant systemic therapy, NE not estimable, and T-DM1 trastuzumab emtansine.

To determine whether the 70 patients with HER2-negative residual disease could also be distinguished by baseline characteristics, we compared characteristics between these patients and those maintaining HER2-positive residual disease. Baseline age, sex, race, clinical stage at presentation, and tumor hormone receptor status were similar between the two groups (Table 1). However, the pre-NAST samples of patients with HER2-negative residual disease had increased percentages with HER2 1+ and 2+ staining intensity, gene ratios <4 and heterogenous HER2 protein expression than those who maintained HER2-positive residual disease (Table 1). Patients with HER2-negative residual disease were also more likely to have had residual disease corresponding to pathological stage I disease at surgery (47.1% vs 38.8%; Supplementary Table 1) and to have received dual HER2-targeted NAST (30.0% vs 17.9%; Table 1).

**Table 1:** Baseline characteristics by HER2 status in residual disease at surgery after neoadjuvant systemic therapy.

Characteristic, No. (%)^a^	All (*N* = 1486)	Patients with residual disease assessed as HER2-positive on retest (*n* = 775)	Patients with residual disease assessed as HER2-negative upon retest		
			All (*n* = 70)	Trastuzumab (*n* = 42)	T-DM1 (*n* = 28)
Sample used for study eligibility^b^					
Presurgical	1195 (80.4)	775 (100)	70 (100)	42 (100)	28 (100)
Surgical	289 (19.4)	0	0	0	0
Median age (range), y	49 (23–80)	49 (23–80)	49 (27–73)	48.5 (27–73)	52 (33–73)
Sex					
Female	1481 (99.7)	773 (99.7)	70 (100)	42 (100)	28 (100)
Male	5 (0.3)	2 (0.3)	0	0	0
Race^c^					
White	1082 (72.8)	581 (75.0)	54 (77.1)	33 (78.6)	21 (75.0)
Asian	129 (8.7)	68 (8.8)	5 (7.1)	2 (4.8)	3 (10.7)
American Indian, Alaska Native, or Pacific Islander	87 (5.9)	35 (4.5)	3 (4.3)	3 (7.1)	0
Black or African American	40 (2.7)	20 (2.6)	2 (2.9)	1 (2.4)	1 (3.6)
Multiple/Unknown	148 (10.0)	71 (9.2)	6 (8.6)	3 (7.1)	3 (10.7)
Clinical stage at presentation					
Operable	1111 (74.8)	590 (76.1)	50 (71.4)	31 (73.8)	19 (67.9)
Inoperable	375 (25.2)	185 (23.9)	20 (28.6)	11 (26.2)	9 (22.1)
Hormone receptor status					
ER-negative and PgR-negative/unknown	412 (27.7)	196 (25.3)	17 (24.3)	11 (26.2)	6 (21.4)
ER- and/or PgR-positive	1074 (72.3)	579 (74.7)	53 (75.7)	31 (73.8)	22 (78.6)
Neoadjuvant HER2-targeted therapy					
Trastuzumab alone	1196 (80.5)	636 (82.1)	49 (70.0)	27 (64.3)	22 (78.6)
Trastuzumab + additional HER2-targeted agent	290 (19.5)	139 (17.9)	21 (30.0)	15 (35.7)	6 (21.4)
HER2 status by IHC at eligibility screening^d^					
IHC0/1+	25 (1.7)	11 (1.4)	3 (4.3)	2 (4.8)	1 (3.6)
IHC2+	326 (21.9)	136 (17.5)	25 (35.7)	16 (38.1)	9 (32.1)
IHC3+	1132 (76.2)	627 (80.9)	42 (60.0)	24 (57.1)	18 (64.3)
Unknown	3 (0.2)	1 (0.1)	0	0	0
*HER2* gene ratio at eligibility screening^d^					
<2	11 (0.7)	8 (1.0)	1 (1.4)	0	1 (3.6)
2 to <4	422 (28.4)	197 (25.4)	29 (41.4)	19 (45.2)	10 (35.7)
≥4	982 (66.1)	540 (69.7)	34 (48.6)	23 (54.8)	11 (39.3)
Missing	71 (4.8)	30 (3.9)	6 (8.6)	0	6 (21.4)
*HER2* gene copy number at eligibility screening^d^					
<4	21 (1.4)	11 (1.4)	4 (5.7)	3 (7.1)	1 (3.6)
4 to <6	183 (12.3)	81 (10.5)	12 (17.1)	7 (16.7)	5 (17.9)
≥6	1211 (81.5)	653 (84.3)	48 (68.6)	32 (76.2)	16 (57.1)
Missing	71 (4.8)	30 (3.9)	6 (8.6)	0	6 (21.4)
HER2 heterogeneity^e^ at eligibility screening^d^					
Focal (<30%)	166 (11.2)	65 (8.4)	20 (28.6)	12 (28.6)	8 (28.6)
Heterogeneous (30–79%)	325 (21.9)	151 (19.5)	20 (28.6)	11 (26.2)	9 (32.1)
Homogeneous (≥80%)	992 (66.8)	558 (72.0)	30 (42.9)	19 (45.2)	11 (39.3)
Missing	3 (0.2)	1 (0.1)	0	0	0
Median *HER2* gene expression in residual disease (IQR), log2(nCPM)	n/a^f^	(*n* = 534) 10.8 (9.5–12.2)	(*n* = 44) 8.5 (8.0–9.1)	(*n* = 27) 8.3 (7.7–9.0)	(*n* = 17) 8.8 (8.4–9.2)

^a^Data are No. (%) unless otherwise indicated.

^b^Two patients in the IHC2+/ISH+ subgroup were deemed HER2-positive based on the DAKO IQFISH pharmDx test and had an unknown Ventana DDISH test result.

^c^Race data were provided by patient self-report (patient-defined race options).

^d^For the ITT population, these data may have been from specimens obtained prior to neoadjuvant therapy or at surgery.

^e^Tumors were categorized into HER2 IHC2+/3+ heterogeneity categories based on the percentage of cells that stained positive for HER2. If the percentage of cells that stained positive for HER2 was <30%, the tumor was categorized as HER2 focal; if the percentage was 30–79%, the tumor was categorized as HER2 heterogeneous, and if the percentage was ≥80%, the tumor was categorized as HER2 homogeneous. Tumors were analyzed using the sum of complete membrane staining with IHC2+/3+ intensity.

^f^HER2 gene expression assessed for this analysis was based on RNA sequencing analysis which was performed only on evaluable samples obtained at surgery after neoadjuvant therapy.

HER2 gene expression, assessed by RNA sequencing of the surgical samples, was consistent with HER2 status determined by IHC and ISH, showing median HER2 gene expression of 10.8 and 8.5 in HER2-positive and HER2-negative residual disease, respectively (Table 1). While the mechanisms underlying an apparent change in HER2 expression after NAST are unclear, it has been postulated that HER2-negative cells are selected by HER2-directed NAST^8^. Indeed, higher rates of HER2-negative testing results have been demonstrated after NAST containing HER2-targeted therapy plus chemotherapy compared to chemotherapy alone^7^, and, in our study, patients with HER2-negative residual disease were more likely to have received dual versus single HER2-targeted NAST. As trastuzumab + pertuzumab-based therapy becomes a universal standard of care for NAST, it will be interesting to evaluate whether increased selection for HER2-negative cells may occur, with a higher rate of conversion to HER2-negative status in residual disease. Reduced reliability of testing in post-NAST specimens could also contribute to apparent HER2 status conversion.

Next, we assessed the potential impact of change in HER2 status on outcomes. In the overall population, regardless of treatment arm, there was no meaningful difference in IDFS between patients who converted to HER2-negative at surgery and those who remained HER2-positive (HR = 0.93; 95% CI: 0.50–1.71). Among those with HER2-negative residual disease, baseline characteristics were balanced between treatment arms (Table 1). There were 11 IDFS events in the 42 patients (26.2%) randomized to trastuzumab, and no events in the 28 randomized to T-DM1. Of the 11 IDFS events in the trastuzumab arm, seven were distant recurrences not in the central nervous system, one was a central nervous system recurrence, two were locoregional recurrences, and one was contralateral breast cancer.

Previous studies have suggested that HER2-negative status of residual disease after NAST is associated with poor prognosis^6,7,10^, and in this analysis of KATHERINE, patients with HER2-negative residual disease in the trastuzumab arm had a 3-year IDFS of only 70% (Fig. 1b). In contrast, no IDFS events were reported in those receiving T-DM1, suggesting this adverse prognostic effect may be offset with T-DM1. These data are consistent with other data from KATHERINE showing that biomarkers assessed in the surgical sample affected outcomes in the trastuzumab arm, but not in the T-DM1 arm^12^, however they are limited in that paired sample data were not available from all patients because of insufficient tumor material and/or invalid or unknown HER2 status results.

While the analysis of this small subset of patients must be considered exploratory and descriptive, the data do not support a hypothesis that subsets of patients presenting with HER2-positive EBC found to have HER2-negative residual disease on retesting after NAST may not derive benefit from adjuvant T-DM1. These results and the low rate of HER2 status conversion further suggest that HER2 retesting of residual disease has no clinical utility and should not be a prerequisite for T-DM1 therapy in this setting.

## Methods

The KATHERINE study (NCT01772472, registered January 21, 2013) evaluated T-DM1 in patients with HER2-positive EBC who had residual invasive disease and had received taxane/trastuzumab-based NAST. The study design, including patient eligibility criteria and patient disposition, have been published previously^3^. In brief, patients were eligible for the study if they had histologically confirmed, centrally confirmed, HER2-positive, non-metastatic, invasive primary breast cancer (T1–4, N0–3, M0 [excluding T1aN0 and T1bN0]) at presentation and residual invasive disease detected pathologically in the surgical specimen of the breast or axillary lymph nodes after completion of taxane-based neoadjuvant chemotherapy administered with trastuzumab. Patients were required to have completed at least six cycles (≥16 weeks) of NAST including ≥9 weeks of trastuzumab and ≥9 weeks of taxane-based chemotherapy (or, if receiving dose-dense chemotherapy regimens, ≥6 to 8 weeks of taxane-based therapy and ≥8 weeks of trastuzumab). HER2-directed therapy and chemotherapy could be given concurrently, and patients could have received more than one HER2-directed therapy, and anthracyclines and alkylating agents as part of preoperative therapy. Within 12 weeks of surgery, patients were randomized to adjuvant trastuzumab (6 mg/kg intravenously every 3 weeks) or T‐DM1 (3.6 mg/kg intravenously every 3 weeks) for 14 cycles. Adjuvant radiotherapy and adjuvant hormonal therapy were permitted, as indicated. The KATHERINE study methods were performed in accordance with relevant guidelines and regulations and approved by the institutional review board at each participating center (e.g., Houston Methodist Institutional Review Board). KATHERINE was conducted in accordance with the International Council for Harmonisation E6 Good Clinical Practice Guideline and the principles of the Declaration of Helsinki; and followed local laws and regulations. All patients provided written informed consent.

HER2 status for study eligibility was performed preferentially on specimens collected pre-NAST and, in the analysis described herein, was also assessed on paired residual samples submitted for correlative studies. Samples were submitted to the central laboratory in the form of a formalin-fixed paraffin-embedded tumor block or partial block or slides obtained from the pretreatment primary tumor biopsy material (or residual tumor tissue from definitive surgery post-NAST). HER2 status of all samples was centrally assessed in the same laboratory (Targos Molecular Pathology, GmbH [Kassel, Germany]) and according to interpretation guidelines of the Ventana assays (PATHWAY^®^ anti-HER-2/neu [4B5] assay with rabbit monoclonal primary antibody, Ventana Medical Systems, Inc., cat#790–2991 and INFORM HER2 Dual ISH assay, DNA Probe Cocktail, Ventana Medical Systems, Inc., cat#800–4422). Breast cancer was considered HER2-positive with an immunohistochemistry (IHC) score of 3+ or amplification of HER2 by in situ hybridization (ISH), defined as a ratio of ≥2.0 for the number of HER2 gene copies to chromosome 17 copies. HER2 expression by IHC was recorded as focal (<30%), heterogeneous (30–79%), or homogeneous (≥80%), based on the percentage of cells stained with IHC2+/3+ intensity. RNA expression of the whole transcriptome was measured using RNA sequencing (RNA-seq) with TruSeq RNA Access (Illumina, Inc., San Diego, California) at Expression Analysis (Morrisville, North Carolina) on macro-dissected tumor samples. Results from RNA analysis, adjusted for tumor content, were used to quantify HER2 (*ERBB2*) expression in support of the IHC and ISH analyses. Gene expression normalization and transformation to log2(nCPM) was performed with the edgeR R package (version 3.32.1). To regress out the tumor content effect the limma R package (version 3.42.0) was used. Median and IQRs of HER2 gene expression were estimated with the quantile function from the stats R base package (R version 4.0.5).

The endpoint for this exploratory analysis was IDFS, defined as the time from randomization until first occurrence of: recurrence of ipsilateral invasive breast cancer, recurrence of ipsilateral locoregional or contralateral invasive breast cancer, distant recurrence, or death from any cause. Unstratified hazard ratios and 95% confidence intervals were estimated using Cox proportional hazards models. Three-year IDFS rates were estimated with the Kaplan–Meier method. *P* values were not computed since analyses were exploratory. IDFS was evaluated by randomized treatment arm in patients who had paired specimens with central HER2 status results.

### Reporting summary

Further information on research design is available in the Nature Research Reporting Summary linked to this article.

## Supplementary information

Supplementary Table 1

Protocol

## Data Availability

Qualified researchers may request access to individual patient level clinical data through a data request platform. At the time of this writing this request platform is Vivli: https://vivli.org/ourmember/roche/ For up to date details on Roche’s Global Policy on the Sharing of Clinical Information and how to request access to related clinical study documents, see here: https://www.roche.com/innovation/process/clinical-trials/data-sharing/ Individual patient level HER2 gene expression data and limited clinical data including treatment arm and central HER2 status pre-NAT and at surgery are available to qualified researchers at The European Genome-phenome Archive (https://ega-archive.org/access/data-access) under accession number (EGAS00001006037). Anonymised records for individual patients across more than one data source external to Roche cannot, and should not, be linked due to a potential increase in risk of patient re-identification.

## Code availability

There were no special new codes generated for any analysis in this paper.
