# Supplementary material for: Adjuvant trastuzumab emtansine in HER2-positive breast cancer patients with HER2-negative residual invasive disease in KATHERINE
Source: NPJ Breast Cancer. 2022 Sep 19;8:106. doi: 10.1038/s41523-022-00477-z (PMC9482917; doi:10.1038/s41523-022-00477-z)
Supplement: Supplementary file 1 — Supplementary Table 1 [file 41523_2022_477_MOESM1_ESM.pdf]

Supplementary Table 1. Clinical stage by HER2 status at surgery in patients with paired pre-neoadjuvant and surgical samples

Stage	Clinical stage at initial diagnosis		Stage	Pathological stage at surgery	
	**HER2-positive at surgery (*n* = 775)**	**HER2-negative at surgery (*n* = 70)**		**HER2-positive at surgery (*n* = 775)**	**HER2-negative at surgery (*n* = 70)**
I	4.5% (*n* = 35)	8.6% (*n* = 6)	I	38.8% (*n* = 301)	47.1% (*n* = 33)
II	57.5% (*n* = 446)	47.1% (*n* = 33)	II	38.7% (*n* = 300)	31.4% (*n* = 22)
III	37.9% (*n* = 294)	44.3% (*n* = 31)	III	22.5% (*n* = 174)	21.4% (*n* = 15)

Abbreviation: HER2, human epidermal growth factor receptor 2.
