# Supplementary material for: Adjuvant trastuzumab emtansine in HER2-positive breast cancer patients with HER2-negative residual invasive disease in KATHERINE
Source: NPJ Breast Cancer. 2022 Sep 19;8:106. doi: 10.1038/s41523-022-00477-z (PMC9482917; doi:10.1038/s41523-022-00477-z)
Supplement: Supplementary file 2 — Protocol [file 41523_2022_477_MOESM2_ESM.pdf]

PROTOCOL

TITLE: **A RANDOMIZED, MULTICENTER, OPEN-LABEL PHASE III STUDY TO EVALUATE THE EFFICACY AND SAFETY OF TRASTUZUMAB EMTANSINE VERSUS TRASTUZUMAB AS ADJUVANT THERAPY FOR PATIENTS WITH HER2-POSITIVE PRIMARY BREAST CANCER WHO HAVE RESIDUAL TUMOR PRESENT PATHOLOGICALLY IN THE BREAST OR AXILLARY LYMPH NODES FOLLOWING PREOPERATIVE THERAPY**

PROTOCOL NUMBER: BO27938

VERSION NUMBER: 1

EUDRACT NUMBER: 2012-002018-37

IND NUMBER: 71,072

TEST PRODUCT: Trastuzumab emtansine

MEDICAL MONITOR: [redacted]

SPONSOR: F. Hoffmann-La Roche Ltd

DATE FINAL: See electronic date stamp below

FINAL PROTOCOL APPROVAL

Approver's Name	Title	Date and Time (UTC)
[redacted]	Company Signatory	28-Jun-2012 14:53:57

CONFIDENTIAL STATEMENT

The information contained in this document, especially any unpublished data, is the property of F. Hoffmann-La Roche Ltd (or under its control) and therefore is provided to you in confidence as an investigator, potential investigator, or consultant, for review by you, your staff, and an applicable Ethics Committee or Institutional Review Board. It is understood that this information will not be disclosed to others without written authorization from Roche except to the extent necessary to obtain informed consent from persons to whom the drug may be administered.

TABLE OF CONTENTS

PROTOCOL ACCEPTANCE FORM 9

PROTOCOL SYNOPSIS 10

1. BACKGROUND 21
   1.1 Background on Early-Stage HER2-Positive Breast Cancer 21
   1.2 Background on Trastuzumab Emtansine 23
      1.2.1 Study TDM4450g/BO21976 23
      1.2.2 Study TDM4874g/BO22857 24
      1.2.3 Study TDM4370g/BO21977 (EMILIA) 25
      1.2.4 Study TDM4788g/BO22589 (MARIANNE) 26
      1.2.5 Studies TDM4258g and TDM4374g 26
   1.3 Background on Trastuzumab (Herceptin®) 26
   1.4 Study Rationale and Benefit Risk Assessment 26

2. OBJECTIVES 27
   2.1 Primary Efficacy Objective 27
   2.2 Safety Objectives 27
   2.3 Patient Reported Outcome Objectives 27
   2.4 Pharmacokinetics Objectives 28
   2.5 Exploratory Objectives 28

3. STUDY DESIGN 28
   3.1 Description of Study 28
      3.1.1 Overview 28
      3.1.2 Data Monitoring Committee 30
      3.1.3 Clinical Events Committee 30
   3.2 End of Study 30
   3.3 Rationale for Study Design 30
      3.3.1 Rationale for Test Product Dosage 30
      3.3.2 Rationale for Patient Population 31
      3.3.3 Rationale for Control Group and Duration of Therapy 31
      3.3.4 Rationale for Biomarker Assessments 31

3.3.5 Rationale for Pharmacokinetic Assessments 32
3.4 OUTCOME MEASURES 32
3.4.1 Primary Efficacy Outcome Measure 32
3.4.2 Secondary Efficacy Outcome Measures 32
3.4.3 Safety Outcome Measures 33
3.4.4 Patient-Reported Outcome Measures 33
3.4.5 Pharmacokinetic Outcome Measures 33
3.4.6 Exploratory Outcome Measure 34

4. MATERIALS AND METHODS 34
4.1 PATIENTS 34
4.1.1 Inclusion Criteria 34
4.1.1.1 Disease-Specific Inclusion Criteria 34
4.1.1.2 General Inclusion Criteria 35
4.1.2 Exclusion Criteria 37
4.1.2.1 Disease-Related Exclusion Criteria 37
4.1.2.2 General Exclusion Criteria 38
4.2 Method of Treatment Assignment and Blinding 38
4.3 Study Treatment 38
4.3.1 Formulation, Packaging, and Handling 38
4.3.1.1 Trastuzumab Emtansine and Trastuzumab 39
4.3.1.1.1 Trastuzumab Emtansine 39
4.3.1.1.2 Trastuzumab 39
4.3.2 Dosage, Administration, and Compliance 39
4.3.2.1 Trastuzumab Emtansine 39
4.3.2.2 Trastuzumab 40
4.3.3 Investigational Medicinal Product Accountability 40
4.3.3.1 Post-Trial Access to Trastuzumab Emtansine 41
4.4 Concomitant Therapy and Food 41
4.4.1 Permitted Therapy 41
4.4.1.1 Adjuvant Radiotherapy 42
4.4.1.2 Concomitant Hormonal Therapy 42
4.4.2 Prohibited Therapy 43
4.5 Study Assessments 43

4.5.1 Description of Study Assessments 44
4.5.1.1 Medical History and Demographic Data 44
4.5.1.2 Vital Signs 44
4.5.1.3 Physical Examinations 44
4.5.1.4 Radiologic Evaluations 44
4.5.1.5 Laboratory Assessments 44
4.5.1.6 Cardiac Assessments... 46
4.5.1.6.1 Electrocardiograms 46
4.5.1.6.2 Left Ventricular Ejection Fraction 46
4.5.1.7 Patient-Reported Outcomes 46
4.5.1.8 Mandatory Samples for Determination of Patient Eligibility and Exploratory Biomarker Research 47
4.5.1.9 Optional Biomarker Research Samples 48
4.5.2 Timing of Study Assessments 52
4.5.2.1 Screening and Pretreatment Assessments 52
4.5.2.2 Assessments during Treatment... 52
4.5.2.3 Assessments at Study Treatment Completion/Early Termination Visit..................................... 53
4.5.2.4 Follow-Up Assessments 53
4.5.2.4.1 Scheduled Follow-up Assessments 53
4.5.2.4.2 Follow-up and Confirmation of Disease Recurrence 54
4.6 Patient, Study, and Site Discontinuation 57
4.6.1 Patient Discontinuation 57
4.6.1.1 Discontinuation from Study Drug... 57
4.6.1.2 Withdrawal from Study 58
4.6.2 Study and Site Discontinuation 59
5. ASSESSMENT OF SAFETY 59
5.1 Safety Plan 59
5.1.1 Toxicities Associated with Trastuzumab Emtansine 59
5.1.2 Toxicities Associated with Trastuzumab 60
5.1.3 Management of Specific Adverse Events 60
5.2 Safety Parameters and Definitions 65

5.2.1 Adverse Events ... 65
5.2.2 Serious Adverse Events (Immediately Reportable to the Sponsor) ... 65
5.2.3 Non-Serious Adverse Events of Special Interest (Immediately Reportable to the Sponsor) ... 66
5.3 Methods and Timing for Capturing and Assessing Safety Parameters ... 66
5.3.1 Adverse Event Reporting Period ... 66
5.3.2 Eliciting Adverse Event Information ... 67
5.3.3 Assessment of Severity of Adverse Events ... 67
5.3.4 Assessment of Causality of Adverse Events ... 68
5.3.5 Procedures for Recording Adverse Events ... 68
5.3.5.1 Diagnosis versus Signs and Symptoms ... 68
5.3.5.2 AEs Occurring Secondary to Other Events ... 69
5.3.5.3 Persistent or Recurrent Adverse Events ... 69
5.3.5.4 Abnormal Laboratory Values ... 69
5.3.5.5 Abnormal Vital Sign Values ... 70
5.3.5.6 Hepatotoxicity ... 71
5.3.5.7 Abnormal Left Ventricular Ejection Fraction ... 71
5.3.5.8 Deaths ... 72
5.3.5.9 Preexisting Medical Conditions ... 72
5.3.5.10 Lack of Efficacy or Worsening of Breast Cancer ... 73
5.3.5.11 Hospitalization or Prolonged Hospitalization ... 73
5.3.5.12 Overdoses ... 73
5.3.5.13 Patient-Reported Outcome Data ... 73
5.4 Immediate Reporting Requirements from Investigator to Sponsor ... 74
5.4.1 Emergency Medical Contacts ... 74
5.4.2 Reporting Requirements for Serious Adverse Events and Non-Serious Adverse Events of Special Interest ... 74
5.4.3 Reporting Requirements for Pregnancies ... 75
5.4.3.1 Pregnancies in Female Patients ... 75

5.4.3.2 Pregnancies in Female Partners of Male Patients 75
5.4.3.3 Abortions 76
5.4.3.4 Congenital Anomalies/Birth Defects 76
5.5 Follow-Up of Patients after Adverse Events 76
5.5.1 Investigator Follow-Up 76
5.5.2 Sponsor Follow-Up 76
5.6 Post-Study Adverse Events 76
5.7 Expedited Reporting to Health Authorities, Investigators, Institutional Review Boards, and Ethics Committees 77
6. STATISTICAL CONSIDERATIONS AND ANALYSIS PLAN 77
6.1 Determination of Sample Size 77
6.2 Summaries of Conduct of Study 78
6.3 Summaries of Treatment Group Comparability 78
6.4 Efficacy Analyses 78
6.4.1 Primary Efficacy Endpoint 78
6.4.2 Secondary Efficacy Endpoints 78
6.5 Safety Analyses 79
6.6 Patient-Reported Outcome Analyses 80
6.7 Pharmacokinetic analyses 80
6.8 Exploratory Analyses 81
6.9 Interim Analyses 81
6.9.1 Interim Efficacy Analyses 81
6.9.2 Interim Safety Analyses 82
7. DATA COLLECTION AND MANAGEMENT 84
7.1 Data Quality Assurance 84
7.2 Electronic Case Report Forms 85
7.3 Electronic Patient-Reported Outcome Data 85
7.4 Source Data Documentation 85
7.5 Use of Computerized Systems 86
7.6 Retention of Records 86
8. ETHICAL CONSIDERATIONS 86

8.1 Compliance with Laws and Regulations ... 86
8.2 Informed Consent ... 87
8.3 Institutional Review Board or Ethics Committee ... 88
8.4 Confidentiality ... 88
8.5 Financial Disclosure ... 89

9. STUDY DOCUMENTATION, MONITORING, AND ADMINISTRATION ... 89
9.1 Study Documentation ... 89
9.2 Site Inspections ... 89
9.3 Administrative Structure ... 89
9.4 Publication of Data and Protection of Trade Secrets ... 89
9.5 Protocol Amendments ... 90

10. REFERENCES ... 91

LIST OF TABLES

Table 1 Recommendations for Hormonal Therapy ... 43
Table 2 Dose Reduction for Trastuzumab Emtansine ... 60
Table 3 Guidelines for Managing Specific Adverse Events ... 61
Table 4 Adverse Event Severity Grading Scale ... 67
Table 5 Summary of Planned Analyses of Invasive Disease-Free Survival ... 81
Table 6 Summary of Planned Analyses of Overall Survival ... 82
Table 7 Probability of Observing $>$ 3% Increase of Death ... 83
Table 8 Probability of Observing $>$ 3% Increase of Confirmed Hy's Law Cases ... 84

LIST OF FIGURES

Figure 1 Tissue Collection Flow ... 48
Figure 2 Algorithm for Continuation and Discontinuation of Study Treatment Based on LVEF Assessment ... 64

LIST OF APPENDICES

Appendix 1 Schedule of Assessments 93
Appendix 2 Schedule of Pharmacodynamic and Pharmacokinetic Assessments 97
Appendix 3 European Organisation for Research and Treatment of Cancer Quality of Life Questionnaire – Core 30 99
Appendix 4 European Organisation for Research and Treatment of Cancer Quality of Life Questionnaire – Breast Cancer 23 101
Appendix 5 EuroQoL EQ-5D 103

PROTOCOL ACCEPTANCE FORM

TITLE: **A RANDOMIZED, MULTICENTER, OPEN-LABEL PHASE III STUDY TO EVALUATE THE EFFICACY AND SAFETY OF TRASTUZUMAB EMTANSINE VERSUS TRASTUZUMAB AS ADJUVANT THERAPY FOR PATIENTS WITH HER2-POSITIVE PRIMARY BREAST CANCER WHO HAVE RESIDUAL TUMOR PRESENT PATHOLOGICALLY IN THE BREAST OR AXILLARY LYMPH NODES FOLLOWING PREOPERATIVE THERAPY**

**PROTOCOL NUMBER:**	BO27938
**VERSION NUMBER:**	1
**EUDRACT NUMBER:**	2012-002018-37
**IND NUMBER:**	71,072
**TEST PRODUCT:**	BO27938
**MEDICAL MONITOR:**	[redacted]
**SPONSOR:**	F. Hoffmann-La Roche Ltd

I agree to conduct the study in accordance with the current protocol.

____

Principal Investigator's Name (print)

____ ________________

Principal Investigator's Signature Date

Please return the original signed form to your local study monitor. Please retain a copy of the signed form for your study files.

PROTOCOL SYNOPSIS

TITLE: A RANDOMIZED, MULTICENTER, OPEN-LABEL PHASE III STUDY TO EVALUATE THE EFFICACY AND SAFETY OF TRASTUZUMAB EMTANSINE VERSUS TRASTUZUMAB AS ADJUVANT THERAPY FOR PATIENTS WITH HER2-POSITIVE PRIMARY BREAST CANCER WHO HAVE RESIDUAL TUMOR PRESENT PATHOLOGICALLY IN THE BREAST OR AXILLARY LYMPH NODES FOLLOWING PREOPERATIVE THERAPY

**PROTOCOL NUMBER:**	BO27938
**VERSION NUMBER:**	1
**Eudract Number:**	2012-002018-37
**IND NUMBER:**	71,072
**TEST PRODUCT:**	Trastuzumab emtansine
**PHASE:**	III
**INDICATION:**	HER2-positive primary breast cancer
**SPONSOR:**	F. Hoffmann-La Roche Ltd

Objectives

Primary Efficacy Objective

The primary efficacy objective for this study is as follows:

- To compare invasive disease-free survival (IDFS) in patients with residual invasive breast cancer after treatment with preoperative chemotherapy and HER2-directed therapy including trastuzumab followed by surgery between the 2 treatment arms

The secondary efficacy objective for this study is as follows:

- To compare IDFS including second non-breast cancers, disease-free survival (DFS), overall survival (OS), and distant recurrence-free interval (DRFI) between the 2 treatment arms

Safety Objectives

The safety objective for this study is as follows:

- To compare cardiac safety and overall safety between the 2 treatment arms according to the National Cancer Institute Common Terminology Criteria for Adverse Events (NCI CTCAE), Version 4.0

Patient Reported Outcome Objectives

The patient-reported outcome (PRO) objective for this study is as follows:

- To compare PROs between the 2 treatment arms using the European Organisation for Research and Treatment of Cancer (EORTC) Quality of Life Questionnaire – Core 30 (QLQ-C30) questionnaire and Quality of Life Questionnaire – Breast Cancer 13 (QLQ-BR23) module

Pharmacokinetics Objectives

The pharmacokinetics (PK) objectives for this study are as follows:

- To characterize the PK of trastuzumab emtansine (including total trastuzumab and DM1) in trastuzumab emtansine treated patients

- To characterize the PK of trastuzumab in trastuzumab-treated patients and permit an intra-study comparison of trastuzumab exposure in the 2 treatment arms
- To investigate exposure–effect (efficacy and safety) relationships in this patient population

Exploratory Objectives

The exploratory objectives for this study are as follows:

- To assess correlations between biomarker status and efficacy and/or safety
- To assess the incidence of anti-therapeutic antibodies (ATAs) and the effect of ATAs on PK, safety, and efficacy

Study Design

Description of Study

This is a Phase III, 2-arm, randomized, multicenter, multinational, open-label study in patients with HER2-positive primary breast cancer who have received preoperative chemotherapy and HER2-directed therapy including trastuzumab followed by surgery, with a finding of residual invasive disease in the breast or axillary lymph nodes.

Patients who provide consent will commence a screening period, which will last at least 30 days. Informed consent forms may be obtained at any time (including prior to the 30-day screening period) but must be obtained prior to the performance of any screening assessments. Patients who have pathologically documented residual invasive disease in either the breast or axillary lymph nodes following completion of preoperative therapy (including, but not limited to, at least 9 weeks of HER2-directed therapy including trastuzumab, at least 9 weeks of taxane therapy, and at least 16 weeks of total systemic treatment in the preoperative setting) will be eligible to participate in the study.

At the end of the screening period, eligible patients will be randomized in a 1:1 ratio to receive open-label study treatment (trastuzumab emtansine 3.6 mg/kg every 3 weeks [q3w] for 14 cycles or trastuzumab 6 mg/kg q3w for 14 cycles). Randomization will be stratified by clinical stage at presentation (inoperable [Stage T4NxM0 or TxN2–3M0], operable [stages T1-3N0 to 1M0]), hormone receptor status (estrogen receptor [ER] or progesterone receptor [PR] positive, ER and PR negative), preoperative HER2-directed therapy (trastuzumab, trastuzumab plus additional HER2-directed agent[s]), and pathological nodal status evaluated after preoperative therapy (node positive, node negative or not done). Patients will be administered radiotherapy and/or hormonal therapy (for patients with hormone receptor-positive tumors) in addition to receiving study treatment for 14 weeks if indicated based on the following guidelines:

- Hormonal therapy (aromatase inhibitor, tamoxifen, etc.) should be initiated in patients with hormone receptor-positive disease at presentation.
- For patients undergoing breast-conserving surgery, whole breast irradiation is required. Regional node irradiation is required if the patient presented at initial diagnosis with clinical T3 or T4 disease and/or with clinical N2 or N3 disease; it is recommended if there is residual disease in lymph nodes.
- For post-mastectomy patients, chest wall and regional node irradiation is required if the patient presented at initial diagnosis with clinical T3 or T4 disease and/or with clinical N2 or N3 disease; it is recommended if there is residual disease in lymph nodes. For post-mastectomy patients who do not meet these criteria, radiotherapy is at the discretion of the investigator based on institutional standards.

The first dose of study treatment will be administered on Day 1 of a 3-week cycle, i.e., dosing will be repeated once q3w to complete a maximum of 14 cycles of treatment. Treatment will be discontinued prior to 14 cycles in the event of disease recurrence, unacceptable toxicity, or study termination by the Sponsor. Efficacy, safety, laboratory, and PRO measures will be assessed throughout the study, as detailed in the schedule of assessments (see Appendix 1). PK measures will be assessed as specified in the schedule of pharmacodynamic and PK assessments (see Appendix 2). The primary efficacy endpoint is the IDFS and will be measured from the time of randomization until its first occurrence. Following discontinuation or completion of study treatment, all patients will continue to be followed for efficacy and safety objectives until the end of the study.

Number of Patients

A planned total of 1484 patients will be enrolled in the study.

Target Population

Patients must meet the following criteria for study entry:

1. HER2-positive breast cancer

    Positivity will be based on pretreatment biopsy and defined as immunohistochemistry (IHC) score of 3+and/or positive by in situ hybridization (ISH) prospectively confirmed by a central laboratory prior to study enrollment. Paraffin-embedded tumor tissue block or a partial block must be obtained. If sites are unable to send a tissue block due to local regulations, at least 8 unstained slides should be sent for HER2 testing, and in addition up to 5 slides for exploratory biomarker research. ISH positivity is defined as a ratio of ≥ 2.0 for the number of HER2 gene copies to the number of signals for CEP17. Both IHC and ISH assays will be performed; however, only one positive result is required for eligibility. In the event that sufficient material from the pretreatment biopsy is not available for submission, central HER2 determination for eligibility may be performed on residual tumor tissue from the time of definitive surgery.

    Patients with synchronous bilateral invasive disease are eligible provided both lesions are HER2-positive.

2. Histologically confirmed invasive breast carcinoma
3. Clinical stage at presentation: T1–4, N0–3, M0 (Note: Patients with T1a/bN0 tumors will not be eligible)
4. Completion of preoperative systemic treatment consisting of at least 6 cycles with a total duration at least 16 weeks, including at least 9 weeks of trastuzumab and at least 9 weeks of taxane-based chemotherapy.

    Note: HER2-directed therapy and chemotherapy may be given concurrently; patients may have received more than one HER2-directed therapy. Patients may have received an anthracycline as part of preoperative therapy.

5. Adequate excision: surgical removal of all clinically evident disease in the breast and lymph nodes as follows:

    Breast surgery: total mastectomy with no gross residual disease at the margin of resection, or breast-conserving surgery with histologically negative margins of excision

        For patients who undergo breast-conserving surgery, the margins of the resected specimen must be histologically free of invasive tumor and ductal carcinoma in situ (DCIS) as determined by the local pathologist. If pathologic examination demonstrates tumor at the line of resection, additional operative procedures may be performed to obtain clear margins. If tumor is still present at the resected margin after re-excision(s), the patient must undergo total mastectomy to be eligible. Patients with margins positive for lobular carcinoma in situ (LCIS) are eligible without additional resection.

    Lymph node surgery:

        In case of positive results from a fine-needle aspiration, core biopsy, or sentinel node biopsy performed prior to preoperative therapy, additional surgical evaluation of the axilla following preoperative therapy is required.

        If sentinel node biopsy performed before preoperative therapy was negative, no additional surgery evaluation of the axilla is required after preoperative therapy.

        If the only sentinel node identified by isotope scan is in the internal mammary chain, surgical evaluation of the axilla is recommended.

        If sentinel node biopsy performed after preoperative therapy is positive, additional surgical evaluation of the axilla is recommended.

        If sentinel node evaluation after preoperativetherapy is negative, no further additional surgical evaluation of the axilla is required.

        Axillary dissection without sentinel node evaluation is permitted after preoperative therapy.

6. Pathologic evidence of residual invasive carcinoma in the breast or axillary lymph nodes following completion of preoperative therapy
7. An interval of no more than 12 weeks between the date of surgery and the date of randomization
8. Known hormone receptor status (either ER and/or PR of the primary tumor)
9. Signed written informed consent approved by the study site's Institutional Review Board (IRB)/Ethical Committee (EC)
10. Age $\geq$ 18 years
11. Eastern Cooperative Oncology Group (ECOG) performance status 0 or 1
12. Life expectancy $\geq$ 6 months
13. Adequate organ function during screening, defined as:

    Absolute neutrophil count $\geq$ 1200 cells/mm^3

    Platelet count $\geq$ 100000 cells/mm^3

    Hemoglobin $\geq$ 9.0 g/dL; patients may receive red blood cell transfusions to obtain this level

    Serum creatinine $\geq$ 1.5 $\times$ upper limit of normal (ULN)

    International normalized ratio (INR) and activated partial thromboplastin time (aPTT) $\leq$ 1.5 $\times$ ULN

    Serum aspartate aminotransferase (AST) and alanine aminotransferase (ALT) $\leq$ 1.5 $\times$ ULN

    Serum total bilirubin (TBILI) $\leq$ 1.0 $\times$ ULN (within normal limits), except for patients with Gilbert's syndrome, for whom direct bilirubin should be within the normal range

    Serum alkaline phosphatase (ALK) $\leq$ 1.5 $\times$ ULN

    Screening left ventricular ejection fraction (LVEF) $\geq$ 50% on echocardiogram (ECHO) or multiple-gated acquisition (MUGA) and no decrease in LVEF by more than 15% absolute points from the pre-chemotherapy LVEF (pre-chemotherapy LVEF must be documented in the CRF)

    LVEF assessment may be repeated once up to 3 weeks following the initial screening assessment to assess eligibility
14. Women of childbearing potential and men with partners of childbearing potential must be willing to use one highly effective form of nonhormonal contraception or 2 effective forms of nonhormonal contraception by the patient and/or partner and to continue its use for the duration of study treatment and for 6 months after the last dose of study treatment
    a) Acceptable forms of effective contraception should include 2 of the following:
        i. Placement of non-hormonal intrauterine device (IUD)
        ii. Condom with spermicidal foam/gel/film/cream/suppository
        iii. Diaphragm or cervical/vault caps with spermicidal foam/film/cream/suppository
    b) The above contraception is not a requirement in the case of any of the following:
        i. The male patient, or male partner of a female patient, is surgically sterilized.
        ii. The female patient is $>$ 45 years of age and is postmenopausal (has not menstruated for at least 12 consecutive months)
        iii. The patient truly abstains from sexual activity and when this is the preferred and usual lifestyle of the patient.
    c) Contraception use should continue for the duration of the study treatment and for at least 6 months after the last dose of study treatment. Periodic abstinence (e.g., calendar ovulation, symptothermal, and post-ovulation methods) and withdrawal are not acceptable methods of contraception.
15. Negative serum pregnancy test for premenopausal women including women who have had a tubal ligation and for women less than 12 months after the onset of menopause

Patients who meet any of the following criteria will be excluded from study entry:

1. Stage IV (metastatic) breast cancer
2. History of any prior (ipsi- or contralateral) breast cancer except LCIS
3. Evidence of clinically evident gross residual or recurrent disease following preoperative therapy and surgery
4. Progressive disease (PD) during preoperative therapy
5. Treatment with any anti-cancer investigational drug within 28 days prior to commencing study treatment
6. History of other malignancy within the last 5 years except for appropriately treated carcinoma in situ (CIS) of the cervix, non-melanoma skin carcinoma, Stage I uterine cancer, or other non-breast malignancies with an outcome similar to those mentioned above
7. Patients for whom radiotherapy would be recommended for breast cancer treatment but for whom it is contraindicated because of medical reasons (e.g., connective tissue disorder or prior ipsilateral breast radiation)
8. Current NCI CTCAE (Version 4.0) Grade $\geq$ 2 peripheral neuropathy
9. History of exposure to the following cumulative doses of anthracyclines:
    - Doxorubicin $>$ 240 mg/m^2
    - Epirubicin $>$ 480 mg/m^2
    - For other anthracyclines, exposure equivalent to doxorubicin $>$ 240 mg/m^2
10. Cardiopulmonary dysfunction as defined by any of the following:
    - Significant symptoms (Grade $\geq$ 2) relating to LV dysfunction, cardiac arrhythmia, or cardiac ischemia while or since receiving preoperative therapy.
    - Uncontrolled hypertension (systolic blood pressure $>$ 180 mmHg and/or diastolic blood pressure $>$ 100 mmHg)
    - Inadequately controlled angina, serious cardiac arrhythmia not controlled by adequate medication, severe conduction abnormality, or clinically significant valvular disease
    - Screening LVEF $<$ 50% by either ECHO or MUGA
    - History of NCI CTCAE (Version 4.0) Grade $\geq$ 3 symptomatic congestive heart failure (CHF) or New York Heart Association (NYHA) criteria Class ≥ II
    - History of a decrease in LVEF to $<$ 40% or symptomatic CHF with prior trastuzumab treatment (e.g., during preoperative therapy)
    - Myocardial infarction within 12 months prior to randomization
    - Requirement for continuous oxygen therapy
11. Prior treatment with trastuzumab emtansine
12. Current severe, uncontrolled systemic disease (e.g., clinically significant cardiovascular, pulmonary, or metabolic disease; wound healing disorders; ulcers)
13. For female patients, current pregnancy and/or lactation
14. Major surgical procedure unrelated to breast cancer or significant traumatic injury within approximately 28 days prior to randomization or anticipation of the need for major surgery during the course of study treatment
15. Any known liver disease, including known carriers of hepatitis B virus, hepatitis C, autoimmune hepatic disorders and sclerosing cholangitis
16. Concurrent, serious, uncontrolled infections or known infection with HIV
17. History of intolerance, including Grade 3 to 4 infusion reaction or hypersensitivity to trastuzumab or murine proteins
18. Active, unresolved infections at screening
19. Assessment by the investigator as being unable or unwilling to comply with the requirements of the protocol

Length of Study

The total length of this study will be approximately 10 years from randomization of the first patient to completion of the last follow-up assessment of the last patient.

End of Study

The study will end after the last patient randomized into the study has undergone the last follow-up assessment. To enable long-term follow-up for survival and safety information, the last follow-up assessment is scheduled to occur 10 years after the first patient is randomized.

Primary Efficacy Outcome Measure

The primary efficacy outcome measure is IDFS, defined as the time from randomization until the date of the first occurrence of any one of the following events:

- Ipsilateral invasive breast tumor recurrence (i.e., an invasive breast cancer involving the same breast parenchyma as the original primary lesion)
- Ipsilateral local-regional invasive breast cancer recurrence (i.e., an invasive breast cancer in the axilla, regional lymph nodes, chest wall and/or skin of the ipsilateral breast)
- Distant recurrence (i.e., evidence of breast cancer in any anatomic site—other than the 2 above-mentioned sites—that has either been histologically confirmed or clinically diagnosed as recurrent invasive breast cancer)
- Contralateral invasive breast cancer
- Death attributable to any cause including breast cancer, non-breast cancer or unknown cause (but cause of death should be specified if at all possible)

Secondary Efficacy Outcome Measures

Secondary efficacy outcome measures include the following:

- IDFS including second primary non-breast cancer: defined the same way as IDFS for the primary endpoint but including second primary non-breast invasive cancer as an event (with the exception of non-melanoma skin cancers and CIS of any site)
- DFS: defined as the time between randomization and the date of the first occurrence of an IDFS event including second primary non-breast cancer event or contralateral or ipsilateral DCIS
- OS: defined as the time from randomization to death due to any cause
- DRFI: defined as the time between randomization and the date of distant breast cancer recurrence

Safety Outcome Measures

The safety outcome measures are the following protocol-specific adverse events (AEs):

- Incidence, type and severity of all AEs based on NCI CTCAE Version 4.0
- Incidence, type, and severity of serious adverse events (SAEs)
- Incidence and type of AEs leading to dose discontinuation, modification, or delay
- Cause of death on study
- Abnormal laboratory values
- LVEF decreases
- Cardiac events, defined as death from cardiac cause or severe CHF (NYHA Class III or IV) with a decrease in LVEF of ≥ 10 percentage points from baseline to an LVEF of $< 50\%$.

Patient-Reported Outcome Measures

The PRO outcome measures for this study are as follows:

- Incidence of treatment-related symptoms and assessment of health-related quality of life (HRQOL) as measured using the EORTC QLQ-C30 questionnaire and QLQ-BR23 module
- Assessment of health status as measured using the EuroQol EQ-5D™ questionnaire for health economic modeling

Pharmacokinetic Outcome Measures

The PK outcome measures to be assessed in patients receiving trastuzumab emtansine are the following:

- Observed serum concentrations and relevant PK parameters of trastuzumab emtansine (trastuzumab emtansine conjugated) and total trastuzumab (sum of conjugated and unconjugated trastuzumab)
- Observed plasma concentrations of DM1
- Explore relationship between trastuzumab emtansine exposure and efficacy/safety
- Characterize ATA and assess impact of ATA on PK, safety and efficacy.

Exploratory Outcome Measures

The exploratory outcome measure for this study is as follows:

- The relationship between molecular markers and efficacy outcomes

Efficacy outcomes considered for this analysis will include IDFS and OS, as appropriate.

Investigational Medicinal Products

Trastuzumab emtansine is provided as a single-use lyophilized formulation in a colorless 20-mL Type I glass vial closed by means of a FluroTec-coated stopper and an overseal with flip-off cap. The lyophilized product should be reconstituted using Sterile Water for Injection (SWFI). The resulting product contains 20 mg/mL trastuzumab emtansine, 10 mM sodium succinate, pH 5.0, 60 mg/mL sucrose, and 0.02% (w/v) polysorbate 20. Each 20 mL vial contains enough trastuzumab emtansine to allow delivery of 160 mg trastuzumab emtansine. Patients will receive trastuzumab emtansine infusions q3w. Vials should be refrigerated at 2°C to 8°C (36°F to 46°F) until use. The vial and the solution of trastuzumab emtansine should not be shaken or frozen.

Information on the formulation, packaging, handling, and administration of trastuzumab is provided in the trastuzumab Investigator's Brochure (IB).

Accurate records of all investigational medicinal products (IMPs), including trastuzumab emtansine and trastuzumab, that are received at, dispensed from, returned to, and disposed of by the study site should be recorded on the Drug Inventory Log.

Statistical Methods

Primary Analysis

The primary efficacy variable is IDFS, defined as the time between randomization and date of first occurrence of an IDFS event. Patients who have not had an event will be censored at the date they are last known to be alive and event free on or prior to the clinical data cutoff date.

The log-rank test, stratified by the protocol-defined stratification factors (clinical stage at presentation [inoperable vs. operable]; hormone receptor status [ER or PR positive vs. ER and PR negative/unknown]; preoperative HER2-directed therapy [trastuzumab vs. trastuzumab plus additional HER2-directed agent(s)]; and pathologic nodal status evaluated after preoperative therapy [node positive vs. node negative/not done]), will be used to compare IDFS between the 2 treatment arms. The unstratified log-rank test results will also be provided as a sensitivity analysis. Cox proportional hazards model, stratified by the protocol-defined stratification factors, will be used to estimate the HR between the 2 treatment arms and its 95% confidence interval (CI). The Kaplan-Meier approach will be used to estimate 3-year IDFS rates and corresponding 95% CIs for each treatment arm.

Determination of Sample Size

The sample size of the study is primarily driven by the analysis of IDFS. To detect a hazard ratio (HR) of 0.75 in IDFS (a 6.5% improvement in 3-year IDFS from 70% in the control arm to 76.5% in the trastuzumab emtansine arm), approximately 384 IDFS events will be required to achieve 80% power at a 2-sided significance level of 5%. Approximately 1484 patients will be enrolled in the study.

The study is expected to be fully enrolled around 35 months after the first patient enrolls in the study (FPI). The final IDFS analysis will be performed after approximately 384 events have occurred, which is projected to be approximately 64 months from FPI.

With the study sample size of 1484 patients and approximately 10 years of follow-up, this study has about 56% power to detect an HR of 0.8 (a 2.8% improvement in 3-year OS from 85% in the control arm to 87.8% in the trastuzumab emtansine arm) at a 2-sided significance level of 5%.

Interim Analyses

One interim analysis of IDFS and 3 interim analyses of OS are planned.

The interim efficacy analysis of IDFS is planned after 67% of the targeted IDFS events have occurred, which is estimated to be approximately 48 months after the first patient is enrolled in the study. At this interim analysis, IDFS will be tested at the significance level determined using the Lan–DeMets alpha spending function with an O'Brien–Fleming boundary so that the overall 2-sided type I error rate will be maintained at the 5% level for the IDFS primary endpoint. A summary of the planed IDFS analyses is shown in the table below:

Analysis of IDFS	No. of events	Efficacy Stopping Boundary [a]	Estimated Timing [b]
Interim	257	$p < 0.0124$ or observed HR < 0.732	48 months
Final	384	$p < 0.0462$ or observed HR < 0.816	64 months

HR = hazard ratio; IDFS = invasive disease-free survival.

[a] p-value will be based on 2-sided stratified log-rank test.

[b] Time from the enrollment of first patient to data cutoff.

The purpose of the interim analysis is to evaluate whether there is an overwhelming difference in the efficacy observed in the trastuzumab emtansine arm compared with the trastuzumab arm in terms of IDFS. If the test is not significant, the study will continue as planned. If the test is significant, the independent Data Monitoring Committee (iDMC) may recommend releasing the primary endpoint results before the targeted number of 384 events is reported. In this latter situation, the Sponsor will be unblinded to the study results and a full data package would be prepared for discussion with regulatory authorities. The study will continue until 10 years of follow-up and IDFS analysis will be updated when 384 IDFS events have occurred.

Three formal interim OS analyses and one final OS analysis are planned, as detailed in the table below. The final OS analysis will be performed at the end of 10 years of follow-up. A survival data sweep will be conducted prior to each analysis.

The overall type I error will be controlled at 0.05 for the formal OS interim analyses and final OS analysis using the Lan–DeMets alpha spending function with an O'Brien–Fleming boundary. The boundaries used at each interim and final OS analysis will depend on the timing of the analyses and the number of death events actually included in the analyses.

Analysis Of OS	No. Of Events	Efficacy Stopping Boundary [a]	Estimated Timing [b]
Interim 1 (at interim IDFS)	150	$p < 0.0009$ or observed HR < 0.5826	48 months
Interim 2 (at final IDFS)	206	$p < 0.0053$ or observed HR < 0.6785	64 months
Interim 3	279	$p < 0.0184$ or observed HR < 0.754	88 months
Final	367	$p < 0.0435$ or observed HR < 0.8099	119 months

HR = hazard ratio; IDFS = invasive disease-free survival; OS = overall survival.

[a] p-value will be based on 2-sided stratified log-rank test.

[b] Time from the enrollment of first patient to data cutoff.

An iDMC will monitor accumulating patient safety data at least once every 6 months until the last patient has completed study treatment. In addition, data on SAEs and deaths will be monitored by the iDMC at least once every 3 months during this period.

After the first 600 patients have been randomized and followed up for 3 months (approximately 21 months after FPI), the iDMC will perform an interim safety analysis regarding death and hepatic events. The Clinical Events Committee will communicate their findings regarding hepatic events to the iDMC to aid iDMC review.

If an absolute increase of > 3% in the percentage of death (from any cause) or in the percentage of Hy's law cases (confirmed by the independent clinical events committee) is observed in the trastuzumab emtansine arm compared with the trastuzumab arm, the iDMC will consider recommending holding enrollment for further data review, stopping, or modifying the trial.

If an absolute increase of > 3% in the percentage of Hy's law cases (confirmed by the independent clinical events committee) is observed in the trastuzumab emtansine arm compared with the control arm, the iDMC will consider recommending holding enrollment for further data review, stopping, or modifying the trial.

The iDMC will work according to the guidelines defined in the iDMC Charter. The iDMC Charter will contain details regarding the frequency of meetings, guidelines for decision making, and process for requesting further information. The iDMC members will review and sign off on the charter before the first review.

LIST OF ABBREVIATIONS AND DEFINITIONS OF TERMS

Abbreviation	Definition
AE	adverse event
ALK	alkaline phosphatase
ALT	alanine aminotransferase
aPTT	activated partial thromboplastin time
AST	aspartate aminotransferase
ATA	anti-therapeutic antibody
CHF	congestive heart failure
CI	confidence interval
CIS	carcinoma in situ
CTCAE	Common Terminology Criteria for Adverse Events
DCIS	ductal carcinoma in situ
DFS	disease-free survival
DRFI	distant recurrence-free interval
EC	Ethics Committee
ECHO	echocardiogram
ECOG	Eastern Cooperative Oncology Group
eCRF	electronic Case Report Form
EDC	electronic data capture
ePRO	electronic patient-reported outcome
EORTC	European Organisation for Research and Treatment of Cancer
ER	estrogen receptor
FACT-B	Functional Assessment of Cancer Therapy-Breast
FDA	Food and Drug Administration
FISH	fluorescence in situ hybridization
FPI	first patient enrolls in the study
HIPAA	Health Insurance Portability and Accountability Act
HR	hazard ratio
HRQOL	health-related quality of life
IB	Investigator's Brochure
ICH	International Conference on Harmonisation
iDCC	independent Data Coordinator Center
iDMC	independent Data Monitoring Committee
IDFS	invasive disease-free survival
IHC	immunohistochemistry
IMP	investigational medicinal product

IND	Investigational New Drug (application)
INR	international normalized ratio
IRB	Institutional Review Board
ISH	in situ hybridization
IV	intravenous
IVRS/IWRS	interactive voice response system/interactive web response system
LCIS	lobular carcinoma in situ
LFT	liver function laboratory test
LVEF	left ventricular ejection fraction
MUGA	multiple-gated acquisition
NCI	National Cancer Institute
NSABP	National Surgical Adjuvant Breast and Bowel Project
NRH	nodular regenerative hyperplasia
NYHA	New York Heart Association
OS	overall survival
pCR	pathological complete response
PD	progressive disease
PK	pharmacokinetic
PR	progesterone receptor
PRO	patient-reported outcome
q3w	every 3 weeks
QLQ-C30	Quality of Life Questionnaire – Core 30
QLQ-BR23	Quality of Life Questionnaire – Breast Cancer 13
RCR	Roche Clinical Repository
SAE	serious adverse event
TBILI	total bilirubin
ULN	upper limit of normal

1. BACKGROUND

1.1 BACKGROUND ON EARLY-STAGE HER2-POSITIVE BREAST CANCER

The use of adjuvant (postoperative) trastuzumab in HER2-positive early-stage breast cancer improves patient outcomes as demonstrated in several large, randomized trials. The 3-year disease-free survival (DFS) rate for patients receiving trastuzumab in these studies, all of whom had operable disease, was approximately 85% to 90% (Romond et al. 2005; Piccart-Gebhart 2005; Slamon et al. 2011). A variety of trastuzumab-based chemotherapy regimens are considered effective for the treatment of non-metastatic HER2-positive breast cancer. These include doxorubicin and cyclophosphamide followed by a taxane (docetaxel or paclitaxel) plus trastuzumab (AC-TH); docetaxel, carboplatin, and trastuzumab (TCbH); and 5-fluorouracil, epirubicin, and cyclophosphamide in sequence with docetaxel plus trastuzumab (FEC-TH or TH-FEC). These chemotherapy regimens for early-stage HER2-positive breast cancer share in common the following features: 1) at least 6 total cycles of chemotherapy; 2) at least 9 weeks of trastuzumab in combination with a taxane; and 3) subsequent trastuzumab monotherapy to complete a total of 1 year of adjuvant HER2-directed treatment. U.S. and E.U. labeling for Herceptin® (trastuzumab) recommends a total of 12 months of treatment (as adjuvant therapy in the United States and as neoadujvant and/or adjuvant therapy in the European Union); this is endorsed by local practice guidelines (Gnant et al. 2011; NCCN 2011). Such regimens used in the adjuvant setting are also recommended for preoperative (also known as “neoadjuvant”) therapy, as the timing of chemotherapy administration in relation to primary surgery does not impact survival (Gnant et al. 2011; NCCN 2011).

For patients with operable breast cancer, preoperative therapy has been shown in several randomized trials to result in survival outcomes similar to those with adjuvant therapy, with the added benefit of improving breast conservation rates (Mauri et al. 2005). In trials of preoperative therapy, it has been consistently demonstrated that patients who achieve a pathological complete response (pCR) have an improved prognosis compared with those who have residual invasive disease present in the surgical specimen after completion of preoperative therapy (non-pCR). For example, in the National Surgical Adjuvant Breast and Bowel Project (NSABP) Studies B-18 and B-27, the hazard ratios (HRs) for DFS for patients who achieved a pCR compared with those who did not were 0.47 and 0.49, respectively,; the HRs for overall survival (OS) were 0.32 and 0.36, respectively (Rastogi et al. 2008). The U.S. Food and Drug Administration (FDA) has recently issued draft guidance on the use of pCR as a surrogate endpoint to support accelerated approval. While there are only limited data for preoperative therapy conducted exclusively in patients with HER2-positive breast cancer, in general, [redacted], and patients who attain a pCR have a more favorable prognosis than those who have residual invasive disease (Buzdar et al. 2005; Gianni et al. 2010; [redacted]; Loibl et al. 2011). For example, the addition of trastuzumab to 24 weeks of sequential paclitaxel-

FEC improved pCR from 25% to 66.7% in patients with operable HER2-positive breast cancer (Buzdar et al. 2005). The Neoadjuvant Herceptin® (NOAH) study randomized patients with locally advanced or inflammatory HER2-positive cancer to preoperative chemotherapy with or without trastuzumab (Gianni et al. 2010). [redacted] In the Taxol® Epirubicin Cyclophosphamide Herceptin Neoadjuvant (TECHNO) study, which included patients with either operable or inoperable HER2 positive breast cancer, pCR following preoperative trastuzumab-based therapy was statistically significantly associated with both improved DFS and OS in multivariate analyses, and the 3-year DFS rate was approximately 70% for non-pCR patients compared to 88% for those attaining pCR (Untch et al. 2011). A recent meta-analysis of neoadjuvant studies affirmed the prognostic import of pCR in 662 HER2-positive patients who had received trastuzumab. With pCR defined as no invasive or non-invasive residual disease in the breast or lymph nodes, there was a significant benefit in OS for attainment of pCR ($p < 0.0001$) (Loibl et al. 2011). Therefore, the absence of pCR after appropriate neoadjuvant therapy allows identification of a patient population at higher risk of disease recurrence. This is a clinical setting where the application of more effective therapies would have a potentially large absolute impact on patient outcomes, and can be considered an area of unmet medical need.

While it is recognized that patients without pCR after preoperative treatment are at increased risk of recurrence, no specific adjuvant regimens are recommended for this population. Additional systemic therapy for non-pCR patients has not been rigorously studied or shown to be of benefit. Therefore, these patients are currently recommended to receive the same adjuvant therapies as would be used for any patient with HER2-positive breast cancer, regardless of surgical findings (NCCN 2011; Gnant et al. 2011). For example, patients with HER2-positive breast cancer are recommended to complete a total of 1 year of trastuzumab treatment. They may also receive post-surgical radiation as part of breast conservation or for the presence of other high-risk features. In addition, those with hormone receptor–positive disease are recommended to receive hormonal therapy after surgery.

Various definitions of pCR have been commonly utilized in clinical trials, but all have demonstrated prognostic value. In the earliest studies of preoperative therapy, the absence of residual disease in the breast alone was mainly considered. More recently, the status of the axillary lymph nodes has also been considered. There are conflicting data on whether residual in-situ disease carries prognostic significance in the absence of residual invasive disease. Thus, a conservative definition of pCR would be the absence of residual invasive disease in the breast or axillary lymph nodes. Attempts have been made to further refine prognostic categories for patients whose tumors do not achieve a pCR after preoperative treatment. However, no additional factors have been validated to date.

In summary, preoperative chemotherapy in combination with trastuzumab is a standard of care for patients with HER2-positive locally advanced (Stage IIB to IIIC) breast cancer or in cases where patients wish to minimize the extent of breast cancer surgery. Compared with patients who attain a pCR after preoperative therapy, patients with residual disease have a greater risk of recurrence and death. It is not known whether the application of additional non–cross-resistant agents in the adjuvant setting may benefit these patients, and there are no approved therapies in this clinical setting. Because trastuzumab emtansine has shown activity in patients who have previously progressed after chemotherapy and HER2-directed therapy in the metastatic setting, it would be reasonable to explore in a clinical study whether there may be a benefit of administering trastuzumab emtansine to patients with HER2-positive early breast cancer who have not had an optimal response to commonly recommended preoperative therapy regimens.

1.2 BACKGROUND ON TRASTUZUMAB EMTANSINE

Trastuzumab emtansine is a novel antibody-drug conjugate (ADC), specifically designed for the treatment of HER2-positive cancer. It is composed of the following components: trastuzumab, a humanized antibody directed against the extracellular region of HER2; DM1, an anti-microtubule agent derived from maytansine; and succinimidyl 4-[N-maleimidomethyl] cyclohexane-1-carboxylate (SMCC), a thioether linker molecule used to conjugate DM1 to trastuzumab. Trastuzumab emtansine binds to HER2 with an affinity similar to that of unconjugated trastuzumab. It is hypothesized that after binding to HER2, trastuzumab emtansine undergoes receptor-mediated internalization, resulting in intracellular release of DM1 and subsequent cell death. DM1 is an inhibitor of tubulin polymerization; it binds to tubulin competitively with vinca alkaloids.

Data from clinical trials of trastuzumab emtansine that are especially relevant to the design of the current trial are summarized below. Please refer to the most recent version of the trastuzumab emtansine Investigator's Brochure (IB) for further information on all of the completed and ongoing trastuzumab emtansine studies.

1.2.1 Study TDM4450g/BO21976

Study TDM4450g/BO21976 is a randomized, multicenter, Phase II trial of the efficacy and safety of trastuzumab emtansine (3.6 mg/kg intravenous [IV] every 3 weeks [q3w]) versus trastuzumab plus docetaxel in patients with metastatic HER2-positive breast cancer who have not received prior chemotherapy for metastatic disease.

The primary endpoints were investigator-assessed PFS and safety. The primary PFS analysis took place after 72 investigator-assessed PFS events had occurred in the 2 arms combined (data cut: 15 November 2010). ORR, duration of objective response, and related exploratory assessments; and patient-reported outcome (PRO) were also analyzed at this time. The OS and safety analyses were performed approximately 24

months after the last patient was enrolled (data cut: 31 August 2011). A total of 137 patients were enrolled in the study.

Trastuzumab emtansine demonstrated significant improvement in PFS over trastuzumab plus docetaxel as first-line MBC therapy. The median PFS was 14.2 months in the trastuzumab emtansine arm compared with 9.2 months in the trastuzumab plus docetaxel arm (HR = 0.59; 95% confidence interval (CI) = 0.364-0.968; log-rank p-value = 0.035), with a median follow-up of approximately 14 months in both arms. The ORR was 58.0% with trastuzumab plus docetaxel and 64.2% with trastuzumab emtansine. Tumor response was more durable with trastuzumab emtansine (median duration of response not reached vs. median duration 9.5 months in the control arm). Compliance rate on QOL measures was high (≥93%) across cycles for both treatment arms. The worsening of the Functional Assessment of Cancer Therapy-Breast (FACT-B) Trial Outcome Index scores was delayed in the trastuzumab emtansine arm compared with the control arm (7.5 vs. 3.5 months; HR = 0.58; p = 0.022). Preliminary results indicate that OS results are similar between the arms. However, OS data was not mature (less than 20% of the patients in the study had died at the time of the data cut and the median duration of OS was not reached for either arm). Results were also potentially confounded by crossover as allowed in the protocol. At the time of OS follow-up, there were 35 patients (50%) from the trastuzumab plus docetaxel arm who had crossed over to trastuzumab emtansine after documented disease progression.

Grade ≥ 3 AEs were reported less frequently in the trastuzumab emtansine group compared with the trastuzumab plus docetaxel group (46.4% vs. 90.9%), as were AEs leading to treatment discontinuations (7.2% vs. 34.8%) and SAEs (20.3% vs. 25.8%).

1.2.2 Study TDM4874g/BO22857

Study TDM4874g/BO22857 is an ongoing multicenter, multinational, single arm Phase II study to assess the safety and feasibility of administering trastuzumab emtansine (3.6 mg/kg IV q3w) after anthracycline-based chemotherapy as adjuvant or preoperative therapy for patients with early-stage HER2-positive breast cancer. Patients must complete an anthracycline-based regimen (FEC or AC) and initiate treatment with trastuzumab emtansine within 42 days. The safety objectives of the study include the following: to evaluate the rate of pre-specified cardiac events (New York Heart Association [NYHA] class III/IV congestive heart failure [CHF]) following initiation of trastuzumab emtansine treatment; to evaluate the safety and feasibility of trastuzumab emtansine when given with concurrent radiotherapy; to evaluate the feasibility of the planned duration (up to 17 cycles) of treatment with trastuzumab emtansine therapy; and to evaluate the safety profile of trastuzumab emtansine in this patient population. Real-time monitoring of AEs is ongoing. At the primary analysis (Dang et al. 2012), with a data cutoff of October 28, 2011, 148 patients had received at least 1 cycle of trastuzumab emtansine. There were no prespecified cardiac events, with a cardiac event rate of 0.0% (95% CI 0.0% to 2.45%). There were no reported AEs of left

ventricular dysfunction or heart failure. One patient experienced a dose delay due to asymptomatic left ventricular ejection fraction (LVEF) decrease; no patient discontinued trastuzumab emtansine due to cardiac cause. Twenty patients have received treatment with concurrent RT and trastuzumab emtansine. There was no apparent effect of concurrent delivery on the ability to give trastuzumab emtansine and radiotherapy at planned doses. During concurrent RT, there was no Grade 3 or higher skin toxicity; one patient experienced a Grade 2 RT-related pneumonitis that resolved with corticosteroids. Six patients discontinued trastuzumab emtansine due to AEs: one with Grade 3 increase in liver transaminases (aspartate aminotransferase [AST]) and one with Grade 3 neutropenia; one case each of Grade 2 events of malaise, vertigo, alanine aminotransferase (ALT) and AST increase, and thrombocytopenia. The study is ongoing.

1.2.3 Study TDM4370g/BO21977 (EMILIA)

Study TDM4370g/BO21977 is a randomized Phase III study of trastuzumab emtansine versus capecitabine and lapatinib for patients with HER2-positive unresectable locally advanced or metastatic breast cancer (MBC) previously treated with trastuzumab and a taxane. Patients received trastuzumab emtansine (3.6 mg/kg IV q3w) or capecitabine (1000 mg/m^2 PO bid, Days 1 to 14 q3w) + lapatinib (1250 mg PO daily) until progressive disease (PD) or unmanageable toxicity. Patients had confirmed HER2-positive MBC (immunohistochemistry [IHC] 3+ and/or fluorescence in situ hybridization [FISH+]) and prior therapy with trastuzumab and a taxane. Primary endpoints were PFS by independent review, OS, and safety. An interim OS analysis was planned at the time of the final PFS analysis. A total of 991 patients were enrolled; 978 received treatment. Median durations of follow-up were 12.9 (trastuzumab emtansine) and 12.4 (capecitabine/lapatinib) months. Baseline patient demographics, prior therapy, and disease characteristics were balanced. There was a significant improvement in PFS favoring trastuzumab emtansine (HR = 0.650, $p < 0.0001$; median 9.6 vs. 6.4 months). A strong trend in OS was observed in favor of the trastuzumab emtansine arm (HR = 0.621, $p = 0.0005$, median not reached vs 23.3 months); however, the interim efficacy stopping boundary for OS was not crossed. The objective response rate was 43.6% for trastuzumab emtansine versus 30.8% for capecitabine/lapatinib with the median duration of response being 12.6 months versus 6.5 months respectively. Trastuzumab emtansine was well tolerated with no unexpected safety signals. The most common grade ≥ 3 AEs for trastuzumab emtansine were: thrombocytopenia (12.9% vs. 0.2%), increased AST (4.3% vs. 0.8%), and increased ALT (2.9% vs. 1.4%); and for capecitabine/lapatinib were: diarrhea (20.7% vs. 1.6%), palmar plantar erythrodysesthesia (16.4% vs. 0) and vomiting (4.5% vs. 0.8%). The incidence of Grade ≥ 3 AEs in the trastuzumab emtansine arm was 40.8% versus 57.0% for capecitabine/lapatinib. Median time to symptom progression, as defined by a 5-point decrease in the score derived from the trial outcome index-breast (TOI-B) subscale of the FACT-B quality of life questionnaire was delayed in female patients receiving trastuzumab emtansine compared to those receiving lapatinib plus capecitabine (7.1

months for trastuzumab emtansine, compared to 4.6 months: HR = 0.796, 95% CI = 0.667-0.951; p = 0.0121).

1.2.4 Study TDM4788g/BO22589 (MARIANNE)

Study TDM4788g/BO22589 is a randomized 3 arm, Phase III study of trastuzumab emtansine combined with pertuzumab versus trastuzumab emtansine combined with pertuzumab-placebo (blinded for pertuzumab) versus trastuzumab plus taxane as first-line treatment in HER2-positive progressive or recurrent locally advanced or metastatic breast cancer patients. The primary objectives of the study are to compare the efficacy (PFS) and safety across the 3 arms. The enrollment has been completed with a total of 1095 patients, and the study is ongoing. Unblinded safety and efficacy data are not yet available to the Sponsor. However, the independent Data Monitoring Committee (iDMC) reviews data from the trial on a quarterly basis and has thus far recommended that the study should continue as planned.

1.2.5 Studies TDM4258g and TDM4374g

The efficacy of trastuzumab emtansine (at a dose of 3.6 mg/kg q3w) was evaluated in 2 completed single-arm Phase II studies, TDM4258g and TDM4374g. Study TDM4258g enrolled patients with HER2-positive MBC who had progressed on previous HER2-directed therapy, while patients in study TDM4374g had disease progression after at least 2 HER2-directed therapies (i.e., trastuzumab and lapatinib) in the metastatic or locally advanced setting. In both of these studies, the primary efficacy endpoint was objective response as assessed by independent review of tumor assessments. The clinical activity of trastuzumab emtansine was similar in the 2 studies, with an objective response rate of 26% in TDM4258g and 32% in TDM4374g. A pooled analysis of the safety profile of trastuzumab emtansine monotherapy in previously treated metastatic breast cancer patients from these phase II studies is available in the IB.

1.3 BACKGROUND ON TRASTUZUMAB (HERCEPTIN®)

Trastuzumab is a recombinant humanized anti-p185 HER2 monoclonal antibody that binds with high affinity to the HER2 protein. U.S. and E.U. labeling for Herceptin® (trastuzumab) recommends a total of 12 months of treatment as adjuvant therapy in the United States and as neoadujvant and/or adjuvant therapy in the European Union based on the data generated in randomized trials (Piccart-Gebhart 2005; Romond et al. 2005; Slamon 2011; Gianni et al. 2010)

Details on the AEs associated with trastuzumab and clinical use of trastuzumab are to be found in the trastuzumab IB and in the Herceptin local prescribing information.

1.4 STUDY RATIONALE AND BENEFIT RISK ASSESSMENT

Breast cancer patients who do not achieve a pCR following preoperative treatment are at increased risk of recurrence and breast cancer–related death compared with those that do achieve a pCR. Trastuzumab emtansine has shown activity and a favorable

benefit-risk profile in patients who have progressed after prior HER2-directed therapies for metastatic disease. In addition, trastuzumab emtansine appears to have a favorable benefit-risk profile in patients who have not received prior chemotherapy for metastatic disease, including patients who have previously received trastuzumab in the adjuvant setting. The safety profile of trastuzumab emtansine appears to be acceptable in the metastatic setting and is currently under evaluation for early-stage HER2-positive breast cancer. In the curative treatment setting, certain acute AEs or potential chronic organ effects (e.g., cardiac and/or hepatic damage) may constitute a specific concern for trastuzumab emtansine. In studies done to date, there does not appear to be an increased risk for cardiac AEs with trastuzumab emtansine as compared to other HER2-directed therapies. Increases in transaminases are observed with the administration of trastuzumab emtansine but the potential for severe acute drug-induced liver injury is not clear. While initial safety data to date using trastuzumab emtansine in previously untreated patients in both the adjuvant (Dang et al. 2012) and metastatic setting (Hurwitz 2011) appears acceptable, a safety monitoring plan for this study including appropriate eligibility criteria, dose modification guidelines, and interim safety analyses, as well as regular monitoring of accumulating patient safety data by a Data Monitoring Committee has been put in place to minimize any potential risk in the trial patient population.

2. OBJECTIVES

2.1 PRIMARY EFFICACY OBJECTIVE

The primary efficacy objective for this study is as follows:

- To compare invasive disease-free survival (IDFS, Section 3.4.1) in patients with residual invasive breast cancer after treatment with preoperative chemotherapy and HER2-directed therapy including trastuzumab followed by surgery between the 2 treatment arms

The secondary efficacy objective for this study is as follows:

- To compare IDFS including second non-breast cancers, DFS, OS, and distant recurrence-free interval (DRFI) between the 2 treatment arms

2.2 SAFETY OBJECTIVES

The safety objective for this study is as follows:

- To compare cardiac safety and overall safety between the 2 treatment arms according to the National Cancer Institute Common Terminology Criteria for Adverse Events (NCI CTCAE), Version 4.0

2.3 PATIENT REPORTED OUTCOME OBJECTIVES

The PRO objective for this study is as follows:

- To compare PROs between the 2 treatment arms using the European Organisation for Research and Treatment of Cancer (EORTC) Quality of Life Questionnaire – Core 30 (QLQ-C30) questionnaire and Quality of Life Questionnaire – Breast Cancer 13 (QLQ-BR23) module

2.4 PHARMACOKINETICS OBJECTIVES

The pharmacokinetics (PK) objectives for this study are as follows:

- To characterize the PK of trastuzumab emtansine (including total trastuzumab and DM1) in trastuzumab emtansine treated patients
- To characterize the PK of trastuzumab in trastuzumab-treated patients and permit an intra-study comparison of trastuzumab exposure in the 2 treatment arms
- To investigate exposure–effect (efficacy and safety) relationships in this patient population

2.5 EXPLORATORY OBJECTIVES

The exploratory objectives for this study are as follows:

- To assess correlations between biomarker status and efficacy and/or safety
- To assess the incidence of anti-therapeutic antibodies (ATAs) and the effect of ATAs on PK, safety, and efficacy

3. STUDY DESIGN

3.1 DESCRIPTION OF STUDY

3.1.1 Overview

Study BO27938 is a Phase III, 2-arm, randomized, multicenter, multinational, open-label study in patients with HER2-positive primary breast cancer who have received preoperative chemotherapy and HER2-directed therapy including trastuzumab followed by surgery, with a finding of residual invasive disease in the breast or axillary lymph nodes.

Patients will be randomized to one of the following treatment arms in a 1:1 ratio:

Arm A: Trastuzumab emtansine 3.6 mg/kg q3w for 14 cycles

Arm B: Trastuzumab 6 mg/kg q3w for 14 cycles (an 8 mg/kg loading dose should be given in cases where there has been an interval greater than 6 weeks since the last dose of trastuzumab)

The study will enroll approximately 1484 patients who have pathologically documented residual invasive disease in either the breast or axillary lymph nodes following completion of preoperative therapy. Patients must have received at least 9 weeks of HER2-directed therapy including trastuzumab, at least 9 weeks of taxane therapy, and at

least 16 weeks of total systemic treatment in the preoperative setting. Patients may also have received anthracycline-based preoperative therapy, in accordance with local standards.

Patients will receive study treatment for a maximum of 14 cycles; treatment will be discontinued prior to 14 cycles in the event of disease recurrence, unacceptable toxicity, or study termination by the Sponsor. Patients who discontinue trastuzumab emtansine may complete the duration of their study therapy with trastuzumab if appropriate based on toxicity considerations. Following discontinuation or completion of study treatment, patients will continue to be followed for efficacy and safety objectives until the end of the study.

Radiotherapy and/or hormonal therapy (for patients with hormone receptor-positive tumors) concurrent with study treatment should be administered if indicated based on the following guidelines (refer to Section 4.1.1.1 for lymph node surgery requirements):

- Hormonal therapy (aromatase inhibitor, tamoxifen, etc.) should be initiated in patients with hormone receptor-positive disease at presentation.
- For patients undergoing breast-conserving surgery, whole breast irradiation is required. Regional node irradiation is required if the patient presented at initial diagnosis with clinical T3 or T4 disease and/or with clinical N2 or N3 disease; it is recommended if there is residual disease in lymph nodes.
- For post-mastectomy patients, chest wall and regional node irradiation is required if the patient presented at initial diagnosis with clinical T3 or T4 disease and/or with clinical N2 or N3 disease; it is recommended if there is residual disease in lymph nodes. For post-mastectomy patients who do not meet these criteria, radiotherapy is at the discretion of the investigator based on institutional standards.

A permuted-block randomization scheme will be used to ensure an approximate 1:1 allocation of patients to receive trastuzumab emtansine or trastuzumab with respect to the following stratification factors:

- Clinical stage at presentation: inoperable (Stage T4NxM0 or TxN2–3M0) versus operable (Stages T1-3N0–1M0)
- Hormone receptor status: estrogen receptor (ER) or progesterone receptor (PR) positive versus ER and PR negative/unknown
- Preoperative HER2-directed therapy: trastuzumab versus trastuzumab plus additional HER2-directed agent(s)
- Pathologic nodal status evaluated after preoperative therapy: node positive versus node negative/not done

Schedules of assessments are provided in Appendix 1 and Appendix 2.

3.1.2 Data Monitoring Committee

An iDMC will monitor accumulating patient safety data at least once every 6 months until the last patient has completed study treatment. In addition, data on SAEs and deaths will be monitored by the iDMC at least once every 3 months during this period. The iDMC will also assess safety and efficacy as part of the interim efficacy and safety analyses. An independent Data Coordinator Center (iDCC) will perform unblinded analyses to support the periodic iDMC review of safety data and the interim analysis. Additional details will be provided in an iDMC Charter.

3.1.3 Clinical Events Committee

An independent safety advisory board will adjudicate pre-specified safety events of interest (cardiac and hepatic dysfunction events). A separate charter will outline the committee's composition, meeting timelines, and members' roles and responsibilities. The committee members may review all potential cases of CHF and cardiac death as well as all potential cases of hepatic dysfunction and Hy's law. Identified cases will be forwarded to the iDMC on a regular basis as part of the ongoing safety reviews.

3.2 END OF STUDY

The study will end after the last patient randomized into the study has undergone the last follow-up assessment. To enable long-term follow-up for survival and safety information, the last follow-up assessment is scheduled to occur 10 years after the first patient is randomized.

3.3 RATIONALE FOR STUDY DESIGN

3.3.1 Rationale for Test Product Dosage

In a Phase I study (TDM3569g), the maximum tolerated dose of trastuzumab emtansine administered by IV infusion q3w was 3.6 mg/kg. Clinical activity has been observed at a dose of 3.6 mg/kg q3w in studies of single-agent trastuzumab emtansine in both pretreated and previously untreated HER2-positive MBC. In the adjuvant or neoadjuvant setting following anthracycline therapy, trastuzumab emtansine 3.6 mg/kg q3w has been tolerated as a single agent administered up to 17 cycles without evidence of significant cardiac toxicity. Please refer to the trastuzumab emtansine IB for further information.

There are 2 approved dose regimens of trastuzumab: a 4 mg/kg loading dose followed by a 2 mg/kg dose every week; and an 8 mg/kg loading dose followed by a 6 mg/kg dose q3w. The half-life of trastuzumab has been determined to be approximately 28.5 days, which supports a dosing of q3w. Data to support the q3w regimen are available from 2 studies evaluating the safety, tolerability, and PK of trastuzumab administered to women with HER2-positive (IHC 3+ or FISH+) metastatic breast cancer and from the 1-year arm of the HERA study (BO16348). Data from these three trials indicate that serum concentrations of trastuzumab increased until steady-state trough concentrations (median 47.3 ng/mL, 95% CI 19.6 to 51.2 ng/mL). The average exposure at any time during the treatment is comparable between the 2 treatment regimens.

Please refer to the trastuzumab IB for further information.

3.3.2 Rationale for Patient Population

Preoperative/neoadjuvant systemic therapy has been shown to be equivalent to adjuvant therapy in terms of long-term disease outcomes in randomized trials. It may be utilized to improve operability or to shrink tumors to enable breast conservation at the discretion of patients and their physicians. Patients without a pCR following preoperative treatment are at increased risk of recurrence and breast cancer–related death compared with those that do achieve a pCR. A number of questions remain regarding treatment duration and exactly how to combine HER2-directed therapy with chemotherapy in the neoadjuvant setting. A randomized Phase II study in HER2-positive patients demonstrated that the rate of pCR was equivalent using anthracycline and non-anthracycline based chemotherapy regimens (Schneeweiss et al. 2011). In addition, the administration of HER2 targeting agents trastuzumab and pertuzumab with a taxane for 3 cycles following anthracycline resulted in similar pCR rates to administration of 6 cycles of these HER2 targeting agents initially given concurrently with anthracycline. Therefore, patients who have received both anthracycline and non-anthracycline based neoadjuvant regimens may enroll in this study, with a minimum requirement of 9 weeks of exposure to a taxane and trastuzumab and to 6 cycles of neoadjuvant chemotherapy.

3.3.3 Rationale for Control Group and Duration of Therapy

Although 1 year of trastuzumab therapy is currently recommended for HER2-positive patients, both shorter and longer durations of therapy are being investigated in the adjuvant setting. By standardizing the study treatment duration to 14 cycles, given q3w, and requiring at least 9 weeks of preoperative trastuzumab, all patients should receive at minimum approximately 1 year of HER2-directed therapy, consistent with current practice guidelines, as tolerated.

3.3.4 Rationale for Biomarker Assessments

For all patients, IHC and/or in situ hybridization (ISH) assays will be performed for the mandatory confirmation of HER2 status. Other in situ methods or newly available diagnostics to evaluate HER2 may be applied as well on these samples.

HER2 signaling is known to be modulated by expression levels of other HER family members (e.g., HER1 and HER3) and their ligands, the expression of which may correlate with response or resistance to HER2-targeted therapies. As an exploratory objective, the expression of other HER family receptors or ligands and their potential impact on clinical efficacy will be assessed in this study, should sufficient material be available. Additional candidate markers of response to trastuzumab emtansine that emerge from other clinical or preclinical studies may also be assessed in this study.

3.3.5 Rationale for Pharmacokinetic Assessments

The rationale for collecting PK samples is to further characterize the PK of trastuzumab emtansine, total trastuzumab, and DM1 in trastuzumab emtansine treated patients and to establish correlations between drug exposure and measures of both efficacy and toxicity in the adjuvant breast cancer setting. The proposed PK sampling scheme should allow for adequate characterization of each analyte for the planned analysis. Any remaining serum/plasma samples may be, at Sponsors discretion and if stability is confirmed, used for measurement of trastuzumab emtansine metabolites as an exploratory assessment.

Among patients randomized to receive trastuzumab, the rationale for collecting PK samples is to assess the PK of trastuzumab in this patient population and the sampling scheme will allow an intra-study comparison of trastuzumab exposure in the 2 treatment arms.

3.4 OUTCOME MEASURES

3.4.1 Primary Efficacy Outcome Measure

The primary efficacy outcome measure is IDFS, defined as the time from randomization until the date of the first occurrence of any one of the following events:

- Ipsilateral invasive breast tumor recurrence (i.e., an invasive breast cancer involving the same breast parenchyma as the original primary lesion)
- Ipsilateral local-regional invasive breast cancer recurrence (i.e., an invasive breast cancer in the axilla, regional lymph nodes, chest wall and/or skin of the ipsilateral breast)
- Distant recurrence (i.e., evidence of breast cancer in any anatomic site—other than the 2 above-mentioned sites—that has either been histologically confirmed or clinically diagnosed as recurrent invasive breast cancer)
- Contralateral invasive breast cancer
- Death attributable to any cause including breast cancer, non-breast cancer or unknown cause (but cause of death should be specified if at all possible)

3.4.2 Secondary Efficacy Outcome Measures

Secondary efficacy outcome measures include the following:

- IDFS including second primary non-breast cancer: defined the same way as IDFS for the primary endpoint but including second primary non-breast invasive cancer as an event (with the exception of non-melanoma skin cancers and carcinoma in situ [CIS] of any site)
- DFS: defined as the time between randomization and the date of the first occurrence of an IDFS event including second primary non-breast cancer event or contralateral or ipsilateral ductal carcinoma in situ (DCIS)

- OS: defined as the time from randomization to death due to any cause
- DRFI: defined as the time between randomization and the date of distant breast cancer recurrence

3.4.3 Safety Outcome Measures

Clinical and laboratory AEs will be reported according to the NCI CTCAE, Version 4.0. LVEF will be assessed using either echocardiogram (ECHO) or multiple-gated acquisition (MUGA).

Safety will be measured by determining the incidence, nature, and severity of AEs. The safety outcome measures are the following protocol-specific AEs:

- Incidence, type and severity of all AEs based on NCI CTCAE Version 4.0
- Incidence, type, and severity of SAEs
- Incidence and type of AEs leading to dose discontinuation, modification, or delay
- Cause of death on study
- Abnormal laboratory values
- LVEF decreases
- Cardiac events, defined as death from cardiac cause or severe CHF (NYHA Class III or IV) with a decrease in LVEF of $\geq$10 percentage points from baseline to an LVEF of $<$50%.

3.4.4 Patient-Reported Outcome Measures

The PRO outcome measures for this study are as follows:

- Incidence of treatment-related symptoms and assessment of health-related quality of life (HRQOL) as measured using the EORTC QLQ-C30 questionnaire and QLQ-BR23 module
- Assessment of health status as measured using the EuroQol EQ-5D™ questionnaire for health economic modeling

3.4.5 Pharmacokinetic Outcome Measures

The PK outcome measures to be assessed in patients receiving trastuzumab emtansine are the following:

- Observed serum concentrations and relevant PK parameters of trastuzumab emtansine (trastuzumab emtansine conjugated) and total trastuzumab (sum of conjugated and unconjugated trastuzumab)
- Observed plasma concentrations of DM1
- Explore relationship between trastuzumab emtansine exposure and efficacy/safety
- Characterize ATA and assess impact of ATA on PK, safety and efficacy.

3.4.6 Exploratory Outcome Measure

The exploratory outcome measure for this study is as follows:

- The relationship between molecular markers and efficacy outcomes

Efficacy outcomes considered for this analysis will include IDFS and OS, as appropriate.

4. MATERIALS AND METHODS

4.1 PATIENTS

The patient population for this study will include patients with primary non-metastatic HER2-positive breast cancer (see Section 4.1.1.1 for definition).

4.1.1 Inclusion Criteria

Patients must meet the following criteria for study entry.

4.1.1.1 Disease-Specific Inclusion Criteria

1. HER2-positive breast cancer

   Positivity will be based on pretreatment biopsy and defined as IHC score of 3+ and/or positive by ISH prospectively confirmed by a central laboratory prior to study enrollment. Paraffin-embedded tumor tissue block or a partial block must be obtained. If sites are unable to send a tissue block due to local regulations, at least 8 unstained slides should be sent for HER2 testing, and in addition up to 5 slides for exploratory biomarker research. ISH positivity is defined as a ratio of $\geq$2.0 for the number of HER2 gene copies to the number of signals for CEP17. Both IHC and ISH assays will be performed; however, only one positive result is required for eligibility. In the event that sufficient material from the pretreatment biopsy is not available for submission, central HER2 determination for eligibility may be performed on residual tumor tissue from the time of definitive surgery.

   Patients with synchronous bilateral invasive disease are eligible provided both lesions are HER2-positive.

2. Histologically confirmed invasive breast carcinoma

3. Clinical stage at presentation: T1–4, N0–3, M0 (Note: Patients with T1a/bN0 tumors will not be eligible)

4. Completion of preoperative systemic treatment consisting of at least 6 cycles with a total duration at least 16 weeks, including at least 9 weeks of trastuzumab and at least 9 weeks of taxane-based chemotherapy.

   Note: HER2-directed therapy and chemotherapy may be given concurrently; patients may have received more than one HER2-directed therapy. Patients may have received an anthracycline as part of preoperative therapy.

5. Adequate excision: surgical removal of all clinically evident disease in the breast and lymph nodes as follows:

Breast surgery: total mastectomy with no gross residual disease at the margin of resection, or breast-conserving surgery with histologically negative margins of excision

For patients who undergo breast-conserving surgery, the margins of the resected specimen must be histologically free of invasive tumor and DCIS as determined by the local pathologist. If pathologic examination demonstrates tumor at the line of resection, additional operative procedures may be performed to obtain clear margins. If tumor is still present at the resected margin after re-excision(s), the patient must undergo total mastectomy to be eligible. Patients with margins positive for lobular carcinoma in situ (LCIS) are eligible without additional resection.

Lymph node surgery:

In case of positive results from a fine-needle aspiration, core biopsy, or sentinel node biopsy performed prior to preoperative therapy, additional surgical evaluation of the axilla following preoperative therapy is required.

If sentinel node biopsy performed before preoperative therapy was negative, no additional surgery evaluation of the axilla is required after preoperative therapy.

If the only sentinel node identified by isotope scan is in the internal mammary chain, surgical evaluation of the axilla is recommended.

If sentinel node biopsy performed after preoperative therapy is positive, additional surgical evaluation of the axilla is recommended.

If sentinel node evaluation after preoperativetherapy is negative, no further additional surgical evaluation of the axilla is required.

Axillary dissection without sentinel node evaluation is permitted after preoperative therapy.

6. Pathologic evidence of residual invasive carcinoma in the breast or axillary lymph nodes following completion of preoperative therapy
7. An interval of no more than 12 weeks between the date of surgery and the date of randomization
8. Known hormone receptor status (either ER and/or PR of the primary tumor)

4.1.1.2 General Inclusion Criteria

9. Signed written informed consent approved by the study site's Institutional Review Board (IRB)/Ethical Committee (EC)
10. Age $\geq$ 18 years
11. Eastern Cooperative Oncology Group (ECOG) performance status 0 or 1
12. Life expectancy $\geq$ 6 months
13. Adequate organ function during screening, defined as:

    Absolute neutrophil count $\geq$ 1200 cells/mm^3

Platelet count $\geq$ 100000 cells/mm^3

Hemoglobin $\geq$ 9.0 g/dL; patients may receive red blood cell transfusions to obtain this level

Serum creatinine $\geq$ 1.5 $\times$ upper limit of normal (ULN)

International normalized ratio (INR) and activated partial thromboplastin time (aPTT) $\leq$ 1.5 $\times$ ULN

Serum AST and ALT $\leq$ 1.5 $\times$ ULN

Serum total bilirubin (TBILI) $\leq$ 1.0 $\times$ ULN (within normal limits), except for patients with Gilbert's syndrome, for whom direct bilirubin should be within the normal range

Serum alkaline phosphatase (ALK) $\leq$ 1.5 $\times$ ULN

Screening LVEF $\geq$ 50% on ECHO or MUGA and no decrease in LVEF by more than 15% absolute points from the pre-chemotherapy LVEF (pre-chemotherapy LVEF must be documented in the CRF)

LVEF assessment may be repeated once up to 3 weeks following the initial screening assessment to assess eligibility

14. Women of childbearing potential and men with partners of childbearing potential must be willing to use one highly effective form of nonhormonal contraception or 2 effective forms of nonhormonal contraception by the patient and/or partner and to continue its use for the duration of study treatment and for 6 months after the last dose of study treatment

    a) Acceptable forms of effective contraception should include 2 of the following:

        i. Placement of non-hormonal intrauterine device (IUD)

        ii. Condom with spermicidal foam/gel/film/cream/suppository

        iii. Diaphragm or cervical/vault caps with spermicidal foam/film/cream/suppository

    b) The above contraception is not a requirement in the case of any of the following:

        i. The male patient, or male partner of a female patient, is surgically sterilized.

        ii. The female patient is > 45 years of age and is postmenopausal (has not menstruated for at least 12 consecutive months)

        iii. The patient truly abstains from sexual activity and when this is the preferred and usual lifestyle of the patient.

    c) Contraception use should continue for the duration of the study treatment and for at least 6 months after the last dose of study treatment. Periodic abstinence (e.g., calendar ovulation, symptothermal, and post-ovulation methods) and withdrawal are not acceptable methods of contraception.

15. Negative serum pregnancy test for premenopausal women including women who have had a tubal ligation and for women less than 12 months after the onset of menopause

4.1.2 Exclusion Criteria

Patients who meet any of the following criteria will be excluded from study entry.

4.1.2.1 Disease-Related Exclusion Criteria

1. Stage IV (metastatic) breast cancer
2. History of any prior (ipsi- or contralateral) breast cancer except lobular CIS
3. Evidence of clinically evident gross residual or recurrent disease following preoperative therapy and surgery
4. PD during preoperative therapy
5. Treatment with any anti-cancer investigational drug within 28 days prior to commencing study treatment
6. History of other malignancy within the last 5 years except for appropriately treated CIS of the cervix, non-melanoma skin carcinoma, Stage I uterine cancer, or other non-breast malignancies with an outcome similar to those mentioned above
7. Patients for whom radiotherapy would be recommended for breast cancer treatment but for whom it is contraindicated because of medical reasons (e.g., connective tissue disorder or prior ipsilateral breast radiation)
8. Current NCI CTCAE (Version 4.0) Grade $\geq$ 2 peripheral neuropathy
9. History of exposure to the following cumulative doses of anthracyclines:

    Doxorubicin $>$ 240 mg/m^2

    Epirubicin $>$ 480 mg/m^2

    For other anthracyclines, exposure equivalent to doxorubicin $>$ 240 mg/m^2

10. Cardiopulmonary dysfunction as defined by any of the following:

    Significant symptoms (Grade $\geq$ 2) relating to LV dysfunction, cardiac arrhythmia, or cardiac ischemia while or since receiving preoperative therapy.

    Uncontrolled hypertension (systolic blood pressure $>$ 180 mmHg and/or diastolic blood pressure $>$ 100 mmHg)

    Inadequately controlled angina, serious cardiac arrhythmia not controlled by adequate medication, severe conduction abnormality, or clinically significant valvular disease

    Screening LVEF $<$ 50% by either ECHO or MUGA

    History of NCI CTCAE (Version 4.0) Grade $\geq$ 3 symptomatic CHF or NYHA criteria Class ≥ II

    History of a decrease in LVEF to $<$ 40% or symptomatic CHF with prior trastuzumab treatment (e.g., during preoperative therapy)

    Myocardial infarction within 12 months prior to randomization

    Requirement for continuous oxygen therapy

11. Prior treatment with trastuzumab emtansine

4.1.2.2 General Exclusion Criteria

12. Current severe, uncontrolled systemic disease (e.g., clinically significant cardiovascular, pulmonary, or metabolic disease; wound-healing disorders; ulcers)
13. For female patients, current pregnancy and/or lactation
14. Major surgical procedure unrelated to breast cancer or significant traumatic injury within approximately 28 days prior to randomization or anticipation of the need for major surgery during the course of study treatment
15. Any known liver disease, including known carriers of hepatitis B virus, hepatitis C, autoimmune hepatic disorders and sclerosing cholangitis
16. Concurrent, serious, uncontrolled infections or known infection with HIV
17. History of intolerance, including Grade 3 to 4 infusion reaction or hypersensitivity to trastuzumab or murine proteins
18. Active, unresolved infections at screening
19. Assessment by the investigator as being unable or unwilling to comply with the requirements of the protocol

4.2 METHOD OF TREATMENT ASSIGNMENT AND BLINDING

After written informed consent has been obtained and eligibility has been established and approved, the study site will obtain the patient randomization number and treatment assignment from the interactive voice response system/interactive web response system (IVRS/IWRS). Patients should receive their first dose of study treatment the day of randomization if possible, but no later than 5 business days after randomization. Patients will be randomized in a 1:1 ratio by a permuted block randomization scheme to one of the 2 treatment arms (trastuzumab or trastuzumab emtansine) through use of the IVRS/IWRS.

Randomization will be stratified by the stratification factors:

- Clinical stage at presentation: inoperable (Stage T4NxM0 or TxN2–3M0) versus operable (Stages T1-3N0 to 1M0)
- Hormone receptor status: ER or PR positive versus ER and PR negative/unknown
- Preoperative HER2-directed therapy: trastuzumab versus trastuzumab plus additional HER2-directed agent(s)
- Pathologic nodal status evaluated after preoperative therapy: node positive versus node negative/not done

4.3 STUDY TREATMENT

4.3.1 Formulation, Packaging, and Handling

For further details regarding the study drug, see the trastuzumab emtansine and trastuzumab Investigator's Brochure as well as local prescribing information for trastuzumab.

4.3.1.1 Trastuzumab Emtansine and Trastuzumab

4.3.1.1.1 Trastuzumab Emtansine

Trastuzumab emtansine is provided as a single-use lyophilized formulation in a colorless 20-mL Type I glass vial closed by means of a FluroTec-coated stopper and an overseal with flip-off cap. Upon receipt of trastuzumab emtansine, vials should be refrigerated at 2°C to 8°C (36°F to 46°F) until use. THE VIAL MUST NOT BE FROZEN OR SHAKEN. Trastuzumab emtansine must be stored in the original carton to protect it from light. Do not use the product beyond the expiration date provided by the manufacturer. The reconstituted product contains no preservative and is intended for single use only. Any remaining medication should be discarded.

All vials of trastuzumab emtansine should be handled by appropriately trained site staff wearing gloves and using appropriate procedures in place at the clinical site for preparation of chemotherapeutic drugs. Vials should be visually inspected upon receipt to ensure that they are intact without exterior contamination. Discard any cracked vials and report vials with surface contamination to the clinical site manager for assessment.

The lyophilized product should be reconstituted using Sterile Water for Injection (SWFI). Using a new syringe, 8 mL SWFI should be added to the vial and the vial swirled gently until the product is completely dissolved. The vial should not be shaken. The resulting product contains 20 mg/mL trastuzumab emtansine, 10 mM sodium succinate, pH 5.0, 60 mg/mL sucrose, and 0.02% (w/v) polysorbate 20. Each 20 mL vial contains enough trastuzumab emtansine to allow delivery of 160 mg trastuzumab emtansine. The reconstituted product contains no preservative and is intended for single use only.

The vial should be inspected to ensure the reconstituted product is a clear colorless solution, and is free of particulates before proceeding. Drug from any vial that appears abnormal upon inspection should not be administered to patients. Using a new syringe, the indicated volume of trastuzumab emtansine solution should be removed from the vial(s) and added to the IV bag containing at least 250 mL of 0.45% sodium chloride (preferred) or 0.9% sodium chloride injection and gently inverted to mix the solution. A 0.22 micron non-protein adsorptive polyethersulfone in-line filter is recommended when using 0.45% sodium chloride and required when using 0.9% sodium chloride injection. The solution of trastuzumab emtansine should not be shaken.

4.3.1.1.2 Trastuzumab

For further details, see the trastuzumab Investigator's Brochure as well as local prescribing information for trastuzumab.

4.3.2 <u>Dosage, Administration, and Compliance</u>

4.3.2.1 Trastuzumab Emtansine

Trastuzumab emtansine will be administered on Day 1 of a 3-week cycle q3w at a dose of 3.6 mg/kg IV. The total dose will be calculated based on the patient’s weight on Day 1

of (or up to 3 days before) each cycle with no upper limit. Changes in weight of $<10\%$ from baseline do not require dose recalculation.

Trastuzumab emtansine doses may be reduced to as low as 2.4 mg/kg, according to the dose-modification guidelines (see Table 2 in Section 5.1.3). Dose delays of up to 42 days from the last administered dose are permitted.

The first infusion of trastuzumab emtansine will be administered over 90 minutes ($\pm$10 minutes). Infusions may be slowed or interrupted for patients experiencing infusion-associated symptoms. Vital signs must be assessed before and after dose administration. Following the initial dose, patients will be observed for at least 60 minutes for fever, chills, or other infusion-associated symptoms. If prior infusions were well tolerated (without any signs or symptoms of infusion reactions), subsequent doses of trastuzumab emtansine may be administered over 30 minutes ($\pm$10 minutes), with a minimum 30-minute observation period after infusion. Local health authority guidelines must be followed with regard to further observation and monitoring, if applicable. Premedication for nausea and infusion reactions (e.g., acetaminophen or other analgesics, antihistamines such as diphenhydramine, or corticosteroids) may be given at the investigator's discretion.

4.3.2.2 Trastuzumab

Trastuzumab will be administered on Day 1 of a 3-week cycle at a maintenance dose of 6 mg/kg IV. A loading dose of 8 mg/kg is required if >6 weeks have elapsed since the prior dose of trastuzumab.

Infusion of trastuzumab should be performed in accordance with local guidelines and/or prescribing information.

4.3.3 Investigational Medicinal Product Accountability

All investigational medicinal products (IMPs) required for completion of this study will be provided by the Sponsor. Trastuzumab emtansine is the IMP in this study. Depending on local legislation, trastuzumab may also be considered an IMP in this study. Where permitted by regulatory requirements, sites will obtain and utilize commercially available trastuzumab.

The investigational site will acknowledge receipt of IMPs, using the IVRS/IWRS to confirm the shipment condition and content. Any damaged shipments will be replaced.

IMPs will either be disposed of at the study site according to the study site's institutional standard operating procedure or returned to the Sponsor with the appropriate documentation. The site's method of IMP destruction must be agreed upon by the Sponsor. The site must obtain written authorization from the Sponsor before any IMP is destroyed, and IMP destruction must be documented on the appropriate form.

Accurate records of all IMPs received at, dispensed from, returned to, and disposed of by the study site should be recorded on the Drug Inventory Log.

4.3.3.1 Post-Trial Access to Trastuzumab Emtansine

The Sponsor does not intend to provide trastuzumab emtansine or other study interventions to patients after conclusion of the study or any earlier patient withdrawal.

4.4 CONCOMITANT THERAPY AND FOOD

4.4.1 Permitted Therapy

Concomitant therapy and premedication are defined as non-IMPs.

Concomitant therapy includes any medication (e.g., prescription drugs, over-the-counter drugs, herbal/homeopathic remedies, nutritional supplements) or therapy used by a patient at any time from 7 days prior to randomization to the study completion/early termination visit.

All concomitant medications (within 7 days prior to randomization) and prior treatments for breast cancer must be reported in the electronic case report form (eCRF), including the following:

- All systemic therapies for breast cancer (drug name, dose, schedule, and duration), including chemotherapy, biologic therapy (antibody and small molecule therapies), or hormonal therapy (including ovarian ablation and drug induced ovarian suppression)
- Date and extent of primary surgery, as applicable
- Any locoregional radiation therapy (extent or volume and total dose)
- Bisphosphonate or denosumab therapy **(to be used in accordance with the approved labeled indication and/or nationally recognized treatment guidelines)**

All concomitant medications are to be reported until the end of study treatment visit. Thereafter, only medications and therapies applicable for long-term reporting must be reported, including the following:

- Breast cancer treatments (e.g., hormone therapy)
- Anticancer treatments for recurrence
- Bisphosphonate or denosumab therapy
- Medications related to the treatment of SAEs that are applicable for long-term reporting

Any medication that is necessary for the supportive management of the patient may be used at the discretion of the investigator.

4.4.1.1 Adjuvant Radiotherapy

Concomitant adjuvant radiotherapy includes the following:

- For patients undergoing breast-conserving surgery, whole breast irradiation is required. Primary tumor bed boost may be administered according to local policy. Regional node irradiation is required if the patient presented with clinical T3 or T4 disease and/or with clinical N2 or N3 disease and is recommended if there is residual disease in lymph nodes.
- For post-mastectomy patients, chest wall and regional node irradiation is required if the patient presented with clinical T3 or T4 disease and/or with clinical N2 or N3 disease and is recommended if there is residual disease in lymph nodes. For post-mastectomy patients who do not meet these criteria, radiotherapy is at the discretion of the investigator based on institutional standards.

When indicated, radiotherapy is to be given concurrently with study therapy. Radiotherapy should be administered within 60 days of surgery in the absence of complications requiring delay. Plans for reconstructive surgery should take into consideration the protocol therapy.

Dose fractionation of adjuvant whole breast, chest wall and regional node radiotherapy may be done according to local institutional guidelines.

4.4.1.2 Concomitant Hormonal Therapy

Concomitant hormonal therapy may be administered according to the following recommendations.

Female patients must be classified according to one of the following menopausal status definitions on the basis of their pre-chemotherapy status.

- Premenopausal

  $<$ 12 months since last menstrual period AND no prior bilateral ovariectomy AND not receiving estrogen replacement OR biochemical evidence of premenopausal status, according to local policies
- Postmenopausal

  $>$ 12 months since last menstrual period with no prior hysterectomy OR prior bilateral ovariectomy OR biochemical evidence of postmenopausal status, according to local policies

Female patients should be treated according to local guidelines. A minimum of 5 years of hormonal therapy should be planned.

NOTE: Endocrine therapy in male patients is to be given according to local guidelines.

Table 1 Recommendations for Hormonal Therapy

Clinical Scenario	Hormonal Therapy
Hormone receptor negative [a]	Not permitted
Hormone receptor positive [b] (premenopausal)	Tamoxifen for 5 years with or without ovarian suppression as per local policy
Hormone receptor positive [b] (postmenopausal)	Aromatase inhibitor for 5 years, OR<br><br>Aromatase inhibitor for 2-3 years, followed by tamoxifen to complete a total of 5 years, OR<br><br>Tamoxifen for 2-3 years, followed by aromatase inhibitor to complete a total of 5 years, OR<br><br>Tamoxifen for 5 years, OR<br><br>Tamoxifen for 5 years, followed by aromatase inhibitor for 5 years

ER = estrogen receptor; PR = progesterone receptor.

[a] Hormone receptor "positive" is defined as positive for ER and/or PR based on the preoperative or postoperative tumor pathology. The investigator may decide treatment policy according to local laboratory receptor status.

[b] Patients who are initially premenopausal may become postmenopausal over the course of the study, in which case hormonal therapy can be adjusted according to local policy.

4.4.2 <u>Prohibited Therapy</u>

Explicitly prohibited therapies prior to disease recurrence include the following:

- Anticancer therapies other than those administered in this study, including cytotoxic chemotherapy, radiotherapy (except for adjuvant radiotherapy for breast cancer after completion of chemotherapy), immunotherapy, and biological or targeted (e.g., lapatinib, neratinib) anticancer therapy
- Any investigational agent, except those used for this study

Potent CYP3A4 inhibitors, such as ketoconazole and erythromycin, should be avoided during the study treatment period with trastuzumab emtansine.

Excessive alcohol intake should be avoided (occasional to moderate use is permitted).

4.5 STUDY ASSESSMENTS

All patients will be closely monitored for safety and tolerability during all cycles of therapy. Patients should be assessed for toxicity prior to each dose; dosing will occur only if the clinical assessment and local laboratory test values are acceptable. Study treatment will be administered in 21-day cycles if no additional time is required for reversal of toxicity.

If the timing of a protocol-mandated procedure coincides with a holiday and/or weekend that precludes performance of the procedure within the allotted window, the procedure should be performed on the nearest following date. Study assessments are outlined in this section and in Appendix 1 and Appendix 2.

4.5.1 Description of Study Assessments

4.5.1.1 Medical History and Demographic Data

Medical history includes clinically significant diseases, surgeries, cancer history (including all prior cancer therapies and procedures), and all medications (e.g., prescription drugs, over-the-counter drugs, herbal/homeopathic remedies, nutritional supplements) used by the patient within 7 days prior to the screening visit. Demographic data will include age, sex, and self-reported race/ethnicity.

4.5.1.2 Vital Signs

Vital signs will include measurements of respiratory rate, pulse rate, and systolic and diastolic blood pressures while the patient is in a seated position, as well as weight and temperature.

4.5.1.3 Physical Examinations

A complete physical examination should include, at the minimum, the evaluation of bilateral breast, chest wall, and regional lymph nodes and of the cardiovascular, dermatological, musculoskeletal, respiratory, gastrointestinal (including an evaluation for the presence of hepatomegaly), and neurological systems. Any abnormality identified at baseline should be recorded on the General Medical History and Baseline Conditions eCRF.

Limited, symptom-directed physical examinations may be performed as indicated in the schedule of assessment. Changes from baseline abnormalities should be recorded in patient notes. New or worsened abnormalities should be recorded as AEs on the AE eCRF.

4.5.1.4 Radiologic Evaluations

Reports of most recent bilateral mammograms and/or MRI scans performed within 1 year prior to enrollment must be available. Mammograms of any remaining breast tissue should be performed at least annually during follow-up. Bone scan, CT, MRI, and/or PET-FDG scans may be performed as clinically indicated according to the investigator.

4.5.1.5 Laboratory Assessments

Samples for the following laboratory tests will be sent to the study site’s local laboratory for analysis:

– Hematology (hemoglobin, hematocrit, platelet count, WBC count, and absolute neutrophil count)

– Serum chemistry

  At baseline: sodium, potassium, chloride, bicarbonate, BUN or urea, creatinine, TBILI (and direct bilirubin when TBILI > ULN), albumin, ALT, AST, and ALK

  At each cycle: potassium, TBILI (and direct bilirubin when TBILI > ULN), ALT, AST, ALK, and other studies when clinically indicated

– Pregnancy test

  All women of childbearing potential (including those who have had a tubal ligation) will have a serum pregnancy test at screening. Urine pregnancy tests will be performed at specified subsequent visits. If a urine pregnancy test is positive, it must be confirmed by a serum pregnancy test.

– INR and aPTT at screening; otherwise, as clinically indicated

Samples for the following laboratory tests will be sent to one or several central laboratories or to Roche for analysis. Instruction manuals and supply kits will be provided for all central laboratory assessments.

– Prospective central testing for HER2-positive status by IHC and ISH

  A patient's HER2 status will be considered positive if the central laboratory reports Grade 3+ staining intensity (on a scale of 0 to 3+) by means of IHC analysis and/or ratio of $\geq$ 2.0 for the number of HER2 gene copies to the number of signals for CEP17. Paraffin-embedded tumor tissue block or a partial block must be obtained. If sites are unable to send a tissue block due to local regulations, at least 8 unstained slides should be sent for HER2 testing, and in addition up to 5 slides for exploratory biomarker research.

  Central laboratory confirmation of a positive HER2 status is required prior to randomization to the study. The outcome of this assessment will be communicated to the investigator

  After completion of HER2 testing for eligibility criteria applying prespecified HER2 tests, patient samples may also be tested with other HER2 assays to establish performance characteristics of these assays for diagnostic development. Testing could be performed on all screened patients (screen-failed and enrolled). These testing data will have no impact on eligibility and testing will be performed only after eligibility is established for each patient.

– Assessment of potential predictive candidate markers involving alterations to molecules relating to HER2 signaling; assessment of the mechanism of action of trastuzumab emtansine or trastuzumab, breast cancer biology, or both; assessment of the association of candidate markers with the safety profile of trastuzumab emtansine; or the improvement of diagnostic methods

  For these assessments, tumor blocks will be mandatorily obtained from the resection specimen after preoperative treatment and collected.

– Analysis of serum, plasma, and whole blood samples collected as part of the optional biomarker program

– Anti–trastuzumab emtansine antibody and anti-trastuzumab antibody
– Analysis of serum and plasma samples for trastuzumab emtansine and trastuzumab PK in approximately 50% of the patients in each treatment arm, respectively

4.5.1.6 Cardiac Assessments

4.5.1.6.1 Electrocardiograms

Single 12-lead ECGs will be collected locally and assessed at screening and otherwise as clinically indicated.

For safety monitoring purposes, any abnormalities on any of the ECGs will be documented on the eCRF. The investigator or designee must review, sign, and date all ECG tracings. Paper copies will be kept as part of the patient's permanent study file at the site. For ECG tracings that will fade over time (e.g., ECGs on thermal paper), lasting legible copies should be filed together with the original.

4.5.1.6.2 Left Ventricular Ejection Fraction

LVEF will be assessed by ECHO or MUGA. The same modality should be used throughout the study for each patient. Results of ECHO/MUGA performed prior to commencement and immediately after completion of preoperative therapy will also be collected in the eCRF.

4.5.1.7 Patient-Reported Outcomes

PRO data will be elicited from the patients in this study to more fully characterize the clinical profile of trastuzumab emtansine. The PRO instruments, translated as required into the local language, will be distributed by the investigational site staff and completed in their entirety by patients. To ensure instrument validity and that data standards meet health authority requirements, the PRO questionnaires (the EORTC QLQ-C30, the EORTC QLQ-BR23 breast cancer module, and the EuroQol EQ-5D) should be self-administered at the investigational site prior to the completion of other study assessments and the administration of study treatment at screening, Day 1 of Cycles 5 and 11, the study drug completion visit, and every 6 months during the follow-up period to Month 24 and then at Month 36.

The EORTC QLQ-C30 is a validated and reliable self-report measure (Aaronson et al. 1993; Sprangers et al. 1996; Fitzsimmons et al. 1999) that consists of 30 questions that assess five aspects of patient functioning (physical, emotional, role, cognitive, and social); three symptom scales (fatigue; nausea, vomiting, and pain; and the global health/quality of life) and six single items (dyspnea, insomnia, appetite loss, constipation, diarrhea, and financial difficulties). Scale scores can be obtained for the multi-item scales. The QLQ-BR23 breast cancer module is meant for use among patients varying in disease stage and treatment modality (Sprangers et al. 1996). The module comprises 23 questions assessing disease symptoms, side effects of treatment (surgery, chemotherapy, radiotherapy, and hormonal treatment), body image, sexual functioning, and future perspective. The breast cancer module incorporates five multiple-item scales

to assess systemic therapy side effects, arm symptoms, breast symptoms, body image, and sexual functioning. In addition, single items assess sexual enjoyment, hair loss, and future perspective. The QLQ-C30 and QLQ-BR23 take 10 to 15 minutes to complete (see Appendix 3 and Appendix 4, respectively).

The EuroQol EQ-5D questionnaire is a generic, preference-based health utility measure with questions about mobility, self-care, usual activities, pain/discomfort, and anxiety/depression that are used to build a composite of the patient's health status. The EQ-5D questionnaire will be utilized in this study for economic modeling (see Appendix 5).

4.5.1.8 Mandatory Samples for Determination of Patient Eligibility and Exploratory Biomarker Research

Tumor samples in the form of a formalin-fixed paraffin-embedded tumor block or partial block obtained from the pretreatment primary tumor (biopsy material) are required for study enrollment and must be submitted to the central pathology laboratory for assessment of HER2 status by IHC and ISH. If local regulations prevent the site from sending a (partial) biopsy block, a minimum of 8 slides should be sent for HER2 testing and additional 5 slides for exploratory biomarker analysis. If sufficient material from the pretreatment biopsy is not available for submission, the central pathology laboratory may use residual tumor tissue obtained at the time of definitive surgery to assess HER2 status.

If enough tissue (block, partial block, or slides) is available after eligibility HER2 testing, samples will also be used for exploratory biomarker research (see below). It is also mandatory to send a (partial) tissue block from the surgical specimen for exploratory biomarker research, as further outlined below. If sites are not able to send a tissue block due to local regulations, a minimum of 25 slides should be sent.

The pre-treatment biopsy specimen will be used for exploratory biomarker research to determine whether changes in biomarker profiles may characterize potential resistance mechanisms. Furthermore, both tissue samples collected (pre-treatment biopsy core and resection specimen) will be used to assess potential predictive candidate biomarkers involving alterations to molecules relating to HER2 signaling; to assess the mechanism of action of treatment, breast cancer biology, or both; to assess association of candidate biomarkers with safety profile of treatment; or to improve diagnostic methods. Examples of such markers are phosphatase and tensin homolog (PTEN), phosphoinositide 3-kinases (PI3K), and HER family receptors by protein or messenger RNA expression. Tissue may also be used for central testing of Ki67 and ER/PR staining. The final set of markers will be defined based on emerging data from the trastuzumab emtansine development program and/or literature data. Such biomarker research may methodically include assessment of protein expression (e.g., by IHC) and assessment of tumor DNA to explore non-inherited markers and assessment of tumor RNA. Remaining sample materials after the completion of the initial biomarker

assessments (e.g., aliquots of tumor RNA or DNA) may be used for re-testing, developing and validating diagnostic assays, or for further assessment of expanded marker panels. Figure 1 shows the tissue collection flow.

Figure 1 Tissue Collection Flow

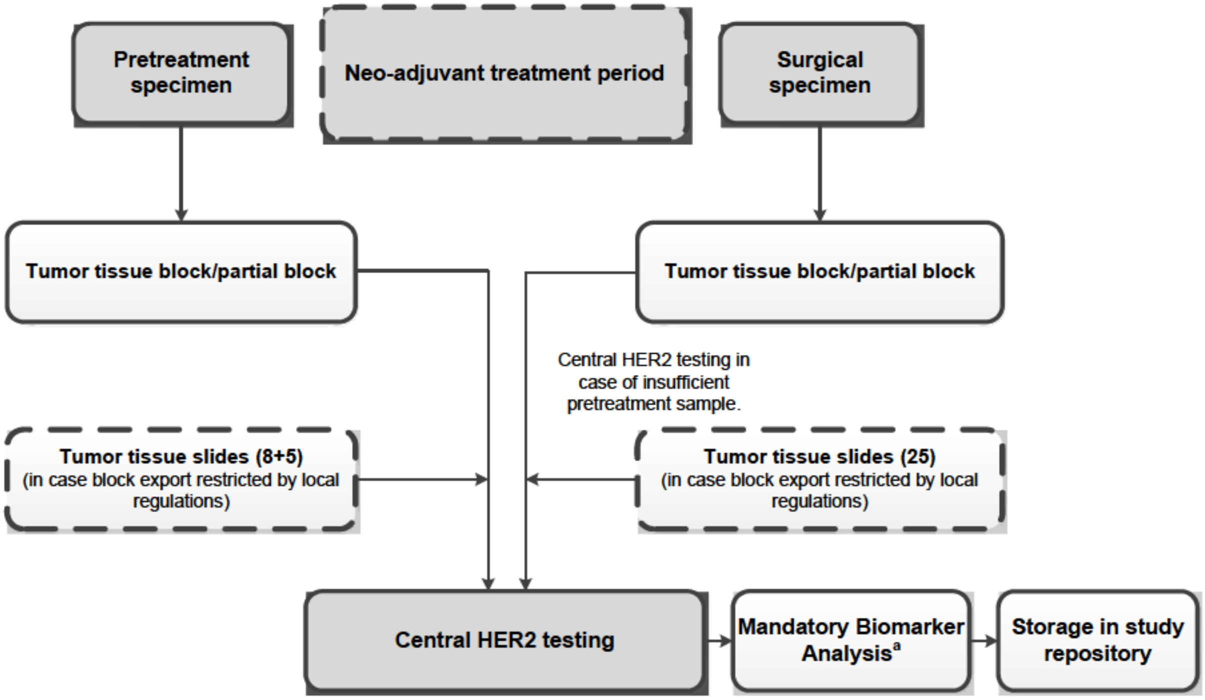

HER2 = human epidermal growth factor receptor 2.
[a] Biomarker analysis will only be performed on tissue from randomized patients.

After analyses, samples will be stored at a study's central biological samples repository for up to 15 years after the date of final closure of the associated clinical database, with the additional option of further long-term storage. The implementation and use of the study repository specimens is governed by the Study Steering Committee, with guidance from a dedicated translational advisory committee to ensure the appropriate use of the study specimens. All biomarker specimens will be retained for new research related to this study and/or disease in accordance with the recommendations and approval of the Study Steering Committee.

For sampling procedures, storage conditions, and shipment instructions, see the Sample Handling and Logistics Manual.

4.5.1.9 Optional Biomarker Research Samples

The blood samples collected will be used to identify biomarkers that may be predictive of response or toxicity to treatment and/or prognostic for breast cancer. Since the knowledge of new markers that may correlate with disease activity and the efficacy or safety of the treatment is evolving, the analytes may change during the course of the

study and may include determination of additional markers of tumorigenesis pathways and mechanisms of response to anti-HER2 therapies. The collected blood samples may also be used to develop and validate diagnostic assays and allow the generation of statistically meaningful biomarker data. Remaining sample materials after the completion of the initial biomarker assessments may be used for re-testing, developing and validating diagnostic assays, or for further assessment of expanded marker panels. Samples will be stored at a study's central biological samples repository for up to 15 years after database closure. For sampling procedures and shipment see instructions in the Sample Collection, Handling and Logistics Manual.

Specimens for biomarker research will be collected from patients who give specific consent to participate in this optional research. These optional biomarker specimens will be used to achieve the following objectives:

- To study the association of biomarkers with efficacy, AEs, or other effects associated with medicinal products
- To increase knowledge and understanding of disease biology
- To study drug response, including drug effects and the processes of drug absorption and disposition
- To develop biomarker or diagnostic assays and establish the performance characteristics of these assays

The specimens in the study repository will be made available for future biomarker research towards further understanding of treatment with trastuzumab emtansine or trastuzumab of breast cancer, related diseases and AEs, and for the development of potential associated diagnostic assays. Patients must consent to this optional program and long-term storage of their blood samples in this study repository. The implementation and use of the study repository specimens is governed by the Study Steering Committee, with guidance from a dedicated translational advisory committee to ensure the appropriate use of the study specimens. All biomarker specimens will be retained for new research related to this study and/or disease in accordance with the recommendations and approval of the Study Steering Committee.

Approval by the Institutional Review Board or Ethics Committee

Sampling for the Optional Biomarker Research is contingent upon the review and approval of the exploratory research and this portion of the Informed Consent Form by each site's IRB/EC and, if applicable, an appropriate regulatory body. If a site has not been granted approval for the optional biomarker research sampling, this section of the protocol will not be applicable at that site.

Sample Collection for Optional Biomarker Research

The following samples will be collected for identification of dynamic (non-inherited) biomarkers:

- Plasma samples

  For patients who consented to optional biomarker program, blood for plasma and serum isolation will be obtained at baseline, during treatment, at the end of treatment and at disease recurrence, as described in the schedule of assessments (Appendix 1).

  If patients are prematurely withdrawn from study treatment without recurrence, biomarker sample should be taken as well at the study drug completion visit and at disease recurrence.

The following sample will be collected for identification of genetic (inherited) biomarkers:

- Blood sample for genetic analysis

  For patients who consented to optional biomarker program, blood (approximately 6 mL in K3 EDTA) for DNA isolation will be collected at baseline as shown in the schedule of assessments. If, however, the genetic blood sample is not collected during the scheduled visit, it may be collected at any time (after randomization) during the conduct of the clinical study. The sample may be processed using techniques such as kinetic polymerase chain reaction (PCR) and DNA sequencing.

For all samples, dates of consent and specimen collection should be recorded on the associated biomarker page of the eCRF. For sampling procedures, storage conditions, and shipment instructions, see the Sample Handling and Logistics Manual.

The dynamic biomarker specimens will be subject to the confidentiality standards described Section 8.4. The genetic biomarker specimens will undergo additional processes to ensure confidentiality, as described below.

Confidentiality

Given the sensitive nature of genetic data, additional processes to ensure patient confidentiality for DNA biomarker specimens has been implemented. Upon receipt by the study biomarker repository, each specimen is "double-coded" by replacing the patient identification number with a new independent number. Data generated from the use of these specimens and all clinical data transferred from the clinical database and considered relevant are also labeled with this same independent number. A "linking key" between the patient identification number and this new independent number is stored in a secure database system. Access to the linking key is restricted to authorized individuals and is monitored by audit trail. Legitimate operational reasons for accessing the linking key are documented in a standard operating procedure.

Data generated from optional biomarker specimens must be available for inspection upon request by representatives of national and local health authorities, and study monitors, representatives, and collaborators, as appropriate.

Patient medical information associated with biomarker specimens is confidential and may only be disclosed to third parties as permitted by the Informed Consent Form (or separate authorization for use and disclosure of personal health information) signed by the patient, unless permitted or required by law.

Data derived from biomarker specimen analysis (except HER2 status) on individual patients will generally not be provided to study investigators.

Any inventions and resulting patents, improvements, and/or know-how originating from the use of the biomarker data will become and remain the exclusive and unburdened property of Roche, except where agreed otherwise.

Consent to Participate in the Optional Biomarker Research Program

The Informed Consent Form will contain a separate section that addresses participation in the optional biomarker program. The investigator or authorized designee will explain to each patient the objectives, methods, and potential hazards of participation in this program. Patients will be told that they are free to refuse to participate and may withdraw their specimens at any time and for any reason during the storage period. A separate, specific signature will be required to document a patient's agreement to provide optional biomarker specimens. Patients who decline to participate will check a “no” box in the appropriate section and will not provide a separate signature.

The investigator should document whether or not the patient has given consent to participate by completing the Optional Biomarker Research Sample Informed Consent eCRF.

In the event of a participant's death or loss of competence, the participant's specimens and data will continue to be used as part of the optional biomarker research.

A separate, specific signature is not required for the following mandatory samples:

- Tumor tissue collection for HER2 testing and biomarker analysis (pretreatment biopsy tissue sample and surgical tissue sample)

Withdrawal from the Optional Biomarker Research Program

Patients who give consent to provide optional biomarker specimens have the right to withdraw their specimens from the Optional Research Program at any time for any reason. If a patient wishes to withdraw consent to the testing of his or her specimens, the investigator must inform the Medical Monitor in writing of the patient's wishes using the Roche Clinical Repository (RCR) Subject Withdrawal Form and, if the trial is ongoing, must enter the date of withdrawal on the Optional Biomarker Research Sample

Withdrawal of Informed Consent eCRF. The patient will be provided with instructions on how to withdraw consent after the trial is closed. A patient's withdrawal from Study BO27938 does not, by itself, constitute withdrawal of specimens from the Optional Research Program. Likewise, a patient's withdrawal from the Optional Research Program does not constitute withdrawal from Study BO27938.

Monitoring and Oversight

Optional Biomarker Research specimens will be tracked in a manner consistent with Good Clinical Practice by a quality-controlled, auditable, and appropriately validated laboratory information management system, to ensure compliance with data confidentiality as well as adherence to authorized use of specimens as specified in this protocol and in the Informed Consent Form. Study monitors and auditors will have direct access to appropriate parts of records relating to patient participation in the Optional Biomarker Research program for the purposes of verifying the data provided to Sponsor. The site will permit monitoring, audits, IRB/EC review, and health authority inspections by providing direct access to source data and documents related to the optional biomarker samples.

4.5.2 Timing of Study Assessments

4.5.2.1 Screening and Pretreatment Assessments

Written informed consent for participation in the study must be obtained before performing any study-specific screening tests or evaluations. Informed Consent Forms for enrolled patients and for patients who are not subsequently enrolled will be maintained at the study site. Screening tests and evaluations will be performed within 30 days prior to enrollment unless otherwise specified. Results of standard-of-care tests or examinations performed prior to obtaining informed consent and within 30 days prior to enrollment may be used; such tests do not need to be repeated for screening. All screening evaluations must be completed and reviewed to confirm that patients meet all eligibility criteria before randomization. The investigator will maintain a screening log to record details of all patients screened and to confirm eligibility or record reasons for screening failure, as applicable.

Pretreatment tests and evaluations will be performed within 7 days prior to enrollment after confirmation of other eligibility criteria, unless otherwise specified.

Please see Appendix 1 for the schedule of screening and pretreatment assessments.

4.5.2.2 Assessments during Treatment

All patients will have a limited physical examination and be assessed for AEs and concomitant medications q3w for 14 cycles and will be assessed for disease recurrence every 3 months according to the schedule of assessments regardless of whether the patient completes or prematurely discontinues study therapy (trastuzumab emtansine or trastuzumab). Patients will be followed for disease-free status and survival according to

the schedule of assessments for approximately 10 years from the date of randomization of the last patient.

Scheduled study visits are based on a 21-day (3-week) cycle, with Cycle 1 beginning at Day 1. All visits must occur within ± 5 business days from the scheduled date, unless otherwise noted in the schedule of assessments. All assessments will be performed on the day of the specified visit unless a time window is specified. Assessments scheduled on the day of study treatment administration should be performed prior to study treatment administration unless otherwise noted. If the timing of a protocol-mandated procedure coincides with a holiday and/or weekend, it should be performed on the nearest following date.

Local laboratory assessments scheduled for Day 1 of all cycles must be performed within 72 hours prior to study treatment administration unless otherwise specified. Results of local laboratory assessments must be reviewed and the review documented prior to study treatment administration.

Please see Appendix 1 for the schedule of assessments performed during the treatment period.

4.5.2.3 Assessments at Study Treatment Completion/Early Termination Visit

Patients may remain on study treatment until disease recurrence as assessed by the investigator, unmanageable toxicity, completion of study treatment, or study termination by the Sponsor. Patients who discontinue study treatment will be asked to return to the clinic approximately 30 days (± 7 days) after the last study treatment administration for the study treatment discontinuation visit.

Please see Appendix 1 for the schedule of assessments performed at the study treatment completion/early termination visit.

4.5.2.4 Follow-Up Assessments

4.5.2.4.1 Scheduled Follow-up Assessments

After the study treatment completion/early termination visit, AEs should be followed as outlined in Section 5.5 and Section 5.6.

All patients must be followed up for 10 years, even if the assigned treatment is discontinued permanently.

The schedule of follow-up visits and tests for this study is the minimum required; investigators may see their patients more frequently according to their routine practice.

In cases of disease recurrence, diagnosed at any time during the study, patients will be out of the study schedule and will be followed up once a year (starting 1 year after first relapse) until Year 10 for survival, and new relapse events as per secondary endpoints.

Please see Appendix 1 for the schedule of follow-up assessments.

4.5.2.4.2 Follow-up and Confirmation of Disease Recurrence

The diagnosis of a breast cancer recurrence or second primary tumor should be confirmed histologically whenever possible. Some patients may have a suspicious recurrence that leads to death quite quickly without the possibility of confirming relapse of disease. Efforts should be made to obtain an autopsy report in such cases. The earliest date of diagnosis of recurrent disease should be used and recorded. This date should be based on objective clinical, radiological, histological or cytological evidence.

Recurrent disease includes local, regional, or distant recurrence and contralateral breast cancer. Patients who have a diagnosis of in situ breast disease or second (non-breast) malignancies should be maintained on a regular follow-up schedule wherever possible in order to fully capture any subsequent recurrent disease events.

The definitions of and procedures for confirming disease recurrence, death, and other noteworthy events on follow-up are given below:

a) Local invasive recurrence

Ipsilateral breast after previous lumpectomy

Defined as evidence of invasive tumor (except DCIS and LCIS) in the ipsilateral breast after lumpectomy. Patients who develop clinical evidence of tumor recurrence in the remainder of the ipsilateral breast should have a biopsy of the suspicious lesion to confirm the diagnosis.

Confirmed by positive histology or cytology

Ipsilateral after previous mastectomy

Defined as evidence of invasive tumor in any soft tissue or skin of the ipsilateral chest wall. This includes the area bounded by the midline of the sternum, extending superiorly to the clavicle, and inferiorly to the costal margin. Soft tissue recurrences in this area extending into the bony chest wall or across the midline will be considered as evidence of local recurrence.

Confirmed by positive histology or cytology

b) Regional recurrence

Defined as the development of tumor in the ipsilateral internal mammary lymph nodes, ipsilateral axillary lymph nodes or supraclavicular lymph nodes as well as extranodal soft tissue of the ipsilateral axilla. Regional recurrence does not include tumor in the opposite breast.

Confirmed by positive histology or cytology, or radiologic evidence (especially in case of PET activity or visible internal mammary lymph nodes on CT or MRI if no biopsy was performed)

c) Distant recurrence

Defined as evidence of tumor in all areas, with the exception of those described in a) and b) above

Confirmed by the following criteria:

– Skin, subcutaneous tissue, and lymph nodes (other than local or regional)

  Positive cytology, aspirate or biopsy, OR

  Radiological (CT scan, MRI, PET, or ultrasound) evidence of metastatic disease

– Bone

  X-ray, CT scan, or MRI evidence of lytic or blastic lesions consistent with bone metastasis, OR

  Bone scan (requires additional radiological investigation, alone not acceptable in case of diagnostic doubt), OR

  Biopsy proof of bone metastases or cytology

– Bone marrow

  Positive cytology or histology or MRI scan

– Lung

  Radiologic evidence of multiple pulmonary nodules consistent with pulmonary metastases

  Positive cytology or histology (practically rarely performed with the exception of solitary nodules)

  NOTE: For solitary lung lesions, cytological or histological confirmation should be obtained in case of diagnostic doubt. Proof of neoplastic pleural effusions should be established by cytology or pleural biopsy.

– Liver

  Radiologic evidence consistent with liver metastases, OR

  Liver biopsy or fine needle aspiration

  NOTE: If radiological findings are not definitive (especially with solitary liver nodules), a liver biopsy is recommended; however, if a biopsy is not performed, serial scans should be obtained if possible to document stability or progression.

– Central nervous system

  Positive MRI or CT scan, usually in a patient with neurologic symptoms, OR

  Biopsy or cytology (e.g., for a diagnosis of meningeal involvement). However, meningeal involvement may also be diagnosed by CT scan

or MRI and depending from the general status of the patient additional investigations (including cytology of the cerebrospinal fluid).

d) Contralateral invasive breast cancer

Confirmed by positive cytology or histology

e) Second primary malignancy (breast or other cancer)

Any positive diagnosis of a second (non-breast) primary cancer other than basal or squamous cell carcinoma of the skin, or CIS of the cervix will be considered an event in the analysis of the IDFS including second primary non-breast cancer endpoint; however, they will not be included in the IDFS primary endpoint.

LCIS and DCIS of the breast and myelodysplastic syndrome are not considered progression events. The diagnosis of a second primary cancer must be confirmed histologically.

All second primary malignancies are to be reported whenever they occur during the study.

NOTE: Patients diagnosed with a second primary malignancy not requiring systemic therapy (i.e., chemotherapy, hormonal therapy, targeted therapy, etc) and with no evidence of breast cancer recurrence will remain on study and should continue with study drug treatment according to the protocol and schedule of assessment, if considered by the investigator to be in the patient's best interest, whenever possible.

f) Death without recurrence

Any death occurring without prior breast cancer recurrence or second (non-breast) malignancy is considered an event for the following endpoints: IDFS including second primary non-breast cancer, DFS, and OS.

g) Other noteworthy events

The following events should be recorded on the follow-up eCRF:

Ipsilateral and contralateral LCIS

Ipsilateral and contralateral DCIS

NOTE: These events are not considered recurrent disease, but must be recorded.

4.6 PATIENT, STUDY, AND SITE DISCONTINUATION

4.6.1 Patient Discontinuation

The investigator has the right to discontinue a patient from study drug or withdraw a patient from the study at any time. In addition, patients have the right to voluntarily discontinue study drug or withdraw from the study at any time for any reason. Reasons for discontinuation of study drug or withdrawal from the study may include, but are not limited to, the following:

- Patient withdrawal of consent at any time
- Any medical condition that the investigator or Sponsor determines may jeopardize the patient's safety if he or she continues in the study
- Investigator or Sponsor determines it is in the best interest of the patient
- Completion of all assessments including 10 years of follow-up
- The study is closed by the Sponsor

4.6.1.1 Discontinuation from Study Drug

Patients must discontinue study drug if they experience any of the following:

- Pregnancy
- Disease recurrence
- Symptomatic CHF at any point in the study
- Two consecutive or three intermittent dose delays due to asymptomatic decrease in LVEF
- Inability to receive trastuzumab emtansine after 2 dose reductions of trastuzumab emtansine due to toxicity
- Hold on drug administration for $>$ 42 days from last dose due to toxicity
- Unacceptable toxicity
- Intercurrent, non−cancer-related illness that prevents continuation of protocol therapy or follow-up
- Major protocol violation that may jeopardize the patient's safety according to the Sponsor
- Repeated patient noncompliance with protocol requirements
- Changes in the patient's condition or study drug−related toxicity such that in the opinion of the investigator continued participation in the protocol would compromise the patient's well-being
- Withdrawal of patient consent

Patients who discontinue study drug prematurely will be asked to return to the clinic for a study treatment completion/early termination visit (see Section 4.5.2.3) and may undergo

follow-up assessments (see Section 4.5.2.4). The primary reason for premature study drug discontinuation should be documented on the appropriate eCRF. Patients who discontinue study drug prematurely will not be replaced.

Patients who discontinue trastuzumab emtansine prior to completion of 14 cycles of study therapy may continue treatment with trastuzumab so as to complete 14 cycles of HER2-directed study treatment. For example, if a patient receives 7 cycles of trastuzumab emtansine, then the patient could, if otherwise clinically appropriate according to the protocol safety plan and investigator judgment, receive up to 7 cycles of trastuzumab. Patients who discontinue trastuzumab emtansine because of toxicity that may be attributed to the trastuzumab component (e.g., hypersensitivity, cardiac toxicity, pneumonitis) may not continue to receive trastuzumab after discontinuation of trastuzumab emtansine.

4.6.1.2 Withdrawal from Study

Every effort should be made to obtain information on patients who withdraw from the study. The primary reason for withdrawal from the study should be documented on the appropriate eCRF. Patients will not be followed for any reason after consent has been withdrawn. Patients who withdraw from the study will not be replaced.

Withdrawal from entire study

Should a patient decide to withdraw from the study, all efforts will be made to complete and report the observations for that patient as thoroughly as possible. No further data will be collected after the date of the patient's withdrawal from study. The investigator should contact the patient or a legally authorized relative by telephone or through a personal visit to establish as completely as possible the reason for the withdrawal. A complete final evaluation at the time of the patient's withdrawal should be made, along with an explanation of why the patient is withdrawing from the study.

Partial withdrawal from study

All of the above provisions regarding withdrawal from the entire study are applicable to partial withdrawal from the study, except that the patient must agree to be contacted for further information on recurrence as per the primary study endpoint and survival status. Whenever possible, information on recurrence should be documented through review of medical records as well as patient contact. It should be documented in both the medical records and the eCRF that the patient agreed to be contacted for information on survival despite the patient's withdrawal of informed consent.

In the case of patients who do not show up for scheduled visits, site staff should make several attempts (i.e., at least three attempts within a reasonable period of time after a missed visit) to contact these patients for follow-up information. The collection of follow-up data is extremely important for the reliable estimation of study endpoints.

If a patient is lost to follow-up, contact will initially be attempted through the trial research nurse and the lead investigator at each study site. If these attempts are unsuccessful, the patient's physician will be contacted and asked to contact the patient or the patient's family and provide follow-up information to the recruiting study site.

Only after sufficient unsuccessful attempts at contact have been made may a patient be declared "lost to follow-up."

4.6.2 Study and Site Discontinuation

The Sponsor has the right to terminate this study at any time. Reasons for terminating the study may include, but are not limited to, the following:

- The incidence or severity of AEs in this or other studies indicates a potential health hazard to patients.
- Patient enrollment is unsatisfactory.

The Sponsor will notify the investigator if the study is placed on hold, or if the Sponsor decides to discontinue the study or development program.

The Sponsor has the right to replace a site at any time. Reasons for replacing a site may include, but are not limited to, the following:

- Excessively slow recruitment
- Poor protocol adherence
- Inaccurate or incomplete data recording
- Non-compliance with the International Conference on Harmonisation (ICH) guideline for Good Clinical Practice

5. ASSESSMENT OF SAFETY

5.1 SAFETY PLAN

5.1.1 Toxicities Associated with Trastuzumab Emtansine

The safety plan for patients in this study is based on the known nonclinical toxicities of trastuzumab emtansine, clinical experience with this molecule in completed and ongoing studies, and clinical toxicities related to its components (trastuzumab and maytansine, the parent drug of DM1). Thrombocytopenia, increases in serum AST and ALT, infusion/hypersensitivity reaction, pneumonitis, and nodular regenerative hyperplasia (NRH) have been identified as risks associated with trastuzumab emtansine use. NRH is a rare liver condition characterized by widespread benign transformation of hepatic parenchyma into small regenerative nodules, and diagnosis can only be confirmed by histopathology. The anticipated safety risks and potential safety risks of trastuzumab emtansine are further detailed in the IB. Please refer to the Investigator Brochure for a

complete summary of safety. Risk management guidance to avoid or minimize such anticipated toxicities, is detailed herein (Table 2) as well as in the IB.

The iDMC will meet to review AEs on a regularly scheduled basis

5.1.2 Toxicities Associated with Trastuzumab

The anticipated safety risks and potential safety risks of trastuzumab are detailed in the IB and local prescribing guidelines. Please refer to the Investigator Brochure for a complete summary of safety.

5.1.3 Management of Specific Adverse Events

Guidelines for managing specific AEs are provided in Table 3. For AEs not listed in Table 3, the following guidance should be used: for Grade 3 non-hematologic AEs not adequately managed by standard medical intervention or for any Grade 4 non-hematologic AE, study treatment should be held until recovery to Grade ≤ 1. A maximum dose delay of 42 days from the last administered dose of study medication will be allowed for recovery. After appropriate recovery, trastuzumab emtansine may be resumed with one dose level reduction (e.g., trastuzumab emtansine reduced from 3.6 mg/kg to 3 mg/kg or from 3 mg/kg to 2.4 mg/kg). Dose reduction levels for trastuzumab emtansine are shown in Table 2. For patients who have an event while being treated with trastuzumab emtansine 2.4 mg/kg, study treatment will be discontinued. The dose of trastuzumab emtansine, once reduced, may not be re-escalated. There are no dose reductions for control arm therapy (trastuzumab).

Patients who discontinue trastuzumab emtansine may complete the duration of their intended study treatment up to 14 cycles of HER2-directed therapy with trastuzumab if appropriate based on toxicity considerations and investigator discretion. Patients who discontinue trastuzumab emtansine for cardiac toxicity, or other toxicity that may be attributed to the trastuzumab component (e.g., hypersensitivity, pneumonitis) may not continue on trastuzumab after discontinuation of trastuzumab emtansine.

Table 2 Dose Reduction for Trastuzumab Emtansine

Dose Level	Dose
0	3.6 mg/kg
-1	3.0 mg/kg
-2	2.4 mg/kg
Indication for further dose reduction	Off study treatment

Table 3 Guidelines for Managing Specific Adverse Events

Event	Action to Be Taken
Infusion reactions	
Infusion-related dyspnea or clinically significant hypotension, Grade ≥ 3 allergic reaction	Stop infusion, study treatment permanently discontinued.<br>Supportive care with oxygen, β-agonists, antihistamines, antipyretics, or corticosteroids may be used, as appropriate, at the investigator's discretion.<br>Patients should be monitored until complete resolution of symptoms.
Infusion-related symptoms (e.g., chills, fever)	Decrease infusion rate by 50% or interrupt infusion for patients who experience any other infusion-related symptoms (e.g., chills, fever).<br>When symptoms have completely resolved, infusion may be restarted at ≤ 50% of prior rate and increased in 50% increments every 30 minutes as tolerated. Infusions may be restarted at the full rate at the next cycle, with appropriate monitoring.<br>Supportive care with oxygen, β-agonists, antihistamines, antipyretics, or corticosteroids may be used as appropriate at the investigator's discretion.<br>Premedication with corticosteroids, antihistamines, and antipyretics may be used before subsequent infusions at the investigator's discretion.<br>Patients should be monitored until complete resolution of symptoms.
Hematologic toxicity	
Grade ≥ 3 hematologic toxicity (other than thrombocytopenia)	Withhold study treatment until recovery to ≤ Grade 1. Weekly CBC assessments should be done until recovery, as medically indicated.<br>A maximum dose delay of 42 days from the last administered dose to Grade ≤ 1 or baseline will be allowed; otherwise, patients must be discontinued from study treatment.
Grade 2 or 3 thrombocytopenia on day of scheduled treatment	Assess platelet counts weekly or as medically indicated until recovery. Hold study treatment until Grade≤ 1. Resume treatment without dose reduction. If a patient requires 2 delays due to thrombocytopenia, consider reducing dose by one level.
Grade 4 thrombocytopenia at any time	Assess platelet counts weekly or as medically indicated until recovery. Hold trastuzumab emtansine until Grade ≤ 1, then resume with one dose level reduction (i.e., from 3.6 mg/kg to 3 mg/kg or from 3 mg/kg to 2.4 mg/kg) in subsequent cycles. If event occurs with 2.4 mg/kg dose, discontinue study treatment.

Table 3 Guidelines for Managing Specific Adverse Events (cont.)

Event	Action to Be Taken
Hepatotoxicity	
ALT	For a Grade 2-3 ALT that occurs on the laboratory evaluation for cycle Day 1 or the planned day of dosing, hold trastuzumab emtansine until ALT recovers to ≤ Grade 1. Resume with dose reduction by one level for Grade 2 or 3 elevations. Grade 2-3 ALT elevations that are noted between cycles do not require dose delay or reduction unless ALT remains elevated (≥ Grade 2) at the time of planned dosing.<br><br>For Grade 4 ALT (confirmed by a repeat lab evaluation), discontinue trastuzumab emtansine.
AST	For Grade 2 AST on the laboratory evaluation for cycle Day 1 or the planned day of dosing, hold trastuzumab emtansine until AST recovers to ≤ Grade 1. Resume without dose reduction when recovered.<br><br>For Grade 3 or 4 AST on the laboratory evaluation for cycle Day 1 or the planned day of dosing, hold trastuzumab emtansine until AST recovers to ≤ Grade 1. Resume with dose reduction by one level when recovered.
TBILI	For TBILI > 1.0 × ULN to ≤ 2.0 × ULN that occurs on the laboratory evaluation for cycle Day 1 or the day of planned dosing, hold trastuzumab emtansine until TBILI recovers to ≤ 1.0 × ULN (or direct bilirubin recovers to ≥ 1.0 × ULN for patients with Gilbert's syndrome). For TBILI elevations > 1.0 × ULN to ≤ 2.0 × ULN, resume when recovered with a one level dose reduction.<br><br>For TBILI > 2 × ULN at any time (or direct bilirubin > 2 × ULN for Gilbert's syndrome), discontinue trastuzumab emtansine and report the event as an SAE (if applicable) or non-serious expedited AE (if applicable).<br><br>Assess AST, ALT, and TBILI weekly or as medically indicated until recovery. Allow a maximum dose delay of 42 days from the last administered dose to recovery as described above or otherwise discontinue study treatment.<br><br>For any clinical signs of liver dysfunction, discontinue trastuzumab emtansine and have the patient evaluated by a hepatologist. If there are signs of portal hypertension (e.g., ascites and varices), the possibility of NRH should be considered. Trastuzumab emtansine should be discontinued in the event of a diagnosis of NRH.

Table 3 Guidelines for Managing Specific Adverse Events (cont.)

Event	Action to Be Taken
Neurotoxicity	
Grade ≥ 3 peripheral neuropathy	Discontinue trastuzumab emtansine if event does not resolve to Grade ≤ 2 or baseline value within 42 days after the last administered dose.
Cardiotoxicity	
LVSD	Refer to Figure 2 for the algorithm for continuation and discontinuation of study treatment on the basis of asymptomatic LVEF assessment.
Grade 3–4 LVSD or Grade 3–4 heart failure	Discontinue study treatment and report as an SAE.
Grade 2 heart failure accompanied by LVEF < 45%	Discontinue study treatment and report as an SAE.-
Radiotherapy-related toxicity Grade ≥ 2 (for skin toxicity, Grade 3 or higher)	Report as SAE (if applicable). Hold study treatment until recovery to Grade ≤ 1.

AE = adverse event; CBC = complete blood count; LVEF = left ventricular ejection fraction; LVSD = left ventricular systolic dysfunction; NRH = nodular regenerative hyperplasia; SAE = serious adverse event; ULN = upper limit of normal.

Figure 2 Algorithm for Continuation and Discontinuation of Study Treatment Based on LVEF Assessment

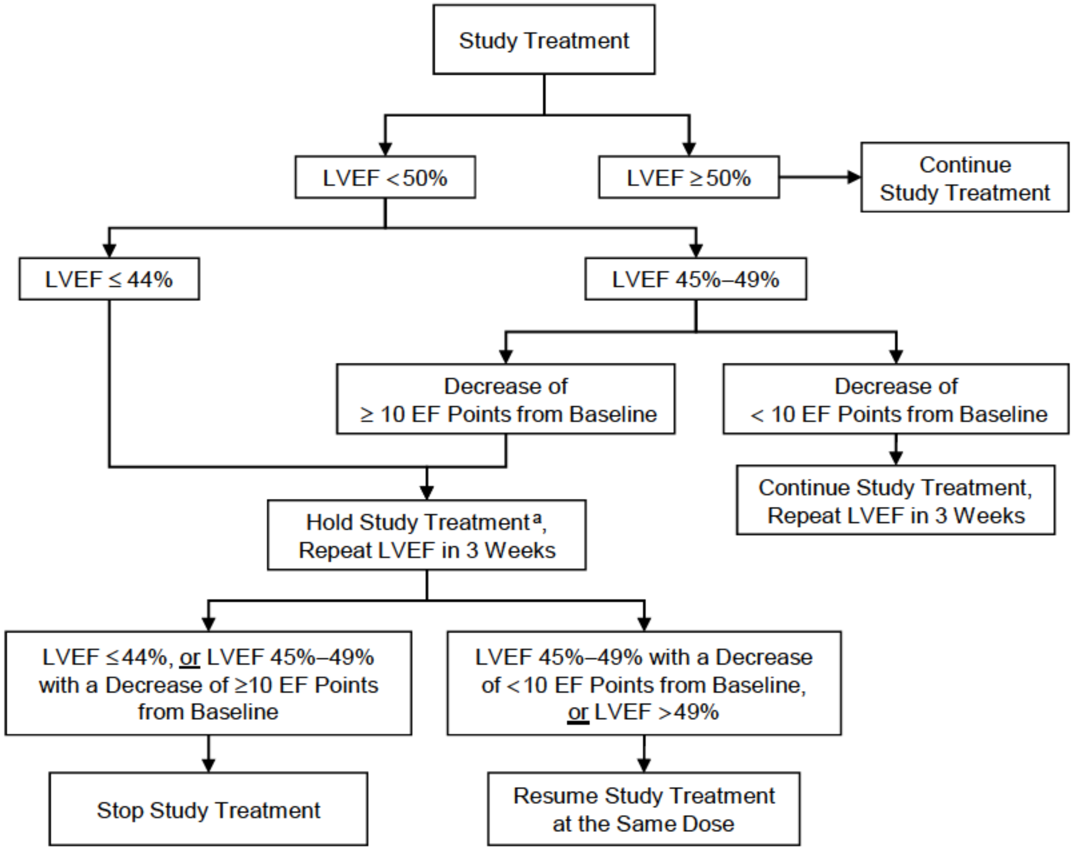

LVEF = left ventricular; EF = ejection fraction.

[a] Three intermittent holds of study treatment will lead to discontinuation.

Baseline refers to the pre-chemotherapy LVEF value, not the screening LVEF.

5.2 SAFETY PARAMETERS AND DEFINITIONS

Safety assessments will consist of monitoring and recording AEs, including SAEs and non-serious AEs of special interest; measurement of protocol-specified safety laboratory assessments; measurement of protocol-specified vital signs; and other protocol-specified tests that are deemed critical to the safety evaluation of the study.

Certain types of events require immediate reporting to the Sponsor, as outlined in Section 5.4.

5.2.1 Adverse Events

According to the ICH guideline for Good Clinical Practice, an AE is any untoward medical occurrence in a clinical investigation subject administered a pharmaceutical product, regardless of causal attribution. An AE can therefore be any of the following:

- Any unfavorable and unintended sign (including an abnormal laboratory finding), symptom, or disease temporally associated with the use of a medicinal product, whether or not considered related to the medicinal product
- Any new disease or exacerbation of an existing disease (a worsening in the character, frequency, or severity of a known condition), except as described in Section 5.3.5.10
- Recurrence of an intermittent medical condition (e.g., headache) not present at baseline
- Any deterioration in a laboratory value or other clinical test (e.g., ECG, X-ray) that is associated with symptoms or leads to a change in study treatment or concomitant treatment or discontinuation from study drug
- AEs that are related to a protocol-mandated intervention, including those that occur prior to assignment of study treatment (e.g., screening invasive procedures such as biopsies)

5.2.2 Serious Adverse Events (Immediately Reportable to the Sponsor)

A serious adverse event (SAE) is any AE that meets any of the following criteria:

- Fatal (i.e., the AE actually causes or leads to death)
- Life threatening (i.e., the AE, in the view of the investigator, places the patient at immediate risk of death)

    This does not include any AE that had it occurred in a more severe form or was allowed to continue might have caused death.

- Requires or prolongs inpatient hospitalization (see Section 5.3.5.11)
- Results in persistent or significant disability/incapacity (i.e., the AE results in substantial disruption of the patient's ability to conduct normal life functions)

- Congenital anomaly/birth defect in a neonate/infant born to a mother exposed to study drug
- Significant medical event in the investigator's judgment (e.g., may jeopardize the patient or may require medical/surgical intervention to prevent one of the outcomes listed above)

The terms "severe" and "serious" are not synonymous. Severity refers to the intensity of an AE (rated as mild, moderate, or severe, or according to NCI CTCAE criteria; see Section 5.3.3); the event itself may be of relatively minor medical significance (such as severe headache without any further findings).

Severity and seriousness need to be independently assessed for each AE recorded on the eCRF.

Serious AEs are required to be reported by the investigator to the Sponsor within 24 hours after learning of the event (see Section 5.4.2 for reporting instructions).

5.2.3 Non-Serious Adverse Events of Special Interest (Immediately Reportable to the Sponsor)

Non-serious AEs of special interest are required to be reported by the investigator to the Sponsor within 24 hours after learning of the event (see Section 5.4.2 for reporting instructions). AEs of special interest for this study include the following:

- Cases of increased serum ALT or AST in combination with either an increased serum TBILI or clinical jaundice, as defined in Section 5.3.5.6

5.3 METHODS AND TIMING FOR CAPTURING AND ASSESSING SAFETY PARAMETERS

The investigator is responsible for ensuring that all AEs (see Section 5.2.1 for definition) are recorded on the AE eCRF and reported to the Sponsor in accordance with instructions provided in this section and in Section 5.4 through Section 5.6.

For each AE recorded on the AE eCRF, the investigator will make an assessment of seriousness (see Section 5.2.2 for seriousness criteria), severity (see Section 5.3.3), and causality (see Section 5.3.4).

5.3.1 Adverse Event Reporting Period

Investigators will seek information on AEs at each patient contact. All AEs, whether reported by the patient or noted by study personnel, will be recorded in the patient's medical record and on the AE eCRF.

After informed consent has been obtained **but prior to initiation of study drug**, only SAEs caused by a protocol-mandated intervention should be reported (e.g., SAEs related to invasive procedures such as biopsies).

After initiation of study drug, all AEs, regardless of relationship to study drug, will be reported until 30 days after the last dose of study drug. After this period, investigators should report any deaths, SAEs, or other AEs of concern that are believed to be related to prior treatment with study drug (see Section 5.6).

5.3.2 Eliciting Adverse Event Information

A consistent methodology of non-directive questioning should be adopted for eliciting AE information at all patient evaluation time points. Examples of non-directive questions include the following:

"How have you felt since your last clinic visit?"

"Have you had any new or changed health problems since you were last here?"

5.3.3 Assessment of Severity of Adverse Events

The AE severity grading scale for the NCI CTCAE Version 4.0 will be used for assessing AE severity. Table 4 will be used for assessing severity for AEs that are not specifically listed in the NCI CTCAE.

Table 4 Adverse Event Severity Grading Scale

Grade	Severity
1	Mild; asymptomatic or mild symptoms; clinical or diagnostic observations only; or intervention not indicated
2	Moderate; minimal, local, or non-invasive intervention indicated; or limiting age-appropriate instrumental activities of daily living [a]
3	Severe or medically significant, but not immediately life-threatening; hospitalization or prolongation of hospitalization indicated; disabling; or limiting self-care activities of daily living [b,c]
4	Life-threatening consequences or urgent intervention indicated [d]
5	Death related to adverse event [d]

NCI CTCAE = National Cancer Institute Common Terminology Criteria for Adverse Events.

Note: Based on the NCI CTCAE, Version 4.0, which can be found at: http://evs.nci.nih.gov/ftp1/CTCAE/CTCAE_4.03_2010-06-14_QuickReference_8.5x11.pdf

[a] Instrumental activities of daily living refer to preparing meals, shopping for groceries or clothes, using the telephone, managing money, etc.

[b] Examples of self-care activities of daily living include bathing, dressing and undressing, feeding one's self, using the toilet, and taking medications, as performed by patients who are not bedridden.

[c] If an event is assessed as a "significant medical event," it must be reported as an SAE (see Section 5.4.2 for reporting instructions), per the definition of SAE in Section 5.2.2.

[d] Grade 4 and 5 events must be reported as SAEs (see Section 5.4.2 for reporting instructions), per the definition of SAE in Section 5.2.2.

5.3.4 Assessment of Causality of Adverse Events

Investigators should use their knowledge of the patient, the circumstances surrounding the event, and an evaluation of any potential alternative causes to determine whether or not an AE is considered to be related to the study drug, indicating "yes" or "no" accordingly. The following guidance should be taken into consideration:

- Temporal relationship of event onset to the initiation of study drug
- Course of the event, considering especially the effects of dose reduction, discontinuation of study drug, or reintroduction of study drug (where applicable)
- Known association of the event with the study drug or with similar treatments
- Known association of the event with the disease under study
- Presence of risk factors in the patient or use of concomitant medications known to increase the occurrence of the event
- Presence of non-treatment-related factors that are known to be associated with the occurrence of the event

For patients receiving combination therapy, causality will be assessed individually for each protocol-mandated therapy.

5.3.5 Procedures for Recording Adverse Events

Investigators should use correct medical terminology/concepts when recording AEs on the AE eCRF. Avoid colloquialisms and abbreviations.

Only one AE term should be recorded in the event field on the AE eCRF.

5.3.5.1 Diagnosis versus Signs and Symptoms

A diagnosis (if known) should be recorded on the AE eCRF rather than individual signs and symptoms (e.g., record only liver failure or hepatitis rather than jaundice, asterixis, and elevated transaminases). However, if a constellation of signs and/or symptoms cannot be medically characterized as a single diagnosis or syndrome at the time of reporting, each individual event should be recorded on the AE eCRF. If a diagnosis is subsequently established, all previously reported AEs based on signs and symptoms should be nullified and replaced by one AE report based on the single diagnosis, with a starting date that corresponds to the starting date of the first symptom of the eventual diagnosis.

5.3.5.2 AEs Occurring Secondary to Other Events

In general, AEs occurring secondary to other events (e.g., cascade events or clinical sequelae) should be identified by their primary cause, with the exception of severe or serious secondary events. However, medically significant AEs occurring secondary to an initiating event that are separated in time should be recorded as independent events on the AE eCRF. For example:

- If vomiting results in mild dehydration with no additional treatment in a healthy adult, only vomiting should be reported on the eCRF.
- If vomiting results in severe dehydration, both events should be reported separately on the eCRF.
- If a severe gastrointestinal hemorrhage leads to renal failure, both events should be reported separately on the eCRF.
- If dizziness leads to a fall and subsequent fracture, all three events should be reported separately on the eCRF.
- If neutropenia is accompanied by a mild, non-serious infection, only neutropenia should be reported on the eCRF.
- If neutropenia is accompanied by a severe or serious infection, both events should be reported separately on the eCRF.

All AEs should be recorded separately on the AE eCRF if it is unclear as to whether the events are associated.

5.3.5.3 Persistent or Recurrent Adverse Events

A persistent AE is one that extends continuously, without resolution, between patient evaluation time points. Such events should only be recorded once on the AE eCRF. The initial severity of the event should be recorded, and the severity should be updated to reflect the most extreme severity any time the event worsens. If the event becomes serious, the AE eCRF should be updated to reflect this.

A recurrent AE is one that resolves between patient evaluation time points and subsequently recurs. Each recurrence of an AE should be recorded separately on the AE eCRF.

5.3.5.4 Abnormal Laboratory Values

Not every laboratory abnormality qualifies as an AE. A laboratory test result should be reported as an AE if it meets any of the following criteria:

- Accompanied by clinical symptoms
- Results in a change in study treatment (e.g., dosage modification, treatment interruption, or treatment discontinuation)
- Results in a medical intervention (e.g., potassium supplementation for hypokalemia) or a change in concomitant therapy

- Clinically significant in the investigator's judgment

It is the investigator's responsibility to review all laboratory findings. Medical and scientific judgment should be exercised in deciding whether an isolated laboratory abnormality should be classified as an AE.

If a clinically significant laboratory abnormality is a sign of a disease or syndrome (e.g., ALK and bilirubin 5 times the ULN associated with cholecystitis), only the diagnosis (i.e., cholecystitis) should be recorded on the AE eCRF.

If a clinically significant laboratory abnormality is not a sign of a disease or syndrome, the abnormality itself should be recorded on the AE eCRF, along with a descriptor indicating if the test result is above or below the normal range (e.g., "elevated potassium," as opposed to "abnormal potassium"). If the laboratory abnormality can be characterized by a precise clinical term per standard definitions, the clinical term should be recorded as the AE. For example, an elevated serum potassium level of 7.0 mEq/L should be recorded as "hyperkalemia."

Observations of the same clinically significant laboratory abnormality from visit to visit should not be repeatedly recorded on the AE eCRF, unless the etiology changes. The initial severity of the event should be recorded, and the severity or seriousness should be updated any time the event worsens.

5.3.5.5 Abnormal Vital Sign Values

Not every vital sign abnormality qualifies as an AE. A vital sign result should be reported as an AE if it meets any of the following criteria:

- Accompanied by clinical symptoms
- Results in a change in study treatment (e.g., dosage modification, treatment interruption, or treatment discontinuation)
- Results in a medical intervention or a change in concomitant therapy
- Clinically significant in the investigator's judgment

It is the investigator's responsibility to review all vital sign findings. Medical and scientific judgment should be exercised in deciding whether an isolated vital sign abnormality should be classified as an AE.

If a clinically significant vital sign abnormality is a sign of a disease or syndrome (e.g., high blood pressure), only the diagnosis (i.e., hypertension) should be recorded on the AE eCRF.

Observations of the same clinically significant vital sign abnormality from visit to visit should not be repeatedly recorded on the AE eCRF, unless the etiology changes. The

initial severity of the event should be recorded, and the severity or seriousness should be updated any time the event worsens.

5.3.5.6 Hepatotoxicity

The finding of an increased serum ALT or AST ($>3\times$ULN) in combination with either an increased serum TBILI ($>2\times$ULN) or clinical jaundice in the absence of cholestasis or other causes of hyperbilirubinemia is considered to be an indicator of severe liver injury. Therefore, investigators must report as an AE the occurrence of either of the following:

- Treatment-emergent serum ALT or AST $>3\times$ULN in combination with serum TBILI $>2\times$ULN
- Treatment-emergent serum ALT or AST $>3\times$ULN in combination with clinical jaundice

The most appropriate diagnosis or (if a diagnosis cannot be established) the abnormal laboratory values should be recorded on the AE eCRF (see Section 5.3.5.1) and reported to the Sponsor within 24 hours after learning of the event, either as a SAE or a non-serious AE of special interest (see Section 5.4.2).

NRH, whether or not accompanied by liver laboratory abnormalities, should be reported to the Sponsor as a SAE.

5.3.5.7 Abnormal Left Ventricular Ejection Fraction

Symptomatic left ventricular systolic dysfunction (otherwise referred to as heart failure) should be reported as an SAE. If the diagnosis is heart failure it should be reported as such and not as individual signs and symptoms thereof.

Heart failure should be graded according to NCI-CTCAE v 4.0 for "heart failure" (Grade 2, 3, 4 or 5) and in addition according to the NYHA classification.

Heart failure occurring during the study and up to 10 years after last administration of study medications must be reported irrespective of causal relationship and followed until one of the following occurs: resolution or improvement to baseline status, no further improvement can be expected, or death.

Asymptomatic Left Ventricular Systolic Dysfunction

Asymptomatic declines in LVEF should generally not be reported as AEs since LVEF data are collected separately in the eCRF. Exceptions to this rule are as follows:

- An asymptomatic decline in LVEF $\geq$10 percentage-points from baseline to an LVEF $<$50% must be reported as an AE with the term of ‘ejection fraction decreased’ as per NCI-CTCAE v4.0 and, in addition, a comment in the AE comments field should confirm that this was asymptomatic.

- An asymptomatic decline in LVEF requiring treatment delay or leading to discontinuation of trastuzumab emtansine or trastuzumab must also be reported.

5.3.5.8 Deaths

For this protocol, mortality is an efficacy endpoint. Deaths that occur during the protocol-specified AE reporting period (see Section 5.3.1) that are attributed by the investigator solely to recurrence or progression of breast cancer should be recorded only on the Study Completion/Discontinuation eCRF. All other on-study deaths, regardless of relationship to study drug, must be recorded on the AE eCRF and immediately reported to the Sponsor (see Section 5.4.2). An independent monitoring committee will monitor the frequency of deaths from all causes.

Death should be considered an outcome and not a distinct event. The event or condition that caused or contributed to the fatal outcome should be recorded as the single medical concept on the AE eCRF. Generally, only one such event should be reported. The term **"sudden death"** should only be used for the occurrence of an abrupt and unexpected death due to presumed cardiac causes in a patient with or without preexisting heart disease, within 1 hour of the onset of acute symptoms or, in the case of an unwitnessed death, within 24 hours after the patient was last seen alive and stable. If the cause of death is unknown and cannot be ascertained at the time of reporting, **"unexplained death"** should be recorded on the AE eCRF. If the cause of death later becomes available (e.g., after autopsy), "unexplained death" should be replaced by the established cause of death.

During post-study treatment survival follow-up, deaths attributed to recurrence or progression of breast cancer should be recorded only on the Survival eCRF.

5.3.5.9 Preexisting Medical Conditions

A preexisting medical condition is one that is present at the screening visit for this study. Such conditions should be recorded on the General Medical History and Baseline Conditions eCRF.

A preexisting medical condition should be recorded as an AE only if the frequency, severity, or character of the condition worsens during the study. When recording such events on the AE eCRF, it is important to convey the concept that the preexisting condition has changed by including applicable descriptors (e.g., "more frequent headaches").

5.3.5.10 Lack of Efficacy or Worsening of Breast Cancer

Events that are clearly consistent with the expected pattern of recurrence or progression of the underlying disease should not be recorded as AEs. These data will be captured as efficacy assessment data only. In most cases, the expected pattern of recurrence or progression will be based on radiologic and biopsy criteria. In rare cases, the determination of clinical progression will be based on symptomatic deterioration. However, every effort should be made to document recurrence or progression using objective criteria. If there is any uncertainty as to whether an event is due to disease recurrence or progression, it should be reported as an AE.

5.3.5.11 Hospitalization or Prolonged Hospitalization

Any AE that results in hospitalization or prolonged hospitalization should be documented and reported as a SAE (per the definition of SAE in Section 5.2.2), except as outlined below.

The following hospitalization scenarios are not considered to be SAEs:

- Hospitalization for respite care
- Planned hospitalization required by the protocol (e.g., for study drug administration or insertion of access device for study drug administration)
- Hospitalization for a preexisting condition, provided that all of the following criteria are met:

    The hospitalization was planned prior to the study or was scheduled during the study when elective surgery became necessary because of the expected normal progression of the disease

    The patient has not suffered an AE

- Hospitalization due solely to recurrence or progression of the underlying cancer

5.3.5.12 Overdoses

Study drug overdose is the accidental or intentional use of the drug in an amount higher than the dose being studied. An overdose or incorrect administration of study drug is not an AE unless it results in untoward medical effects.

Any study drug overdose or incorrect administration of study drug should be noted on the Study Drug Administration eCRF.

All AEs associated with an overdose or incorrect administration of study drug should be recorded on the AE eCRF. If the associated AE fulfills serious criteria, the event should be reported to the Sponsor within 24 hours after learning of the event (see Section 5.4.2).

5.3.5.13 Patient-Reported Outcome Data

AE reports will not be derived from PRO data. However, if any patient responses suggestive of a possible AE are identified during site review of the PRO questionnaires,

site staff will alert the investigator, who will determine if the criteria for an AE have been met and will document the outcome of this assessment in the patient's medical record per site practice. If the event meets the criteria for an AE, it will be reported on the AE eCRF.

5.4 IMMEDIATE REPORTING REQUIREMENTS FROM INVESTIGATOR TO SPONSOR

The investigator must report the following events to the Sponsor within 24 hours after learning of the event, regardless of relationship to study drug:

- SAEs
- Non-serious AEs of special interest (refer to Section 5.2.3)
- Pregnancies

The investigator must report new significant follow-up information for these events to the Sponsor within 24 hours after becoming aware of the information. New significant information includes the following:

- New signs or symptoms or a change in the diagnosis
- Significant new diagnostic test results
- Change in causality based on new information
- Change in the event’s outcome, including recovery
- Additional narrative information on the clinical course of the event

Investigators must also comply with local requirements for reporting serious AEs to the local health authority and IRB/EC.

5.4.1 Emergency Medical Contacts

To ensure the safety of study patients, an Emergency Medical Call Center Help Desk will access the Roche Medical Emergency List, escalate emergency medical calls, provide medical translation service (if necessary), connect the investigator with a Roche Medical Monitor, and track all calls. The Emergency Medical Call Center Help Desk will be available 24 hours per day, 7 days per week. Toll-free numbers for the Help Desk and Medical Monitor contact information will be distributed to all investigators (see "Protocol Administrative and Contact Information & List of Investigators").

5.4.2 Reporting Requirements for Serious Adverse Events and Non-Serious Adverse Events of Special Interest

For reports of SAEs and non-serious AEs of special interest, investigators should record all case details that can be gathered within 24 hours on the AE eCRF and submit the report via the electronic data capture (EDC) system. A report will be generated and sent to Roche Safety Risk Management by the EDC system.

In the event that the EDC system is unavailable, a paper SAE/Non-Serious AE of Special Interest CRF and Fax Coversheet should be completed and faxed to Roche Safety Risk Management or its designee within 24 hours after learning of the event, using the fax numbers provided to investigators (see "Protocol Administrative and Contact Information & List of Investigators"). Once the EDC system is available, all information will need to be entered and submitted via the EDC system.

5.4.3 <u>Reporting Requirements for Pregnancies</u>

5.4.3.1 Pregnancies in Female Patients

Female patients of childbearing potential will be instructed to immediately inform the investigator if they become pregnant during the study or within 6 months after the last dose of study drug. A Pregnancy Report eCRF should be completed by the investigator within 24 hours after learning of the pregnancy and submitted via the EDC system. A pregnancy report will automatically be generated and sent to Roche Safety Risk Management. Pregnancy should not be recorded on the AE eCRF. The investigator should counsel the patient, discussing the risks of the pregnancy and the possible effects on the fetus. Monitoring of the patient should continue until conclusion of the pregnancy.

In the event that the EDC system is unavailable, a Pregnancy Report worksheet and Pregnancy Fax Coversheet should be completed and faxed to Roche Safety Risk Management or its designee within 24 hours after learning of the pregnancy, using the fax numbers provided to investigators (see "Protocol Administrative and Contact Information & List of Investigators").

5.4.3.2 Pregnancies in Female Partners of Male Patients

Male patients will be instructed through the Informed Consent Form to immediately inform the investigator if their partner becomes pregnant during the study or within 9 months after the last dose of study drug. A Pregnancy Report eCRF should be completed by the investigator within 24 hours after learning of the pregnancy and submitted via the EDC system. Attempts should be made to collect and report details of the course and outcome of any pregnancy in the partner of a male patient exposed to study drug. The pregnant partner will need to sign an Authorization for Use and Disclosure of Pregnancy Health Information to allow for follow-up on her pregnancy. Once the authorization has been signed, the investigator will update the Pregnancy Report eCRF with additional information on the course and outcome of the pregnancy. An investigator who is contacted by the male patient or his pregnant partner may provide information on the risks of the pregnancy and the possible effects on the fetus, to support an informed decision in cooperation with the treating physician and/or obstetrician.

In the event that the EDC system is unavailable, follow reporting instructions provided in Section 5.4.3.1.

5.4.3.3 Abortions

Any spontaneous abortion should be classified as a serious AE (as the Sponsor considers spontaneous abortions to be medically significant events), recorded on the AE eCRF, and reported to the Sponsor within 24 hours after learning of the event (see Section 5.4.2).

5.4.3.4 Congenital Anomalies/Birth Defects

Any congenital anomaly/birth defect in a child born to a female patient or female partner of a male patient exposed to study drug should be classified as a SAE, recorded on the AE eCRF, and reported to the Sponsor within 24 hours after learning of the event (see Section 5.4.2).

5.5 FOLLOW-UP OF PATIENTS AFTER ADVERSE EVENTS

5.5.1 Investigator Follow-Up

The investigator should follow each AE until the event has resolved to baseline grade or better, the event is assessed as stable by the investigator, the patient is lost to follow up, or the patient withdraws consent. Every effort should be made to follow all serious AEs considered to be related to study drug or trial-related procedures until a final outcome can be reported.

During the study period, resolution of AEs (with dates) should be documented on the AE eCRF and in the patient's medical record to facilitate source data verification. If, after follow-up, return to baseline status or stabilization cannot be established, an explanation should be recorded on the AE eCRF.

All pregnancies reported during the study should be followed until pregnancy outcome. If the EDC system is not available at the time of pregnancy outcome, follow reporting instructions provided in Section 5.4.3.1.

5.5.2 Sponsor Follow-Up

For SAEs, non-serious AEs of special interest, and pregnancies, the Sponsor or a designee may follow up by telephone, fax, electronic mail, and/or a monitoring visit to obtain additional case details and outcome information (e.g., from hospital discharge summaries, consultant reports, autopsy reports) in order to perform an independent medical assessment of the reported case.

5.6 POST-STUDY ADVERSE EVENTS

At the study treatment completion/early termination visit, the investigator should instruct each patient to report to the investigator any subsequent AEs that the patient's personal physician believes could be related to prior study drug treatment or study procedures.

The investigator should notify the Sponsor of any death, SAE, or other AE of concern occurring at any time after a patient has discontinued study treatment or study participation if the event is believed to be related to prior study drug treatment or study

procedures. The Sponsor should also be notified if the investigator becomes aware of the development of cancer or a congenital anomaly/birth defect in a subsequently conceived offspring of a patient that participated in this study.

The investigator should report these events to Roche Safety Risk Management on the AE eCRF. If the AE eCRF is no longer available, the investigator should report the event directly to Roche Safety Risk Management via telephone (see "Protocol Administrative and Contact Information & List of Investigators").

During post-study treatment survival follow-up, deaths attributed to recurrence or progression of breast cancer should be recorded only on the Survival eCRF.

5.7 EXPEDITED REPORTING TO HEALTH AUTHORITIES, INVESTIGATORS, INSTITUTIONAL REVIEW BOARDS, AND ETHICS COMMITTEES

To determine reporting requirements for single AE cases, the Sponsor will assess the expectedness of these events using the IB as a reference.

The Sponsor will compare the severity of each event and the cumulative event frequency reported for the study with the severity and frequency reported in the applicable reference document.

Reporting requirements will also be based on the investigator's assessment of causality and seriousness, with allowance for upgrading by the Sponsor as needed.

A DMC will monitor the incidence of these expected events during the study. An aggregate report of any clinically relevant imbalances that do not favor the test product will be submitted to health authorities.

6. STATISTICAL CONSIDERATIONS AND ANALYSIS PLAN

6.1 DETERMINATION OF SAMPLE SIZE

The sample size of the study is primarily driven by the analysis of IDFS. To detect a HR of 0.75 in IDFS (a 6.5% improvement in 3-year IDFS from 70% in the control arm to 76.5% in the trastuzumab emtansine arm), approximately 384 IDFS events will be required to achieve 80% power at a 2-sided significance level of 5%. Approximately 1484 patients will be enrolled in the study.

The study is expected to be fully enrolled around 35 months after the first patient enrolls in the study (FPI). The final IDFS analysis will be performed after approximately 384 events have occurred, which is projected to be approximately 64 months from FPI.

With the study sample size of 1484 patients and approximately 10 years of follow-up, this study has about 56% power to detect an HR of 0.8 (a 2.8% improvement in 3-year

OS from 85% in the control arm to 87.8% in the trastuzumab emtansine arm) at a 2-sided significance level of 5%.

6.2 SUMMARIES OF CONDUCT OF STUDY

Patient enrollment will be tabulated by study site for each treatment arm. Patient disposition and reasons for discontinuations will be summarized by treatment arm for all randomized patients. Compliance with protocol-specified schedule of disease status clinical assessments will also be summarized by treatment arm. In addition, protocol deviations and eligibility violations will be summarized by treatment arm.

6.3 SUMMARIES OF TREATMENT GROUP COMPARABILITY

The evaluation of treatment group comparability between the 2 treatment arms will include summaries of demographics and baseline characteristics, including age, sex, race, breast cancer characteristics, medical history, and prior cancer treatment. Descriptive statistics (mean, median, standard deviation, 25th percentile, 75th percentile, and range) will be presented for continuous variables, and proportions will be presented for categorical variables.

6.4 EFFICACY ANALYSES

The randomized patient population will form the basis for all efficacy analyses. In all efficacy analyses, following the intent-to-treat principle, patients will be included in the treatment group to which they are randomized by the IVRS/IWRS.

6.4.1 Primary Efficacy Endpoint

The primary efficacy variable is IDFS, defined as the time between randomization and date of first occurrence of an IDFS event (as described in Section 3.4.1). Patients who have not had an event will be censored at the date they are last known to be alive and event free on or prior to the clinical data cutoff date.

The log-rank test, stratified by the protocol-defined stratification factors (clinical stage at presentation [inoperable vs. operable]; hormone receptor status [ER or PR positive vs. ER and PR negative/unknown]; preoperative HER2-directed therapy [trastuzumab vs. trastuzumab plus additional HER2-directed agent(s)]; and pathologic nodal status evaluated after preoperative therapy [node positive vs. node negative/not done]), will be used to compare IDFS between the 2 treatment arms. The unstratified log-rank test results will also be provided as a sensitivity analysis. Cox proportional hazards model, stratified by the protocol-defined stratification factors, will be used to estimate the HR between the 2 treatment arms and its 95% CI. The Kaplan-Meier approach will be used to estimate 3-year IDFS rates and corresponding 95% CIs for each treatment arm.

6.4.2 Secondary Efficacy Endpoints

Secondary endpoints include IDFS including second primary non-breast cancer; DFS, OS, and DRFI (as defined in Section 3.4.2). Patients who have not had an event will be

censored at the date that they are last known to be event free on or prior to the clinical data cutoff date.

Secondary endpoints will be analyzed in a similar manner as the primary endpoint to estimate 3-year event rates (and 5-year survival rate for OS) for each treatment arm and the HR between the 2 treatment arms with 95% CI.

A testing hierarchy will be used to control the overall type I error rate at 5%. If the primary endpoint IDFS reaches statistical significance, the formal hypothesis testing of OS will be performed. More details of OS interim analyses are specified in Section 6.9.

6.5 SAFETY ANALYSES

Patients who receive any amount of study treatment will be included in the safety analyses. Safety results will be summarized based on the treatment patients actually receive. Specifically, a patient will be included in the trastuzumab emtansine arm in safety analyses if the patient receives any trastuzumab emtansine.

The safety of trastuzumab emtansine will be assessed through treatment exposure, summaries of AEs, SAEs, cardiac-specific AEs, LVEF measurements, and laboratory test results (including thrombocytopenia and transaminases).

Study treatment exposure, such as treatment duration, number of cycles, dose intensity, and dose modification (including dose delay, dose reduction, etc.) will be summarized for each treatment arm with descriptive statistics. Reasons for treatment discontinuation will also be summarized.

Verbatim descriptions of AEs will be mapped to Medical Dictionary for Regulatory Activities (MedDRA) thesaurus terms and graded according to the NCI CTCAE, Version 4.0. All AEs, SAEs, AEs leading to death, and AEs leading to study treatment discontinuation, occurring on or after the first dose of study treatment (i.e., treatment-emergent AEs), will be summarized by NCI CTCAE grade. For repeated events of varying severity in an individual patient, the highest grade will be used in the summaries. Deaths and causes of death will be summarized.

Laboratory toxicities will be summarized by NCI CTCAE grade for each treatment arm with shift tables.

Incidence of cardiac events, defined as death from cardiac cause or severe CHF (NYHA Class III or IV) with a decrease in LVEF of 10 percentage points or more from baseline to an LVEF of $<$ 50%, will be summarized by treatment arm. Other cardiac-related events (e.g., any symptomatic CHF associated with a 10% drop in LVEF to $<$ 50%; asymptomatic declines in LVEF requiring dose delay) will also be summarized. Change in LVEF over time will be summarized by treatment arm.

Incidence of hepatotoxicity events will be summarized by treatment arm. Additional analyses of liver function laboratory tests (LFTs) will also be performed.

For patients who continue on trastuzumab after discontinuation of trastuzumab emtansine due to toxicity before 14 cycles, exploratory safety analyses will be performed. Summary of trastuzumab exposure, SAEs, cardiac-specific AEs, and NCI CTCAE Grade 3 or above AEs will be provided. Other exploratory safety analyses differentiating 2 treatment phases for these patients will be performed as approximate.

6.6 PATIENT-REPORTED OUTCOME ANALYSES

HRQOL data will be captured using the following questionnaires: the EORTC QLQ-C30, the breast cancer module QLQ-BR23, and the EQ-5D.

Summary statistics (mean, standard deviation, median, 25th and 75th percentiles, and range) of absolute scores of the QLQ-C30 and QLQ-BR23 scales and their changes from baseline will be summarized at each assessment time point for the 2 treatment arms. Only patients with a baseline assessment and at least one post-baseline assessment will be included in this analysis. Repeated-measures mixed-effects models will be used to explore the treatment effect changes over time and treatment-by-time interaction.

The EuroQol EQ-5D is a five-item questionnaire with three categories (no problem, moderate problem, severe problems). Scoring and analysis will be reported in a separate document. The EQ-5D data analysis will be performed to support reimbursement dossiers and will not be included in the clinical study report (CSR).

6.7 PHARMACOKINETIC ANALYSES

Blood and serum samples for measurement of trastuzumab emtansine, total trastuzumab, and DM1 will be obtained from patients randomized to the trastuzumab emtansine arm. Individual and mean trastuzumab emtansine and total trastuzumab serum levels and DM1 plasma concentrations versus time data will be plotted, tabulated, and summarized (e.g., mean, standard deviation, coefficient of variation, median, minimum, maximum, and range). Interpatient variability and drug accumulation after multiple dosing will be evaluated. Compartmental, noncompartmental, and/or population approaches will be considered as appropriate. Additional PK and pharmacodynamic analyses and exposure-efficacy and exposure-safety (ALT/AST, platelet, etc.) analyses will be conducted in conjunction with analyses of data from other studies, as appropriate. Any remaining plasma samples may be used for measurement of trastuzumab emtansine metabolites as an exploratory assessment, and the results will be plotted, tabulated, and summarized.

Blood and serum samples for measurement of trastuzumab will be obtained from patients randomized to the trastuzumab arm. Individual and mean trastuzumab serum concentrations versus time data will be plotted, tabulated, and summarized (e.g., mean, standard deviation, coefficient of variation, median, minimum, maximum, and range).

6.8 EXPLORATORY ANALYSES

Exploratory analyses will be performed to explore the correlation between biomarker, ATA, and clinical outcomes as appropriate.

6.9 INTERIM ANALYSES

6.9.1 Interim Efficacy Analyses

One interim analysis of IDFS and 3 interim analyses of OS are planned.

The interim efficacy analysis of IDFS is planned after 67% of the targeted IDFS events have occurred, which is estimated to be approximately 48 months after the first patient is enrolled in the study. If the accrual rate or event rate are different from expected, the timing of the interim analysis may be delayed such that the interim analysis will only take place after all patients have enrolled and have completed treatment.

At this interim analysis, IDFS will be tested at the significance level determined using the Lan–DeMets alpha spending function with an O'Brien–Fleming boundary so that the overall 2-sided type I error rate will be maintained at the 5% level for the IDFS primary endpoint.

Table 5 presents a summary of the planned IDFS analyses, the efficacy stopping boundary, and the estimated timing of these analyses.

Table 5 Summary of Planned Analyses of Invasive Disease-Free Survival

Analysis of IDFS	No. of events	Efficacy Stopping Boundary [a]	Estimated Timing [b]
Interim	257	$p < 0.0124$ or observed HR < 0.732	48 months
Final	384	$p < 0.0462$ or observed HR < 0.816	64 months

HR = hazard ratio; IDFS = invasive disease-free survival.

[a] p-value will be based on 2-sided stratified log-rank test.

[b] Time from the enrollment of first patient to data cutoff.

The interim analysis will be performed by the iDCC statistician and the results will be presented to the iDMC by the iDCC statistician.

The purpose of the interim analysis is to evaluate whether there is an overwhelming difference in the efficacy observed in the trastuzumab emtansine arm compared with the trastuzumab arm in terms of IDFS. If the test is not significant, the study will continue as planned. If the test is significant, the iDMC may recommend releasing the primary endpoint results before the targeted number of 384 events is reported. In this latter situation, the Sponsor will be unblinded to the study results and a full data package would be prepared for discussion with regulatory authorities. The study will continue

until 10 years of follow-up and IDFS analysis will be updated when 384 IDFS events have occurred. Three formal interim OS analyses and one final OS analysis are planned: the first OS interim analysis will be performed at the time of the interim IDFS analysis (approximately 48 months from FPI) if the interim IDFS analysis crosses the boundary; the second interim OS analysis will be performed at the time of the final IDFS analysis (approximately 64 months from FPI; in the case where the interim IDFS analysis crosses the boundary, the second OS interim analysis will be performed when 384 IDFS events have occurred), followed by the third OS interim analysis at approximately 2 years (88 months from FPI) after the second OS interim analysis. The final OS analysis will be performed at the end of 10 years of follow-up. The Sponsor will perform these analyses. A survival data sweep will be conducted prior to each analysis.

The overall type I error will be controlled at 0.05 for the formal OS interim analyses and final OS analysis using the Lan–DeMets alpha spending function with an O'Brien–Fleming boundary. The boundaries used at each interim and final OS analysis will depend on the timing of the analyses and the number of death events actually included in the analyses.

Table 6 presents a summary of the planned OS analyses, the efficacy stopping boundary, and the estimated timing of these analyses.

Table 6 Summary of Planned Analyses of Overall Survival

Analysis Of OS	No. Of Events	Efficacy Stopping Boundary [a]	Estimated Timing [b]
Interim 1 (at interim IDFS)	150	$p < 0.0009$ or observed $HR < 0.5826$	48 months
Interim 2 (at final IDFS)	206	$p < 0.0053$ or observed $HR < 0.6785$	64 months
Interim 3	279	$p < 0.0184$ or observed $HR < 0.754$	88 months
Final	367	$p < 0.0435$ or observed $HR < 0.8099$	119 months

HR = hazard ratio; IDFS = invasive disease-free survival; OS = overall survival.

[a] p-value will be based on 2-sided stratified log-rank test.

[b] Time from the enrollment of first patient to data cutoff.

6.9.2 Interim Safety Analyses

An iDMC will monitor accumulating patient safety data at least once every 6 months until the last patient has completed study treatment. In addition, data on SAEs and deaths will be monitored by the iDMC at least once every 3 months during this period.

After the first 600 patients have been randomized and followed up for 3 months (approximately 21 months after FPI), the iDMC will perform an interim safety analysis regarding death and hepatic events. The Clinical Events Committee will communicate their findings regarding hepatic events to the iDMC to aid iDMC review.

If an absolute increase of > 3% in the percentage of death (from any cause) is observed in the trastuzumab emtansine arm compared with the trastuzumab arm, the iDMC will consider recommending holding enrollment for further data review, stopping, or modifying the trial.

If the true difference in the percentage of death is >3% (e.g., 2% vs. 6%) then there is approximately 70% chance of observing an absolute difference of >3% at the interim with 600 patients. Table 7 presents the probability of observing more than 3% increase in the percentage of death in trastuzumab emtansine arm compared with the trastuzumab arm with different assumption on the percentage of death in 2 arms.

Table 7 Probability of Observing > 3% Increase of Death

Percentage of death		
Trastuzumab (N = 300)	Trastuzumab emtansine (N = 300)	Probability of observing > 3% increase
2%	2%	0.00
2%	3%	0.05
2%	4%	0.20
2%	5%	0.45
2%	6%	0.70

If an absolute increase of > 3% in the percentage of Hy's law cases (confirmed by the independent clinical events committee) is observed in the trastuzumab emtansine arm compared with the control arm, the iDMC will consider recommending holding enrollment for further data review, stopping, or modifying the trial.

If the true difference in the percentage of confirmed Hy's law cases is > 3% (e.g., 0.33% vs. 3.67%) then there is approximately 54% chance of observing an absolute difference of > 3% at the interim with 600 patients. Table 8 presents the probability of observing > 3% increase in the percentage of Hy's law cases in the trastuzumab emtansine arm compared with the trastuzumab arm with different assumptions on the number of Hy's law cases in 2 arms.

Table 8 Probability of Observing > 3% Increase of Confirmed Hy's Law Cases

Number of confirmed Hy's law cases (%)		
Trastuzumab (N = 300)	Trastuzumab emtansine (N = 300)	Probability of observing > 3% increase
1 (0.33%)	4 (1.33%)	<0.01
1 (0.33%)	6 (2%)	0.05
1 (0.33%)	8 (2.67%)	0.19
1 (0.33%)	10 (3.33%)	0.43
1 (0.33%)	11 (3.67%)	0.54
1 (0.33%)	12 (4%)	0.66

The iDMC will work according to the guidelines defined in the iDMC Charter. The iDMC Charter will contain details regarding the frequency of meetings, guidelines for decision making, and process for requesting further information. The iDMC members will review and sign off on the charter before the first review.

7. DATA COLLECTION AND MANAGEMENT

7.1 DATA QUALITY ASSURANCE

The Sponsor will supply electronic eCRF specifications for this study. An academic research organization (NSABP) will be responsible for data management of this study, including quality checking of the data. Data entered manually will be collected via EDC using eCRFs. Sites will be responsible for data entry into the EDC system. In the event of discrepant data, the NSABP will request data clarification from the sites, which the sites will resolve electronically in the EDC system.

The NSABP will produce a Data Quality Plan that describes the quality checking to be performed on the data.

The Sponsor will perform oversight of the data management of this study, including approval of the NSABP's data management plans and specifications. Data will be transferred electronically from the NSABP to the Sponsor, and the Sponsor's standard procedures will be used to handle and process the electronic transfer of these data.

eCRFs and correction documentation will be maintained in the EDC system's audit trail. System backups for data stored at the NSABP and records retention for the study data will be consistent with the NSABP's standard procedures.

Electronic patient-reported outcome (ePRO) data will be collected electronically. The device is designed for entry of data in a way that is attributable, secure, and accurate, in compliance with FDA regulations for electronic records (21 CFR Part 11). The ePRO device data are available for view access only via secure access. Only identified and

trained users may view the data, and their actions become part of the audit trail. The Sponsor will have view access only. System backups for data stored by the Sponsor and records retention for the study data will be consistent with the Sponsor's standard procedures.

7.2 ELECTRONIC CASE REPORT FORMS

eCRFs are to be completed using the NSABP-designated EDC system. Sites will receive training and a have access to a manual for appropriate eCRF completion. eCRFs will be submitted electronically to the Sponsor and should be handled in accordance with instructions from the Sponsor.

All eCRFs should be completed by designated, trained site staff. eCRFs should be reviewed and electronically signed and dated by the investigator or a designee.

7.3 ELECTRONIC PATIENT-REPORTED OUTCOME DATA

PRO data will be collected electronically. The data will be transmitted electronically to a centralized database at the ePRO vendor. The data can be reviewed by site staff via secure access to a web server. Once the study is complete, the ePRO data, audit trail, and trial and system documentation will be archived. The investigator will receive patient data for the site in both human- and machine-readable formats on an archival-quality compact disc that must be kept with the study records as source data. Acknowledgement of receipt of the compact disc is required. In addition, the Sponsor will receive all patient data in a machine-readable format on a compact disc.

7.4 SOURCE DATA DOCUMENTATION

Study monitors will perform ongoing source data verification to confirm that critical protocol data (i.e., source data) entered into the eCRFs by authorized site personnel are accurate, complete, and verifiable from source documents.

Source documents (paper or electronic) are those in which patient data are recorded and documented for the first time. They include, but are not limited to, hospital records, clinical and office charts, laboratory notes, memoranda, PROs, evaluation checklists, pharmacy dispensing records, recorded data from automated instruments, copies of transcriptions that are certified after verification as being accurate and complete, microfiche, photographic negatives, microfilm or magnetic media, X-rays, patient files, and records kept at pharmacies, laboratories, and medico-technical departments involved in a clinical trial.

Before study initiation, the types of source documents that are to be generated will be clearly defined in the Trial Monitoring Plan. This includes any protocol data to be entered directly into the eCRFs (i.e., no prior written or electronic record of the data) and considered source data.

Source documents that are required to verify the validity and completeness of data entered into the eCRFs must not be obliterated or destroyed and must be retained per the policy for retention of records described in Section 7.6.

To facilitate source data verification, the investigators and institutions must provide the Sponsor direct access to applicable source documents and reports for trial-related monitoring, Sponsor audits, and IRB/EC review. The investigational site must also allow inspection by applicable health authorities.

7.5 USE OF COMPUTERIZED SYSTEMS

When clinical observations are entered directly into an investigational site's computerized medical record system (i.e., in lieu of original hardcopy records), the electronic record can serve as the source document if the system has been validated in accordance with health authority requirements pertaining to computerized systems used in clinical research. An acceptable computerized data collection system allows preservation of the original entry of data. If original data are modified, the system should maintain a viewable audit trail that shows the original data as well as the reason for the change, name of the person making the change, and date of the change.

7.6 RETENTION OF RECORDS

Records and documents pertaining to the conduct of this study and the distribution of IMP, including eCRFs, ePRO data, Informed Consent Forms, laboratory test results, and medication inventory records, must be retained by the Principal Investigator for at least 15 years after completion or discontinuation of the study, or for the length of time required by relevant national or local health authorities, whichever is longer. After that period of time, the documents may be destroyed, subject to local regulations.

No records may be disposed of without the written approval of the Sponsor. Written notification should be provided to the Sponsor prior to transferring any records to another party or moving them to another location.

8. ETHICAL CONSIDERATIONS

8.1 COMPLIANCE WITH LAWS AND REGULATIONS

This study will be conducted in full conformance with the ICH E6 guideline for Good Clinical Practice and the principles of the Declaration of Helsinki, or the laws and regulations of the country in which the research is conducted, whichever affords the greater protection to the individual. The study will comply with the requirements of the ICH E2A guideline (Clinical Safety Data Management: Definitions and Standards for Expedited Reporting). Studies conducted in the United States or under a U.S. Investigational New Drug (IND) application will comply with U.S. FDA regulations and applicable local, state, and federal laws. Studies conducted in the EU/EEA will comply with the EU Clinical Trial Directive (2001/20/EC).

8.2 INFORMED CONSENT

The Sponsor's sample Informed Consent Form (and ancillary sample Informed Consent Forms such as a Child's Assent or Caregiver's Informed Consent Form, if applicable) will be provided to each site. If applicable, it will be provided in a certified translation of the local language. The Sponsor or its designee must review and approve any proposed deviations from the Sponsor's sample Informed Consent Forms or any alternate consent forms proposed by the site (collectively, the "Consent Forms") before IRB/EC submission. The final IRB/EC-approved Consent Forms must be provided to the Sponsor for health authority submission purposes according to local requirements.

The Informed Consent Form will contain a separate section that addresses the use of remaining mandatory samples for optional exploratory research. The investigator or authorized designee will explain to each patient the objectives of the exploratory research. Patients will be told that they are free to refuse to participate and may withdraw their specimens at any time and for any reason during the storage period. A separate, specific signature will be required to document a patient's agreement to allow any remaining specimens to be used for exploratory research. Patients who decline to participate will check a "no" box in the appropriate section and will not provide a separate signature.

The Consent Forms must be signed and dated by the patient or the patient's legally authorized representative before his or her participation in the study. The case history or clinical records for each patient shall document the informed consent process and that written informed consent was obtained prior to participation in the study.

The Consent Forms should be revised whenever there are changes to study procedures or when new information becomes available that may affect the willingness of the patient to participate. The final revised IRB/EC-approved Consent Forms must be provided to the Sponsor for health authority submission purposes.

Patients must be re-consented to the most current version of the Consent Forms (or to a significant new information/findings addendum in accordance with applicable laws and IRB/EC policy) during their participation in the study. For any updated or revised Consent Forms, the case history or clinical records for each patient shall document the informed consent process and that written informed consent was obtained using the updated/revised Consent Forms for continued participation in the study.

A copy of each signed Consent Form must be provided to the patient or the patient's legally authorized representative. All signed and dated Consent Forms must remain in each patient's study file or in the site file and must be available for verification by study monitors at any time.

For sites in the United States, each Consent Form may also include patient authorization to allow use and disclosure of personal health information in compliance with the U.S.

Health Insurance Portability and Accountability Act of 1996 (HIPAA). If the site utilizes a separate Authorization Form for patient authorization for use and disclosure of personal health information under the HIPAA regulations, the review, approval, and other processes outlined above apply except that IRB review and approval may not be required per study site policies.

8.3 INSTITUTIONAL REVIEW BOARD OR ETHICS COMMITTEE

This protocol, the Informed Consent Forms, any information to be given to the patient, and relevant supporting information must be submitted to the IRB/EC by the Principal Investigator and reviewed and approved by the IRB/EC before the study is initiated. In addition, any patient recruitment materials must be approved by the IRB/EC.

The Principal Investigator is responsible for providing written summaries of the status of the study to the IRB/EC annually or more frequently in accordance with the requirements, policies, and procedures established by the IRB/EC. Investigators are also responsible for promptly informing the IRB/EC of any protocol amendments (see Section 9.5).

In addition to the requirements for reporting all AEs to the Sponsor, investigators must comply with requirements for reporting SAEs to the local health authority and IRB/EC. Investigators may receive written IND safety reports or other safety-related communications from the Sponsor. Investigators are responsible for ensuring that such reports are reviewed and processed in accordance with health authority requirements and the policies and procedures established by their IRB/EC, and archived in the site's study file.

8.4 CONFIDENTIALITY

The Sponsor maintains confidentiality standards by coding each patient enrolled in the study through assignment of a unique patient identification number. This means that patient names are not included in data sets that are transmitted to any Sponsor location.

Patient medical information obtained by this study is confidential and may only be disclosed to third parties as permitted by the Informed Consent Form (or separate authorization for use and disclosure of personal health information) signed by the patient, unless permitted or required by law.

Medical information may be given to a patient's personal physician or other appropriate medical personnel responsible for the patient's welfare, for treatment purposes.

Data generated by this study must be available for inspection upon request by representatives of the U.S. FDA and other national and local health authorities, Sponsor monitors, representatives, and collaborators, and the IRB/EC for each study site, as appropriate.

8.5 FINANCIAL DISCLOSURE

Investigators will provide the Sponsor with sufficient, accurate financial information in accordance with local regulations to allow the Sponsor to submit complete and accurate financial certification or disclosure statements to the appropriate health authorities. Investigators are responsible for providing information on financial interests during the course of the study and for 1 year after completion of the study (i.e., last patient, last visit).

9. STUDY DOCUMENTATION, MONITORING, AND ADMINISTRATION

9.1 STUDY DOCUMENTATION

The investigator must maintain adequate and accurate records to enable the conduct of the study to be fully documented, including but not limited to the protocol, protocol amendments, Informed Consent Forms, and documentation of IRB/EC and governmental approval. In addition, at the end of the study, the investigator will receive the patient data, which includes an audit trail containing a complete record of all changes to data.

9.2 SITE INSPECTIONS

Site visits will be conducted by the Sponsor or an authorized representative for inspection of study data, patients' medical records, and eCRFs. The investigator will permit national and local health authorities, Sponsor monitors, representatives, and collaborators, and the IRBs/ECs to inspect facilities and records relevant to this study.

9.3 ADMINISTRATIVE STRUCTURE

This study will have a Steering Committee (SC) that will provide guidance on the protocol and study design and the statistical analysis plan and will provide guidance on review of any relevant study-related documents or procedures in order to be confident that the data will be collected in a timely fashion and will be accurate and complete. A separate SC charter will outline the committee's composition, meeting timelines, and members' roles and responsibilities. Additionally, the SC will be kept apprised of all relevant efficacy and safety data from this and related clinical trials.

In addition, the study will have an iDMC and clinical events committee.

9.4 PUBLICATION OF DATA AND PROTECTION OF TRADE SECRETS

The results of this study may be published or presented at scientific meetings. If this is foreseen, the investigator agrees to submit all manuscripts or abstracts to the Sponsor prior to submission. This allows the Sponsor to protect proprietary information and to provide comments based on information from other studies that may not yet be available to the investigator.

The Sponsor will comply with the requirements for publication of study results. In accordance with standard editorial and ethical practice, the Sponsor will generally support publication of multicenter trials only in their entirety and not as individual center data. In this case, a coordinating investigator will be designated by mutual agreement.

Authorship will be determined by mutual agreement and in line with International Committee of Medical Journal Editors authorship requirements. Any formal publication of the study in which contribution of Sponsor personnel exceeded that of conventional monitoring will be considered as a joint publication by the investigator and the appropriate Sponsor personnel.

Any inventions and resulting patents, improvements, and/or know-how originating from the use of data from this study will become and remain the exclusive and unburdened property of the Sponsor, except where agreed otherwise.

9.5 PROTOCOL AMENDMENTS

Any protocol amendments will be prepared by the Sponsor. The Sponsor is responsible for promptly informing the IRB/EC of any amendments to the protocol. Approval must be obtained from the IRB/EC before implementation of any changes, except for changes necessary to eliminate an immediate hazard to patients or changes that involve logistical or administrative aspects only (e.g., change in Medical Monitor or contact information).

10. REFERENCES

1. Aaronson NK, Ahmedzai S, Bergman B, et al. The European Organization for Research and Treatment of Cancer QLQ-C30: a quality-of-life instrument for use in international clinical trials in oncology. J Natl Cancer Inst 1993;85:365–376.

2. Buzdar AU, Ibrahim NK, Francis D, et al. Significantly higher pathologic complete remission rate after neoadjuvant therapy with trastuzumab, paclitaxel, and epirubicin chemotherapy: results of a randomized trial in human epidermal growth factor receptor 2-positive operable breast cancer. J Clin Oncol. 2005;23:3676–3685.

3. Dang, C, Gianni, L, Romieu, G, et al. Cardiac Safety Data From a Phase 2 Study of Trastuzumab Emtansine (T-DM1) Following the Completion of Anthracycline-Based Chemotherapy as Adjuvant or Neoadjuvant Therapy for Early-Stage HER2-Positive Breast Cancer. ASCO 2012 abstract.

4. Fitzsimmons D, Johnson CD, George S, et al. Development of a disease specific quality of life (QoL) questionnaire module to supplement the EORTC core cancer QoL questionnaire, the QLQ-C30 in patients with pancreatic cancer. EORTC Study Group on Quality of Life. Eur J Cancer. 1999;35:939–41.

5. Gianni L, Eiermann W, Semiglazov V, et al. Neoadjuvant chemotherapy with trastuzumab versus neoadjuvant chemotherapy alone in patients with HER2-positive locally advanced breast cancer (the NOAH trial): a randomized controlled superiority trial with a parallel HER2-negative cohort. Lancet. 2010;375:377–384.

6. Gnant M, Harbeck N, Thomssen C. St. Gallen 2011: summary of the consensus discussion. Breast Care (Basel). 2011;6:136–141. Epub 2011 Apr 29.

7. Hurwitz, S, Dirix, L, Kocsis, J, et al. Trastuzumab emtansine (T-DM1) versus trastuzumab and docetaxel in previously untreated HER2-positive metastatic breast cancer (MBC): primary results of a randomized multicenter, open-label phase II study. ESMO, 2011.

8. Loibl, S, von Minckwitz, G, Blohmer, J, et al. Comparison of survival according to pathologic complete response (pCR) in patients with HER2-positive breast cancer receiving neoadjuvant chemotherapy with and without trastuzumab compared to patients with HER2-negative tumors. San Antonio Breast Cancer Symposium. Dec 2011.

9. Mauri D, Pavlidis N, Ioannidis J. Neoadjuvant versus adjuvant systemic treatment in breast cancer: a meta-analysis. J Natl Cancer Inst. 2005;97:188–194.

10. [NCCN] National Comprehensive Cancer Network®. NCCN Clinical Practice Guidelines in Oncology: Breast Cancer. Version 2.2011. Available at www.nccn.org.

11. Piccart-Gebhart, MJ, Procter, M, Leyland-Jones, B, et al. Trastuzumab after adjuvant chemotherapy in HER2-positive breast cancer. N Engl J Med. 2005; 353:1659-1672.

12. Rastogi P, Anderson S, Bear H, et al. Preoperative chemotherapy: updates of National Surgical Adjuvant Breast and Bowel Project Protocols B-18 and B-27. J Clin Oncol. 2008;26:778–785.

13. Romond E, Perez E, Bryant J, et al. Trastuzumab plus adjuvant chemotherapy for operable HER2-positive breast cancer. N Engl J Med. 2005;353:1673–1684.

14. Schneeweiss, A, Chia, S, Hickish, T, et al. Neoadjuvant pertuzumab and trastuzumab concurrent or sequential with an anthracycline-containing or concurrent with an anthracycline-free standard regimen: a randomized phase ii study (TRYPHAENA). San Antonio Breast Cancer Symposium. December 2011.

15. Slamon, D, Eiermann, W., Robert, N, et al. Adjuvant trastuzumab in HER2-positive breast cancer. N Engl J Med. 2011; 365:1273-1283.

16. Sprangers MA, Groenvold M, Arraras JI, et al. The European Organization for Research and Treatment of Cancer breast cancer-specific quality-of-life questionnaire module: first results from a three-country field study. J Clin Oncol. 1996;14:2756–2768.

17. Untch M, Fasching P, Konecny G, et al. Pathological complete response after neoadjuvant chemotherapy plus trastuzumab predicts favorable survival in HER2-overexpressing breast cancer: results from the TECHNO trial of the AGO and GBG study groups. J Clin Oncol. 2011.

Appendix 1 Schedule of Assessments

	Screening [a]	Cycles 1 and 2		Cycles 3–14		Study Drug Completion Visit [b]	Survival Follow-Up
Day	–30 to –1	1	14–21	1	14–21		
Informed consent [a]	x						
Assignment of patient numbers through IVRS/IWRS	x						
Tumor tissue submission for HER2 determination and exploratory biomarkers (mandatory)	x [c]						
Blood sample for plasma/serum biomarker analyses (optional)		x [d]		x [d]		x [d]	
Whole blood sample for genetic analyses (optional)		x [d]					
Medical history and demographics	x						
Complete physical examination	x						x [e]
Limited physical examination [f]		x		x		x	
Height [g]	x						
Vital signs [h]	x	x		x		x	
ECOG performance status	x					x	
Concomitant medication reporting	x [i]	x		x		x	x [e, j]
AE reporting [j]	x [k]	x		x		x	x
12-lead ECG	x						
ECHO/MUGA [l]	x		x [l]		x [l]	x [l]	x [l]
Disease status assessments [e]	x	x					x
Hematology [m]	x [1]	x [2]		x [2]		x	
Biochemistry [n]	x [1]	x [2]		x [2]		x	

Appendix 1 Schedule of Assessments (cont.)

	Screening [a]	Cycles 1 and 2		Cycles 3–14		Study Drug Completion Visit [b]	Survival Follow-Up
Day	–30 to –1	1	14–21	1	14–21		
PK samples (serum and plasma)		See Appendix 2 for details					
ATA assessment [o]		x [o]		x [o]		x [o]	x [o]
INR/aPTT	x [1]	As clinically indicated					
Pregnancy test [p]	x			x [p]		x	x
Bilateral mammogram	x (within 1 year)						x [q]
Patient-reported outcome assessment [r]	x			x [r]		x	x
Arm B: trastuzumab administration [s]		x		x			
Arm A: trastuzumab emtansine administration [s]		x		x			

AE = adverse event; ALK = alkaline phosphatase; ALT = alanine aminotransferase; aPTT = activated partial thromboplastin time; AST = aspartate aminotransferase; ATA = anti-therapeutic antibody; ECG = electrocardiogram; ECHO = echocardiogram; ECOG = Eastern Cooperative Oncology Group; INR = international normalized ratio; IVRS/IWRS = interactive voice response system/interactive web response system; MUGA = multiple-gated acquisition; PK = pharmacokinetics; TBILI = total bilirubin.

NOTE: Local laboratory (hematology, biochemistry, urinalysis, INR, and aPTT assessments) to be used for the following:

[1] Screening: to be performed within 7 days prior to randomization. Screening laboratory assessments may be done on the day of randomization and their results may be used for randomization visit purposes.

[2] Scheduled for Day 1 of Cycle 2 and beyond: to be performed within 72 hours preceding administration of study treatment; results must be reviewed and documented prior to administration of study treatment.

[a] Informed consent may be obtained at any time (including prior to the 30-day screening period) but must be obtained prior to the performance of any screening assessments. Results of screening tests or examinations performed as standard of care prior to obtaining informed consent and within 30 days prior to randomization may be used rather than repeating required tests.

[b] Performed within approximately 30 days after the last dose of study treatment.

[c] Tumor tissue samples (formalin-fixed paraffin-embedded [FFPE] material) obtained from the primary site before and after preoperative therapy are required for submission along with pathology reports. Paraffin-embedded tumor tissue block or a partial block from pretreatment material must be obtained. If sites are unable to send a tissue block due to local regulations, at least 8 unstained slides should be sent for HER2 testing, and in addition up to 5 slides for exploratory biomarker research. A tumor tissue block (FFPE) obtained at surgery after preoperative therapy must be submitted for exploratory biomarker analysis.

[d] If optional consent was given, serum and plasma samples will be collected for exploratory biomarker analyses and/or for long-term storage in the study's central biomarker repository for future biomarker analyses. Serum samples should be drawn at C1D1 baseline, C1D8, C4, C8, and C12, as well as at the study discontinuation visit.

[e] Disease status based on all available clinical assessments should be documented every 3 months during study treatment and up to 2 years, every 6 months from 3 to 5 years, and annually from 6 to 10 years. Whenever possible, disease recurrence should be confirmed pathologically. In cases of disease recurrence diagnosed at any time during the study, patients will be out of the study schedule and will be followed once a year (starting 1 year after first relapse) until Year 10 for survival, anti-cancer medications and new relapse events

[f] Limited symptom-directed physical exam focusing on organ systems related to a potential AE based on patient's interim medical history and/or existing AE profiles of the study drugs. Disease status based on all available clinical assessments should be documented every 3 months during study treatment.

[g] Height to be obtained at screening or at Cycle 1 Day 1 only.

[h] Vital signs should be obtained and reviewed but, aside from weight, are not required to be entered into the eCRF. Abnormal vital signs at any time during the course of study treatment should be recorded as AEs or SAEs if clinically significant.

[i] Record all prior anti-cancer therapies and concomitant medications.

[j] Patients will be followed for new or worsening AEs for 30 days following the last infusion of study drug or until the early termination visit, until treatment related AEs resolve or stabilize, or until the initiation of another anti-cancer therapy, whichever occurs first. After 30 days following last study treatment administration, the investigator should continue to follow all unresolved study-related AEs and SAEs until their resolution or stabilization, the patient is lost to follow-up, or it is determined that the study treatment or participation is not the cause of the AE/SAE. The investigator should notify the Sponsor of any death, SAE, or other AE of concern occurring at any time after a patient has discontinued study treatment or study participation if the event is believed to be related to prior study drug treatment or study procedures.

[k] During screening, only SAEs considered related to protocol-mandated procedures will be collected

[l] Cardiac monitoring (ECHO/MUGA) will be performed in all patients enrolled in the study. ECHO is the preferred method. The same method used for a given patient at screening should be used throughout the study. ECHO/MUGA should be obtained during the last week (Days 14–21) of C2, and every 4 cycles thereafter (C6, C10, C14). ECHO/MUGA should be obtained at the discontinuation visit if not performed within the previous 6 weeks and at 3, 6, 12, 18, 24, 36, 48, and 60 months of survival follow-up.

[m] Hematologic assessments include hemoglobin (Hb), hematocrit, red blood cell count, platelet count, and WBC, including determination of absolute neutrophil count (ANC).

[n] Biochemistry assessments at baseline include sodium, potassium, chloride, bicarbonate, glucose, blood urea nitrogen (BUN) or urea, creatinine, total and direct bilirubin, total protein, albumin, ALT, AST, and ALK. Assessments at each treatment and at study discontinuation include potassium, TBILI, ALT, AST, and ALK; other assessments may be obtained as clinically indicated.

[o] To be assessed in approximately 50% of patients at the following time points: pre-dose Cycle 1, Day 1 and Cycle 4, Day 1, study treatment termination, and at 3 months after the last study treatment. (See Appendix 2 for additional details.)

[p] Serum β-HCG test must be performed during screening. Urine β-HCG test may be performed at subsequent time points for women of childbearing potential (including pre-menopausal women who have had a tubal ligation) and for women not meeting the definition of postmenopausal. For all other women, documentation must be present in medical history confirming that the patient is not of childbearing potential. Urine pregnancy test in women of childbearing potential in all treatment arms every 3 cycles and at 3 and 6 months after the study completion visit. All positive urine pregnancy tests must be confirmed by a serum β-HCG test.

[q] Mammograms of any remaining breast tissue should be performed at least annually during follow-up.

[r] Patient-reported outcome (PRO) questionnaires should be completed before or upon arrival at the study site before any study-specific procedures are performed, and before the patient sees the physician, on Cycles 5 and 11, at the study drug completion visit, and every 6 months in follow up to month 24, and then annually up to 36 months.

[s] Patients who discontinue trastuzumab emtansine may complete the duration of their study therapy with trastuzumab, if appropriate, based on toxicity considerations. If so, they should perform the scheduled assessments as indicated for the study treatment period.

Appendix 2 Schedule of Pharmacodynamic and Pharmacokinetic Assessments

PK, PD, and ATA Assessments for T-DM1-Treated Patients [a]		
**Study Visit**	**Time**	**Sample Acquisition**
Cycle 1, Day 1 and Cycle 4, Day 1	Pre-T-DM1 infusion	• Serum sample for T-DM1 and total trastuzumab<br>• Plasma sample for DM1 [b]<br>• Serum sample for anti-T-DM1 antibody (ATA)
Cycle 1, Day 1 and Cycle 4, Day 1	15-30 min post-T-DM1 infusion	• Serum sample for T-DM1 and total trastuzumab<br>• Plasma sample for DM1 [b]
Cycle 1, Day 1 and Cycle 4, Day 1	2 hours (±15 min) post-T-DM1 infusion	• Serum sample for T-DM1 and total trastuzumab<br>• Plasma sample for DM1 [b]
Cycle 2, Day 1 and Cycle 5, Day 1	Pre-T-DM1 infusion	• Serum sample for T-DM1 and total trastuzumab
Study Treatment Termination	Any point during study visit	• Serum sample for T-DM1 and total trastuzumab<br>• Serum sample for anti-T-DM1 antibody (ATA)
3 months after last dose of T-DM1	Any point during study visit	• Serum sample for anti-T-DM1 antibody (ATA)

PK, PD, and ATA Assessments for Trastuzumab-Treated Patients[c]		
Cycle 1, Day 1 and Cycle 4, Day 1	Pre-trastuzumab infusion	• Serum sample for trastuzumab<br>• Serum sample for anti-trastuzumab antibody (ATA)
Cycle 1, Day 1 and Cycle 4, Day 1	15-30 min post-trastuzumab infusion	• Serum sample for trastuzumab
Study Drug Termination	Any point during study visit	• Serum sample for trastuzumab<br>• Serum sample for anti-trastuzumab antibody (ATA)
3 months after last dose of trastuzumab	Any point during study visit	• Serum sample for anti-trastuzumab antibody (ATA)

T-DM1 = trastuzumab emtansine.

[a] Samples collected in approximately 50% of T-DM1-treated patients.

[b] Any remaining plasma samples after DM1 analysis may be used for measurement of T-DM1 metabolites (e.g., MCC-DM1, Lys-MCC-DM1) as an exploratory assessment (if stability acceptable and at discretion of sponsor).

[c] Samples collected in approximately 50% of trastuzumab-treated patients.

Appendix 3 European Organisation for Research and Treatment of Cancer Quality of Life Questionnaire – Core 30

EORTC QLQ-C30 (version 3)

We are interested in some things about you and your health. Please answer all of the questions yourself by circling the number that best applies to you. There are no "right" or "wrong" answers. The information that you provide will remain strictly confidential.

Please fill in your initials:
Your birthdate (Day, Month, Year):
Today's date (Day, Month, Year):

		Not at All	A Little	Quite a Bit	Very Much
1.	Do you have any trouble doing strenuous activities, like carrying a heavy shopping bag or a suitcase?	1	2	3	4
2.	Do you have any trouble taking a long walk?	1	2	3	4
3.	Do you have any trouble taking a short walk outside of the house?	1	2	3	4
4.	Do you need to stay in bed or a chair during the day?	1	2	3	4
5.	Do you need help with eating, dressing, washing yourself or using the toilet?	1	2	3	4

During the past week:

		Not at All	A Little	Quite a Bit	Very Much
6.	Were you limited in doing either your work or other daily activities?	1	2	3	4
7.	Were you limited in pursuing your hobbies or other leisure time activities?	1	2	3	4
8.	Were you short of breath?	1	2	3	4
9.	Have you had pain?	1	2	3	4
10.	Did you need to rest?	1	2	3	4
11.	Have you had trouble sleeping?	1	2	3	4
12.	Have you felt weak?	1	2	3	4
13.	Have you lacked appetite?	1	2	3	4
14.	Have you felt nauseated?	1	2	3	4
15.	Have you vomited?	1	2	3	4
16.	Have you been constipated?	1	2	3	4

Please go on to the next page

ENGLISH

During the past week:	Not at All	A Little	Quite a Bit	Very Much
17. Have you had diarrhea?	1	2	3	4
18. Were you tired?	1	2	3	4
19. Did pain interfere with your daily activities?	1	2	3	4
20. Have you had difficulty in concentrating on things, like reading a newspaper or watching television?	1	2	3	4
21. Did you feel tense?	1	2	3	4
22. Did you worry?	1	2	3	4
23. Did you feel irritable?	1	2	3	4
24. Did you feel depressed?	1	2	3	4
25. Have you had difficulty remembering things?	1	2	3	4
26. Has your physical condition or medical treatment interfered with your family life?	1	2	3	4
27. Has your physical condition or medical treatment interfered with your social activities?	1	2	3	4
28. Has your physical condition or medical treatment caused you financial difficulties?	1	2	3	4

For the following questions please circle the number between 1 and 7 that best applies to you

29. How would you rate your overall health during the past week?

1	2	3	4	5	6	7
Very poor						Excellent

30. How would you rate your overall quality of life during the past week?

1	2	3	4	5	6	7
Very poor						Excellent

© Copyright 1995 EORTC Quality of Life Group. All rights reserved. Version 3.0

Appendix 4 European Organisation for Research and Treatment of Cancer Quality of Life Questionnaire – Breast Cancer 23

EORTC QLQ - BR23

Patients sometimes report that they have the following symptoms or problems. Please indicate the extent to which you have experienced these symptoms or problems during the past week.

During the past week:	Not at All	A Little	Quite a Bit	Very Much
31. Did you have a dry mouth?	1	2	3	4
32. Did food and drink taste different than usual?	1	2	3	4
33. Were your eyes painful, irritated or watery?	1	2	3	4
34. Have you lost any hair?	1	2	3	4
35. Answer this question only if you had any hair loss: Were you upset by the loss of your hair?	1	2	3	4
36. Did you feel ill or unwell?	1	2	3	4
37. Did you have hot flushes?	1	2	3	4
38. Did you have headaches?	1	2	3	4
39. Have you felt physically less attractive as a result of your disease or treatment?	1	2	3	4
40. Have you been feeling less feminine as a result of your disease or treatment?	1	2	3	4
41. Did you find it difficult to look at yourself naked?	1	2	3	4
42. Have you been dissatisfied with your body?	1	2	3	4
43. Were you worried about your health in the future?	1	2	3	4

During the past <u>four</u> weeks:	Not at All	A Little	Quite a Bit	Very Much
44. To what extent were you interested in sex?	1	2	3	4
45. To what extent were you sexually active? (with or without intercourse)	1	2	3	4
46. Answer this question only if you have been sexually active: To what extent was sex enjoyable for you?	1	2	3	4

Please go on to the next page

Appendix 4 European Organisation for Research and Treatment of Cancer Quality of Life Questionnaire – Breast Cancer 23 (cont.)

During the past week:	Not at All	A Little	Quite a Bit	Very Much
47. Did you have any pain in your arm or shoulder?	1	2	3	4
48. Did you have a swollen arm or hand?	1	2	3	4
49. Was it difficult to raise your arm or to move it sideways?	1	2	3	4
50. Have you had any pain in the area of your affected breast?	1	2	3	4
51. Was the area of your affected breast swollen?	1	2	3	4
52. Was the area of your affected breast oversensitive?	1	2	3	4
53. Have you had skin problems on or in the area of your affected breast (e.g., itchy, dry, flaky)?	1	2	3	4

Appendix 5 EuroQoL EQ-5D

EQ - 5D

Health Questionnaire

English version for the US

Appendix 5 EuroQoL EQ-5D (cont.)

By placing a checkmark in one box in each group below, please indicate which statements best describe your own health state today.

**Mobility**	
I have no problems in walking about	❑
I have some problems in walking about	❑
I am confined to bed	❑
**Self-Care**	
I have no problems with self-care	❑
I have some problems washing or dressing myself	❑
I am unable to wash or dress myself	❑
**Usual Activities** (*e.g. work, study, housework, family or leisure activities)*	
I have no problems with performing my usual activities	❑
I have some problems with performing my usual activities	❑
I am unable to perform my usual activities	❑
**Pain/Discomfort**	
I have no pain or discomfort	❑
I have moderate pain or discomfort	❑
I have extreme pain or discomfort	❑
**Anxiety/Depression**	
I am not anxious or depressed	❑
I am moderately anxious or depressed	❑
I am extremely anxious or depressed	❑
